# Association of albumin-bilirubin grade with survival outcomes in patients with cholangiocarcinoma

**DOI:** 10.1371/journal.pone.0321758

**Published:** 2025-05-07

**Authors:** Jing Ye, Rongqiang Liu, Jianguo Wang, Wangbin Ma, Chen Chen, Jia Yu, Weixing Wang

**Affiliations:** Department of General Surgery, Renmin Hospital of Wuhan University, Wuhan, China; Rutgers: Rutgers The State University of New Jersey, UNITED STATES OF AMERICA

## Abstract

**Background:**

Numerous studies have reported the association between preoperative albumin-bilirubin (ALBI) grade and survival outcomes in patients with cholangiocarcinoma (CCA). However, the results are inconsistent. Therefore, we conducted a meta-analysis to comprehensively evaluate the relationship between ALBI grade and prognosis of CCA patients.

**Methods:**

We retrieved the databases (Pubmed,Embase and Web of science) to search articles about the association of ALBI grade with prognostic value in CCA patients. Hazard ratios (HR) and 95% confidence intervals (CI) were used to summarize survival outcomes. STATA 12.0 was used to implement the data analyses.

**Results:**

11 studies from 10 articles comprising 2841 patients were included in the meta-analysis. The pooled results showed that high ALBI grade was significantly associated with poor overall survival (OS) (HR: 1.75, 95% CI: 1.32–2.31) and relapse-free survival(RFS)(HR:1.95;95%CI:1.37–2.13). Subgroup analysis further showed that high ALBI grade had better predictive value for Asian population(HR:1.92;95%CI:1.46–2.51). Sensitivity analysis indicated that the results of the meta-analysis were stable.

**Conclusions:**

High ALBI grade predicted adverse survival outcomes in CCA patients. ALBI grade may be an efficient and convenient prognostic indicator in CCA patients for Asian population.

## Introduction

Cholangiocarcinoma (CCA) is the most general biliary malignancy and the second most prevalent hepatic malignancy after hepatocellular carcinoma (HCC) [1]. CCA is a tumor originating from the epithelial cells of the bile duct and is classified into two types: intrahepatic and extrahepatic cholangiocarcinoma [2]. Surgical resection is the best therapeutic approach for CCA patients. Unfortunately, most patients are diagnosed in advanced stages and the 5-year overall survival rate is poor [3]. Therefore, there is a need for a reliable and easily accessible preoperative prognostic biomarker to identify high-risk patients.

Tumor burden, such as tumor number and tumor size has been associated with clinical outcomes in CCA patients [4,5]. Liver function is also critical in determining the outcome of CCA patients undergoing liver surgery [6–8]. Johnson et al. proposed a new liver function evaluation model called the albumin-bilirubin grade (ALBI grade) [9]. ALBI grade was composed of blood albumin and total bilirubin. ALBI grade were calculated as follows:ALBI = [log10bilirubin (μmol/L) * 0.66] + [albumin (g/L) * −0.085].According to the cut off value of ALBI, ALBI ≤ −2.70 was defined as low ALBI, while ALBI > −2.70 was considered high [9]. Subsequent studies showed that ALBI grade could not only effectively reflect the liver function of HCC patients, but also effectively predict the prognosis of HCC patients [10]. Many scholars applied ALBI grade for patients with CCA and found that ALBI grade had prognostic value in patients with CCA. Yang et al found that high ALBI grade portended poor overall survival time in intrahepatic cholangiocarcinoma(ICC) patients underwent ultrasound-guided percutaneous microwave ablation [11]. Li et al revealed that patients with high ALBI grade had shorter survival time than lower ALBI grade group after radical resection [12]. Several other studies expressed the same view [13,14]. However, Quan et al suggested that ALBI grade was not associated with prognosis in CCA patients, which was also supported by Azar and collaborators [15,16]. Therefore, the relationship between ALBI grade and prognosis in CCA patients remained uncertain. The previous meta-analyses confirmed that high ALBI grade was associated with poorer prognosis in patients with HCC undergoing transarterial chemoembolization therapy [17,18].In this study, we also used meta-analysis to explore the prognostic value of ALBI grade in CCA patients.

## Methods

### Search strategy

Three authors (Jing Ye, Rongqianng Liu and Jianguo Wang) independently searched electronic databases, including Pubmed, Embase and Web of science to identify relevant articles. Disagreements were resolved through discussion. The search deadline was March 10, 2024. The following keywords were used: “albumin–bilirubin grade” OR “ALBI” AND bile duct adenoma OR bile duct neoplasms OR bile duct cancer OR biliary tract cancer OR cholangiocarcinoma OR cholangiocellular carcinoma. Additionally, we manually screened the references of selected studies to obtain other relevant articles. This study did not require the approval of the ethics committee. The meta-analysis was conducted according to the PRISMA statement. Detailed information was displayed in PRISMA_2020_checklist.docx (S1 Checklist).

### Selection criteria

The inclusion criteria were as follows: (1) investigated the association between the ALBI grade and survival outcomes. (2) reported the hazard ratios (HRs) and 95% confidence intervals (CIs). (3) described the concrete treatment methods. Reviews, letters, conference abstracts, studies with insufficient data or studies with duplicated data were excluded.

### Data extraction and quality assessment

Two investigators independently performed data extraction. The extracted data included the first author’s name, publication year, country, sample size, age, cut-off value of ALBI, pathological type, analysis type, treatment method and survival outcomes. The quality of included studies was evaluated by two independent authors using the Newcastle-Ottawa Scale (NOS) [19]. Studies with NOS scores more than 6 were considered to be high-quality studies.

### Statistical analysis

We used STATA 12.0 statistical software (STATA, College Station) to implement meta-analysis. If the survival outcomes were directly described in the study, we used them directly. Otherwise, they were extracted from the survival curves according to the methods described by Tierney [20]. We assessed heterogeneity among the studies using I-squared [21]. If the I-squared was less than or equal to 50%, we would use a fixed-effects model. Otherwise, we would use a random-effects model. Subgroup analysis and meta-regression were conducted to explore the possible sources of heterogeneity. Additionally, sensitivity analysis was applied to test the stability of the results. Publication bias was detected through the Begg’s test and Egger’s test [22]. P values less than 0.05 was considered statistically significant.

## Result

### Search results

**Fig 1:**
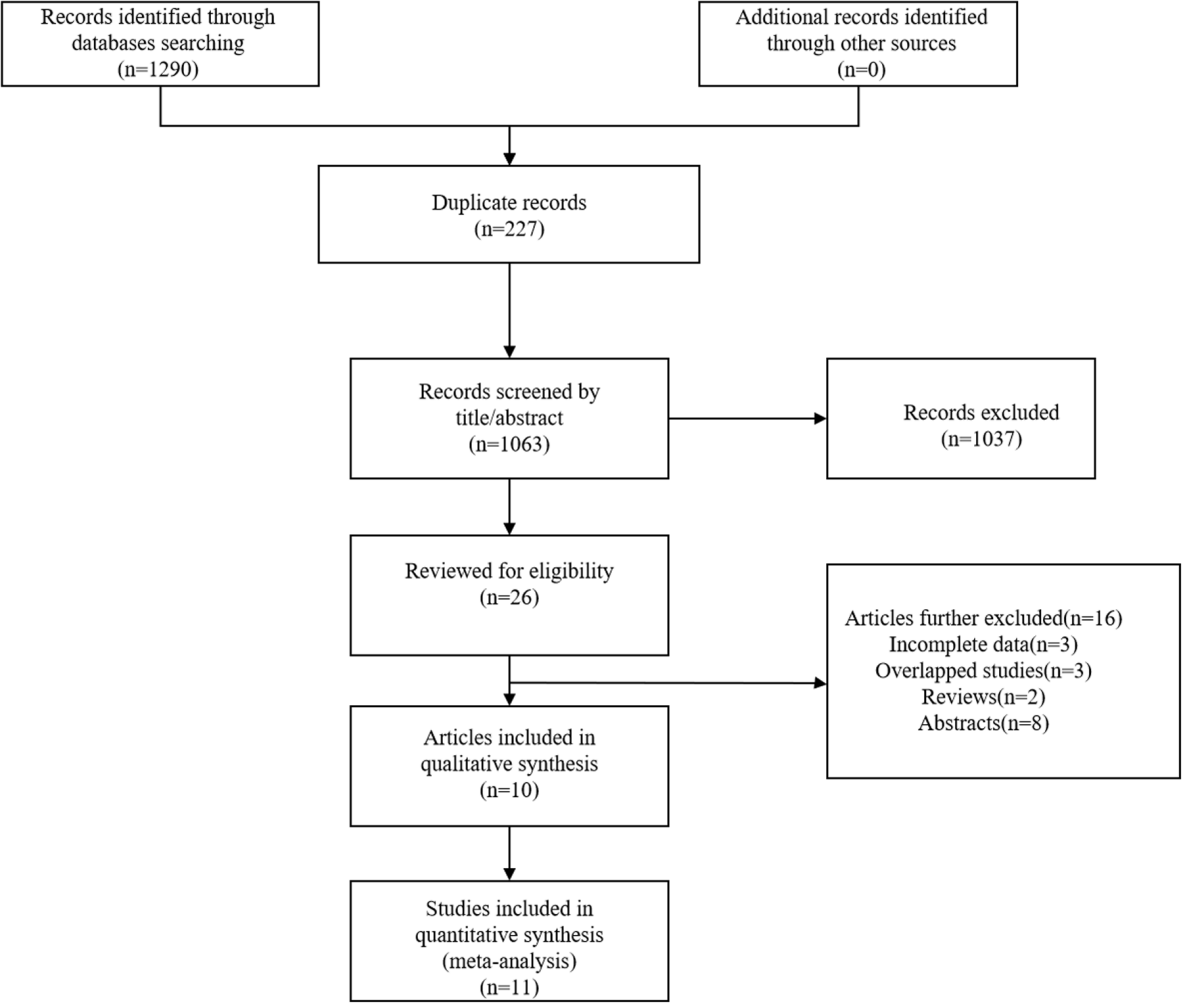
The flow diagram of identifying eligible studies.

A total of 1290 articles were initially identified. After removing duplicated studies, 1063 articles were remained for evaluation(S1 Table. specific information of 1063 articles). Based on the inclusion criteria, 1037 articles were further excluded. Subsequently, 26 articles were further evaluated. Since 1 article contained the experimental group and the validation group, 11 studies from 10 articles were included [11–16,23–26]. The study selection process was illustrated in the Fig 1.

### Study characteristics

Table 1 summarized the main characteristics of the included studies. 8 studies were conducted in China, 2 in the United States, and 1 from Japan. Among the included studies, 10 studies reported overall survival (OS) data, 3 studies displayed relapse-free survival (RFS) and 1 study showed post-recurrence survival (PRS) data. The NOS score of each study ranged from 6 to 8 (Table 2).

**Table 1:** Basic information of included studies.

Study	Year	Country	Study type	Sample size	Age	Cut-off of ALBI	Pathological type	Treatment methods	Analysis type	Survival analysis	Source of HR	NOS score
Kaneko	2021	Japan	R	83	Median 72	-2.6	iCCA	Mixed	Multivariate	OS	Reported	7
Li	2020A	China	R	477	Median 58	-2.6	iCCA	Surgery	Multivariate	OS	Reported	8
Li	2020B	China	R	143	Median 59	-2.6	iCCA	Surgery	Multivariate	OS	Reported	8
Ni	2019	China	R	78	59.6 ± 10	-2.6	iCCA	PMWA	Multivariate	OS.RFS	Reported	7
Wang	2018	China	R	109	68.9 ± 11.1	-2.6	EHC	PTBS combined with ^125^I	Multivariate	OS	Reported	7
Li	2021	China	R	535	Median 59	-2.6	iCCA	Surgery	Multivariate	OS,RFS	Reported	7
Yang	2021	China	R	52	59.6 ± 10	-2.6	iCCA	Surgery	Multivariate	OS,RFS	Reported	7
Azar	2020	USA	R	22	Mean 62.1	-2.6	iCCA	Radioembolization	Univariate	OS	SC	6
Quan	2022	China	R	289	NA	-2.6	CCA	Surgery	Multivariate	OS	Reported	7
Xing	2020	China	R	178	NA	-2.6	iCCA	Surgery	Univariate	PRS	Reported	6
Tsilimigras	2020	USA	R	885	Median 60.4	-2.6	iCCA	Surgery	Univariate	OS	Reported	7

**Abbreviation:** R, Retrospective; PTBS, percutaneous transhepatic biliary stenting; PMWA, percutaneous microwave ablation; OS, overall survival; RFS, recurrent-free survival; PRS: post-recurrence survival; SC, survival curve; iCCA, intrahepatic cholangiocarcinoma; CCA, cholangiocarcinoma; EHC, extrahepatic cholangiocarcinoma;

**Table 2:** Newcastle-Ottawa quality assessment scale.

First author	Year	Selection	Comparability	Outcome	Total
Kaneko	2021	★★★	★	★★★	7
Li	2020A	★★★	★★	★★★	8
Li	2020B	★★★	★★	★★★	8
Ni	2019	★★★	★★	★★★	8
Wang	2018	★★	★★	★★★	7
Li	2021	★★	★★	★★★	7
Yang	2021	★★	★★	★★★	7
Azar	2020	★★	★★	★★	6
Quan	2022	★★	★★	★★★	7
Xing	2020	★	★★	★★★	6
Tsilimigras	2020	★★	★★	★★★	7

### Relationship between high ALBI grade and OS

**Fig 2:**
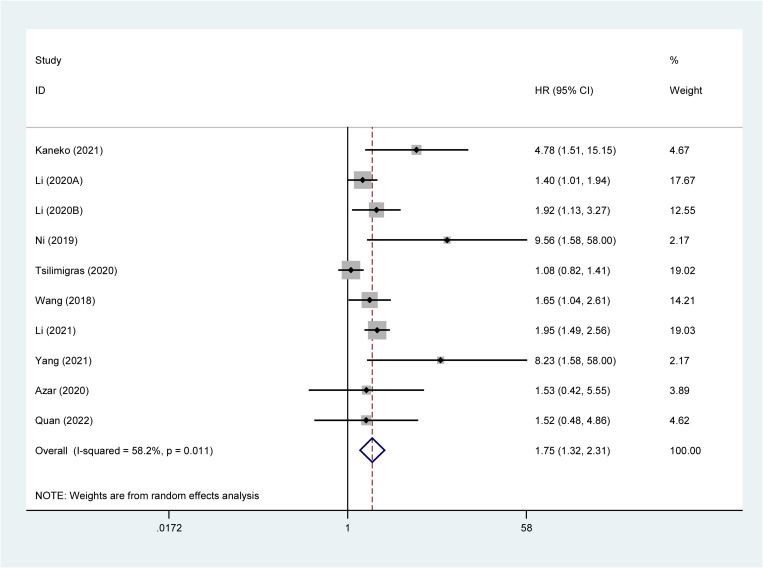
Relationship between high ALBI grade and OS. **Abbreviations:** ALBI, albumin-bilirubin grade; OS, overall survival; **Note:** The solid black line is called an invalid line. If the results are on this line, the study is not statistically significant. ‌The red dashed line represents the point estimate of the pooled outcome effect size. The diamonds represent the pooled results of the meta-analysis. The diamond is in the right of the black solid line, indicating that high ALBI grade predicts a poor prognosis. On the left side, high ALBI grade is favorable factor for prognosis. The intersection with the black solid line indicates that high ALBI grade is not associated with prognosis.

Due to significant heterogeneity (I^2^ = 58.2%), a random effects model was used. We found a significant correlation between high ALBI grade and poor OS (HR: 1.75, 95%CI: 1.32–2.31) (Fig 2).

### Subgroup analysis and meta-regression

We conducted subgroup analysis and meta-regression based on country, race, treatment method, sample size, analysis type, source of HR and data source (Table 3). The results revealed that high ALBI grade predicted poor prognosis regardless of surgical treatment group (HR:1.58;95%CI:1.17–2.15) or non-surgical treatment group (HR:2.02;95%CI:3.00), which suggested that the prognostic value of ALBI grade in CCA was not affected by the treatment methods. Regardless of sample size greater than or less than 200, high ALBI grade predicted poor OS. In terms of data source, high ALBI grade had the survival value in the multi-center group (HR:1.80, 95% CI:1.52–2.14). For different country, high ALBI grade was associated with adverse survival outcome in China group (HR:1.77, 95% CI:1.48–2.10) and Japan (HR:4.78, 95% CI:1.51–15.15). For different race, high ALBI grade predicted unfavorable survival outcome in the Asian population group(HR:1.92;95%CI:1.46–2.51), which displayed that the prognostic value of ALBI grade in CCA patients was more applicable for the Asian population. In addition, subgroup analysis showed that analysis type, country and race may contribute to heterogeneity. Meta-regression revealed that the data source could be a source of heterogeneity (P = 0.006).

**Table 3:** Subgroup analysis and meta regression for OS.

Variables	No. of studies	Estimate HR (95%)	P value	Heterogeneity		Meta-regression		
I^2^ (%)	P value	Tau^2^	Adj R^2^ (%)	P value
**Treatment methods**						0.07564	-12.88	0.099
Surgery	6	1.58(1.17–2.15)	0.003	63.4	0.018			
No-surgery	4	2.02(1.38–3.00)	<0.01	49.4	0.115			
**Sample size**						0.06464	3.35	0.824
≥200	4	1.44(1.05–1.99)	0.025	67.3	0.027			
<200	6	2.07 (1.51–2.83)	<0.01	39.6	0.142			
**Analysis type**						0.0165	46.66	0.183
Univariate analysis	2	1.10(0.84–1.43)	0.498	0	0.538			
Multivariate analysis	8	1.92(1.46–2.51)	<0.01	40.1	0.112			
**Source of HR**						0.07585	-13.18	0.476
Reported	9	1.77(1.32–2.38)	<0.01	62.8	0.006			
SC	1	1.53(0.42–5.55)						
**Data source**						0.007804	88.35	0.006
Multi-center	1	1.08(0.82–1.41)						
Single- center	9	1.80(1.52–2.14)	<0.01	31.9	0.163			
**Country**						0.01	81.6	0.11
China	7	1.77(1.48–2.10)	<0.01	32.4	0.18			
USA	2	1.10(0.84–1.43)	0.498	0	0.605			
Japan	1	4.78(1.51–15.15)						
**Race**						0.08	88.02	0.053
Caucasian	2	1.10(0.84–1.43)	0.498	0	0.605			
Asian	8	1.92(1.46–2.51)	<0.01	40.1	0.112			

### Relationship between high ALBI grade and RFS/PRS

**Fig 3:**
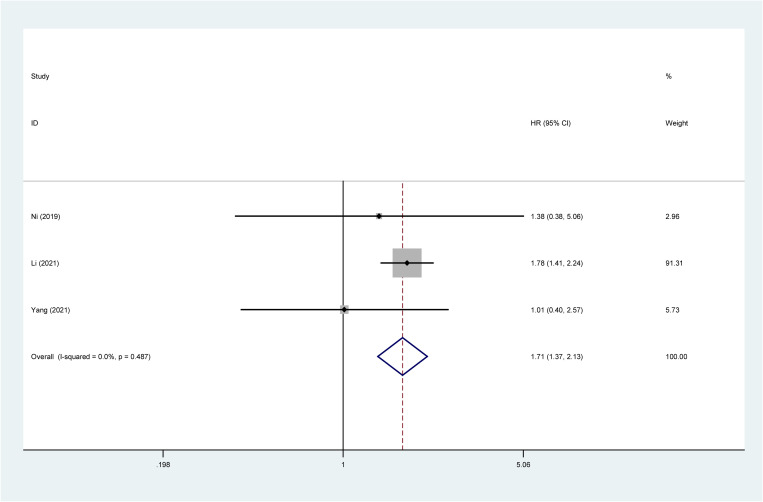
Relationship between high ALBI grade and RFS. **Abbreviations:** ALBI, albumin-bilirubin grade; RFS, recurrence-free survival; **Note:** The solid black line is called an invalid line. If the results are on this line, the study is not statistically significant. ‌The red dashed line represents the point estimate of the pooled outcome effect size. The diamonds represent the pooled results of the meta-analysis.The diamond is in the right of the black solid line, indicating that high ALBI grade predicts a poor prognosis. On the left side, high ALBI grade is favorable factor for prognosis. The intersection with the black solid line indicates that high ALBI grade is not associated with prognosis.

3 studies investigated the association between high ALBI grade and RFS. Comprehensive analysis revealed that patients with high ALBI grade had significantly worse RFS/PRS compared to those with low ALBI grade (HR: 1.71; 95% CI: 1.37–2.13) (Fig 3).

### Sensitivity analysis

**Fig 4:**
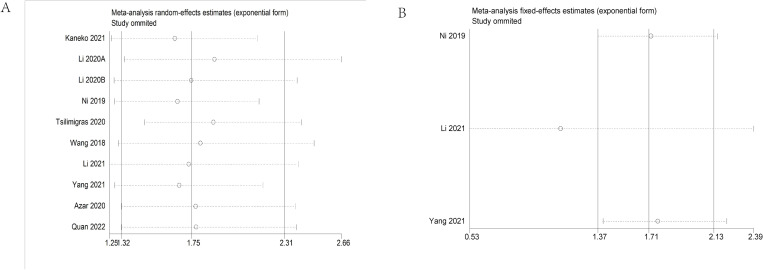
Sensitivity analysis. for OS. **(A)** Sensitivity analysis for OS. **(B)** Sensitivity analysis for RFS. **Abbreviations:** OS, overall survival; RFS, recurrence-free survival; **Note:**The x-axis represents the effect size.The black line in the middle represents the total combined effect size. The black lines on both sides indicate the 95% confidence interval range for the total effect size. After deleting one article, the combined results of the remaining studies were not statistically significant, indicating that the results of the original meta-analysis were not robust.

Sensitivity analysis was a crucial step in meta-analysis and can evaluate the reliability of the results by excluding one study in turn. Sensitivity analysis could help us to determine if the pooled analysis was heavily influenced by any single study. In this study, sensitivity analysis showed that the results of the meta-analysis were stable and reliable (Fig 4).

### Publication bias

**Fig 5:**
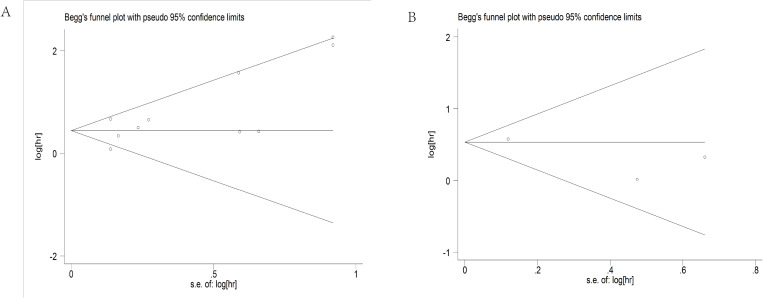
Publication bias. **(A)**Publication bias for OS. **(B)** Publication bias for RFS. **Abbreviations:** OS, overall survival; RFS, recurrence-free survival; **Note:**The x-axis represents the effect size and the y-axis represents the standard error of the logarithm of the effect size.The roughly symmetrical distribution of all points indicates that there is no publication bias.

Begg’s test and Egger’s test were used to evaluate possible publication bias. P values of Begg’s test and Egger’s test for OS were 0.243 and 0.061, respectively (Fig 5A). P values of Begg’s test and Egger’s test for RFS were 1 and 0.345, respectively (Fig 5B). P values were more than 0.05,which indicated that there was no publication bias.

## Discussion

The prognostic value of ALBI grade in HCC was demonstrated. However, the prognostic significance of ALBI grad in CCA was unclear. To our knowledge, the study was the first meta-analysis to explore the prognostic value of ALBI grade in CCA patients. A total of 11 studies including 2841 patients were enrolled. The results showed that high ALBI grade was associated with unfavorable OS and RFS. Subgroup analysis revealed that ALBI grade had predictive value in patients with early, middle and advanced CCA patients. Immunotherapy or targeted therapy was an important treatment method for patients with advanced cancers. There were no studies evaluating the predictive effect of ALBI grade in CCA these patients. Exploring the prognostic value of ALBI grade in patients treated with immunotherapy or targeted therapies would more fully evaluate and confirm the value of ALBI grade. Subgroup analysis also suggested that ALBI grade may be more applicable for Asian populations as a prognostic indicator. Three studies found that ALBI grade was not associated with the prognosis of CCA patients [15,16,24]. Our study concluded that high ALBI grade predicted unfavorable survival through the comprehensive analysis.

Many scoring systems were used to assess liver function, such as Child Turcotte-Pugh (CTP) classification system and the model for end-stage liver disease (MELD) score [27,28]. The ALBI grading system proposed by Johnson et al, was a tool specifically used to assess liver function. Unlike the other scoring systems, the ALBI grade model only took into account two laboratory parameters: albumin and bilirubin. Many studies explored the prognostic stratification ability of ALBI grade in tumors. Imamura et al showed that OS was significantly worse in the ALBI high group than those in the ALBI low group in pancreatic cancer [29]. Koh et al found that the ALBI grade was a significant prognostic factor in patients with colorectal cancer [30]. Luo et al proved that the preoperative ALBI grade was important in the prognosis of gastric cancer patients after gastrectomy [31]. The prognostic value of ALBI grade also was demonstrated in lung cancer, urothelial carcinoma and esophageal squamous cell carcinoma [32–34]. Moreover, a meta-analysis revealed that ALBI grade had good diagnostic value in predicting liver failure after hepatectomy in HCC patients [35]. Two other meta-analyses demonstrated the predictive value of ALBI grade in patients with HCC [36,37]. Combined with these findings, we have reasons to believe the reliability of ALBI grade’s predictive power in CCA patients.

We tried to explain from the composition of ALBI grade that why high ALBI grade predicted poor prognosis in CCA patients. Bilirubin is synthesized and secreted by liver cells. High serum bilirubin levels suggest biliary obstruction and liver dysfunction. In colorectal cancer patients receiving chemotherapy, it has been observed that patients with high serum bilirubin levels respond more poorly to chemotherapy treatment [38]. Accumulating evidences have shown that high bilirubin level can promote multiple tumor progression and is associated with poor prognosis [39,40]. Albumin is produced by the liver, and low albumin levels in the blood can be a sign of malnutrition and dysfunction in liver synthesis. Hypoalbuminemia can lead to the dysfunction of multiple immune cells [41,42]. In addition, serum albumin can directly inhibit the proliferation of tumor cells [43]. Cumulative studies have shown that hypoalbuminemia is associated with poor survival outcomes in different tumors [44–46]. High ABLI grade consisted of high bilirubin and low albumin levels represent significant poor liver functions, malnutrition and immunosuppression. These arguments may efficiently support that high ABLI grade can be utilized as a prognostic indicator for CCA patients.

There were some limitations in the study. Firstly, all articles included in this meta-analysis were retrospective studies with inherent limitations. Secondly, most of the included studies were from China. Therefore, more large-scale studies from different regions were warranted to further assess the prognostic value of ALBI grade for CCA patients. Thirdly, the HR for the different populations was discrepant. Fourthly, due to data limitations, we did not compare ALBI grade with other liver function evaluation systems. Finally, due to the lack of data, we were unable to assess the relationship between ABLI grade and some pathological features. More specialized studies were needed to evaluate the relationship between ALBI grade and pathological features.

In conclusion, high ALBI grade predicted adverse survival outcomes in CCA patients for Asian population. ALBI grade may be an effective prognostic indicator for CCA patients. Because of its simplicity and ease of calculation, clinicians can use it to quickly stratify CCA patients to formulate personalized treatment strategies. The combination of high ALBI grade and other prognostic markers may better predict the prognosis of patients with CCA. Future investigations should explore the potential value of the ALBI grade in predicting outcomes for patients receiving immunotherapy or targeted therapy, particularly in diverse patient populations.

## Supporting Information

S1 Checklist PRISMA_2020_checklist. (DOCX)

S1 Table Specific information of 1063 articles. (DOCX)
