# Supplementary material for: Association of albumin-bilirubin grade with survival outcomes in patients with cholangiocarcinoma
Source: PLoS One. 2025 May 7;20(5):e0321758. doi: 10.1371/journal.pone.0321758 (PMC12058172; doi:10.1371/journal.pone.0321758)
Supplement: S1 Table — (DOCX) [file pone.0321758.s002.docx]

| Title | Year | Author |
| --- | --- | --- |
| A case of cryptogenic pseudocirrhosis causing acute liver failure: When clinic and radiology work together | 2019 | M. Finocchi; O. Para; G. Zaccagnini; L. Corbo; L. Maddaluni; M. Giampieri; E. Blasi; M. Ronchetti; C. Nozzoli |
| A case of cutaneous metastasis of cholangiocarcinoma | 2003 | S. H. Kim; S. J. Youn; J. H. Park; W. S. Kim; D. Y. Lee; E. S. Lee |
| A case of gallbladder neuroendocrine carcinoma diagnosed preoperatively using somatostatin receptor scintigraphy | 2020 | Y. Kamikihara; S. Tanoue; M. Kawahira; H. Iwaya; S. Arima; F. Sasaki; Y. Nasu; S. Hashimoto; S. Kanmura; M. Higashi; K. Gejima; A. Ido |
| A case of mucinous cholangiocarcinoma showing features of hepatocellular carcinoma | 2013 | K. Kai; Y. Ide; A. Miyoshi; M. Masuda; T. Ide; K. Kitahara; H. Irie; J. Nakajima; H. Noshiro; O. Tokunaga |
| A case of persistent JP drain output and ileus after cholecystectomy | 2015 | V. Mittal; R. Sao; L. A. Gollapudi; D. Jodorkovsky |
| A case of profound jaundice with multiple contributory factors | 2018 | S. K. Sharma; K. Das; V. V. Mittal; P. Kar |
| A Case of Secondary Sclerosing Cholangitis in the Setting of Non-Hodgkin's Lymphoma | 2016 | S. Umar; M. Sial; A. Christou; A. Kulkarni |
| A Case of Type II Mirizzi Syndrome | 1990 | K. H. Jin; L. J. Hyeong; S. M. Jun; K. Koing-Bo; C. J. A. E. Chun; C. M. Kwan |
| A case of unresectable combined hepatocellular and cholangiocarcinoma treated with atezolizumab plus bevacizumab | 2022 | N. Saito; T. Hatanaka; S. Nakano; Y. Hazama; S. Yoshida; Y. Hachisu; Y. Tanaka; T. Yoshinaga; K. Kashiwabara; N. Kubo; Y. Hosouchi; H. Tojima; S. Kakizaki; T. Uraoka |
| A case of unsuccessful treatment of heparin-induced thrombocytopenia (HIT) with fondaparinux | 2012 | A. C. Miranda; J. L. Donovan; M. T. Tran; J. M. Gore |
| A case with life-threatening secondary sclerosing cholangitis caused by nivolumab | 2021 | Y. Yoshikawa; M. Imamura; K. Yamaoka; Y. Kosaka; E. Murakami; K. Morio; H. Fujino; T. Nakahara; W. Okamoto; M. Yamauchi; T. Kawaoka; M. Tsuge; A. Hiramatsu; C. N. Hayes; H. Aikata; K. Fujitaka; K. Arihiro; N. Hattori; K. Chayama |
| A Clinical Outcomes Analysis for Resectable Intrahepatic Cholangiocarcinoma in an Urban Academic Medical Center | 2019 | G. Patel; P. Brodin; N. Ohri; A. Kaubisch; S. Bellemare; M. Kinkhabwala; S. Kalnicki; M. K. Garg; C. Guha; R. Kabarriti |
| A comparative study of efficacy and safety of transarterial chemoembolization with CalliSpheres and conventional transarterial chemoembolization in treating unresectable intrahepatic cholangiocarcinoma patients | 2022 | T. Sun; W. Zhang; L. Chen; Y. Ren; Y. Liu; C. Zheng |
| A comparative study of laparoscopic precise hepatectomy with conventional open hepatectomy in the treatment of primary hepatocellular cancer | 2019 | F. Luo; J. Zhou; W. Yan |
| A component could not be larger than the whole, polyclonal immunoglobulin interference in conjugated/direct bilirubin assay and elimination: A case report | 2022 | N. Soleimani; S. Khaleghpanah; S. Mohammadzadeh |
| A curious case of an unimaginable mimicker for gall bladder cancer | 2018 | N. Dash; V. L. Nag |
| A descriptive study of the histopathologic and biochemical liver test abnormalities in dogs with liver disease in Thailand | 2020 | S. N. Assawarachan; P. Maneesaay; N. Thengchaisri |
| A diagnostic dilemma: A case of cholestatic jaundice due to Al-amyloidosis | 2016 | R. Al-Dabbagh; S. Bharadwaj; S. Patterson; M. Puglia |
| A first-in-human phase I study of sEphB4-HSA (sEphB4) with expansion in hepatocellular (HCC) and cholangiocarcinoma (CCA) | 2018 | J. S. Thomas; H. J. Lenz; S. Iqbal; D. D. Tsao-Wei; A. Barzi; V. Duddalwar; S. Cole; R. Rehman; X. Menendez; V. Krasnoperov; R. Liu; Q. Feng; P. S. Gill; A. B. El-Khoueiry |
| A locally advanced pancreatic body cancer presenting common bile duct invasion resected via distal pancreatectomy after gemcitabine plus nab-paclitaxel chemotherapy: A case report | 2022 | H. Kawasaki; M. Hoshikawa; Y. Kyoden; T. Iijima; H. Kojima; J. Yamamoto |
| A lymphoma case without any finding of physical examination except jaundice | 2012 | O. Zengi; S. Zengi; O. Yiʇit |
| A Machine-Based Approach to Preoperatively Identify Patients with the Most and Least Benefit Associated with Resection for Intrahepatic Cholangiocarcinoma: An International Multi-institutional Analysis of 1146 Patients | 2020 | D. I. Tsilimigras; R. Mehta; D. Moris; K. Sahara; F. Bagante; A. Z. Paredes; A. Moro; A. Guglielmi; L. Aldrighetti; M. Weiss; T. W. Bauer; S. Alexandrescu; G. A. Poultsides; S. K. Maithel; H. P. Marques; G. Martel; C. Pulitano; F. Shen; O. Soubrane; B. G. Koerkamp; I. Endo; T. M. Pawlik |
| A Massive Liver Lesion: A Case of Combined Hepatocellular Cholangiocarcinoma | 2017 | B. Singh; A. Panchal; S. Ahmad |
| A Matched Case-Control Study of Preoperative Biliary Drainage in Patients with Pancreatic Adenocarcinoma: Routine Drainage Is Not Justified | 2009 | J. J. Mezhir; M. F. Brennan; R. E. Baser; M. I. D'Angelica; Y. Fong; R. P. DeMatteo; W. R. Jarnagin; P. J. Allen |
| A molecular adsorbent recycling system in treating posthepatectomy acute hepatic failure patients with hepatocellular carcinoma: A bridge to liver transplantation | 2006 | Y. Wang; Y. Liu; W. Zheng; Y. Ming; Z. Shen |
| A more favourable early survival is observed following resection of pancreatic ductal adenocarcinoma if clinical jaundice is resolved preoperatively | 2009 | K. Dajani; R. A. Smith; S. Dodd; P. Whelan; M. Raraty; R. Sutton; F. Campbell; J. P. Neoptolemos; P. Ghaneh |
| A multicenter phase II study of S-1 for gemcitabine-refractory biliary tract cancer | 2013 | E. Suzuki; M. Ikeda; T. Okusaka; S. Nakamori; S. Ohkawa; T. Nagakawa; N. Boku; H. Yanagimoto; T. Sato; J. Furuse |
| A newly improved POSSUM scoring system for prediction of morbidity in patients with pancreaticoduodenectomy | 2020 | Z. L. Zhang; L. Chen; L. Peng; S. C. Li; P. Guo; M. Zhang |
| A nomogram based on pretreatment clinical parameters for the prediction of inadequate biochemical response in primary biliary cholangitis | 2020 | S. Tian; Y. Liu; K. Sun; X. Zhou; S. Ma; M. Zhang; X. Zhou; L. Wang; Y. Han |
| A Nomogram to Predict Hypertrophy of Liver Segments 2 and 3 After Right Portal Vein Embolization | 2016 | Y. Mise; G. Passot; X. Wang; H.-C. Chen; S. Wei; K. W. Brudvik; T. A. Aloia; C. Conrad; S. Y. Huang; J.-N. Vauthey |
| A Novel Classification of Intrahepatic Cholangiocarcinoma Phenotypes Using Machine Learning Techniques: An International Multi-Institutional Analysis | 2020 | D. I. Tsilimigras; J. M. Hyer; A. Z. Paredes; A. Diaz; D. Moris; A. Guglielmi; L. Aldrighetti; M. Weiss; T. W. Bauer; S. Alexandrescu; G. A. Poultsides; S. K. Maithel; H. P. Marques; G. Martel; C. Pulitano; F. Shen; O. Soubrane; B. G. Koerkamp; I. Endo; T. M. Pawlik |
| A Novel Nomogram Based on Hepatic and Coagulation Function for Evaluating Outcomes of Intrahepatic Cholangiocarcinoma After Curative Hepatectomy: A Multi-Center Study of 653 Patients | 2021 | Y. Cai; B. Zhang; J. Li; H. Li; H. Liu; K. Xie; C. Du; H. Wu |
| A novel noninvasive method for predicting liver fibrosis by quantifying the estrangement of indocyanine green retention rate and tc-99m-diethylenetriamine-penta-acetic acid-galactosyl human serum albumin scintigraphy | 2020 | T. Hanaki; E. Uchinaka; T. Yagyu; M. Morimoto; J. Watanabe; K. Miyatani; K. Kihara; T. Matsunaga; M. Yamamoto; Y. Fukumoto; N. Tokuyasu; S. Takano; T. Sakamoto; S. Honjo; T. Hasegawa; Y. Fujiwara |
| A Novel Prognostic Model for Primary Sclerosing Cholangitis | 2015 | E. M. de Vries; J. Wang; K. D. Williamson; M. M. Leeflang; K. Boonstra; U. Beuers; R. W. Chapman; R. Geskus; C. Y. Ponsioen |
| A Novel Prognostic Nomogram for Patients With Recurrence of Intrahepatic Cholangiocarcinoma After Initial Surgery | 2020 | K.-L. Xing; L.-H. Lu; X. Huang; C.-B. He; Y.-D. Song; R.-P. Guo; S.-P. Li |
| A Novel Prognostic Scoring System of Intrahepatic Cholangiocarcinoma With Machine Learning Basing on Real-World Data | 2020 | Z. Li; L. Yuan; C. Zhang; J. Sun; Z. Wang; Y. Wang; X. Hao; F. Gao; X. Jiang |
| A novel technique for central hepatectomy: Maintain the blood supply and biliary drainage on one side and the blood supply from the portal vein on the other | 2013 | C. Tang; J. T. Yang; H. X. Chen; X. C. Liang; H. M. Liu; P. Chen |
| A novel technique of inserting pancreaticogastrostomy with duct-to-mucosa anastomosis can potentially reduce postoperative pancreatic fistula | 2017 | C. D. Lu; J. Huang; Y. F. Hua; A. A. Javed; J. He; S. D. Wu; W. M. Yu; C. J. Lu |
| A pancreaticoduodenectomy risk model derived from 8575 cases from a national single-race population (japanese) using a web-based data entry system: The 30-day and in-hospital mortality rates for pancreaticoduodenectomy | 2014 | W. Kimura; H. Miyata; M. Gotoh; I. Hirai; A. Kenjo; Y. Kitagawa; M. Shimada; H. Baba; N. Tomita; T. Nakagoe; K. Sugihara; M. Mori |
| A Patient with Primary Sclerosing Cholangitis and Diffuse Biliary Dilation | 2021 | M. P. Fejleh; S. E. Yang; S. Kim |
| A pdcd1 role in the genetic predisposition to nafld-hcc? | 2021 | N. Eldafashi; R. Darlay; R. Shukla; M. V. McCain; R. Watson; Y. L. Liu; N. McStraw; M. Fathy; M. A. Fawzy; M. Y. W. Zaki; A. K. Daly; J. P. Maurício; A. D. Burt; B. Haugk; H. J. Cordell; C. Bianco; J. F. Dufour; L. Valenti; Q. M. Anstee; H. L. Reeves |
| A phase I delayed-start, randomized and pharmacodynamic study of metformin and chemotherapy in patients with solid tumors | 2019 | M. W. Saif; S. Rajagopal; J. Caplain; E. Grimm; O. Serebrennikova; M. Das; P. N. Tsichlis; R. Martell |
| A phase i study investigating the safety and pharmacokinetics of highly bioavailable curcumin (Theracurmin®) in cancer patients | 2013 | M. Kanai; Y. Otsuka; K. Otsuka; M. Sato; T. Nishimura; Y. Mori; M. Kawaguchi; E. Hatano; Y. Kodama; S. Matsumoto; Y. Murakami; A. Imaizumi; T. Chiba; J. Nishihira; H. Shibata |
| A Phase I/II Study of Biweekly Carboplatin and Nab-paclitaxel With Concurrent Radiotherapy for Patients With Locally Advanced Unresectable Stage III Non–small-cell Lung Cancer | 2021 | H. Tanaka; Y. Hasegawa; T. Makiguchi; F. Okumura; C. Tabe; T. Shiratori; Y. Ishioka; M. Itoga; K. Taima; J. Yokouchi; Y. Hatayama; M. Aoki; S. Tasaka |
| A phase Ib study of guadecitabine and durvalumab in patients with advanced hepatocellular carcinoma, pancreatic adenocarcinoma, and biliary cancers | 2022 | S. Algaze; D. L. Hanna; N. S. Azad; J. S. Thomas; S. Iqbal; D. Habib; Y. Ning; A. Barzi; R. Patel; H. J. Lenz; A. B. ElKhoueiry |
| A pooled analysis of transarterial radioembolization with yttrium-90 microspheres for the treatment of unresectable intrahepatic cholangiocarcinoma | 2019 | Y. Zhen; B. Liu; Z. Chang; H. Ren; Z. Liu; J. Zheng |
| A prediction model of major complications after radiofrequency ablation for recurrent hepatocellular carcinoma patients | 2021 | C. Xu; H. Zheng; T. Shi; J. Qi; X. Zhao; J. Li; Y. Feng; Q. Zhu |
| A predictive factor of insufficient liver regeneration after preoperative portal vein embolization | 2014 | K. Mihara; T. Sugiura; Y. Okamura; H. Kanemoto; T. Mizuno; M. Moriguchi; T. Aramaki; K. Uesaka |
| A prognostic index to identify patients with intrahepatic cholangiocarcinoma who could benefit from gemcitabine plus cisplatin | 2016 | S. Y. Lee; H. S. Kim; Y. J. Choi; K. H. Park; S. W. Shin; Y. H. Kim; S. T. Kim |
| A prognostic scoring system based on clinical features of intrahepatic cholangiocarcinoma: The Fudan score | 2011 | W. Jiang; Z. C. Zeng; Z. Y. Tang; J. Fan; H. C. Sun; J. Zhou; M. S. Zeng; B. H. Zhang; Y. Ji; Y. X. Chen |
| A prospective pilot study of combined intra-operative radiotherapy for centrally located hepatocellular carcinomas | 2017 | Y. H. Liu; L. M. Wang; J. X. Wu; W. Q. Rong; F. Wu; M. H. Li; Y. Zhang; S. T. Lin; Y. L. Zheng; Q. F. Feng |
| A RANDOMIZED TRIAL OF INTRAHEPATIC INFUSION OF FLUORODEOXYURIDINE WITH DEXAMETHASONE VERSUS FLUORODEOXYURIDINE ALONE IN THE TREATMENT OF METASTATIC COLORECTAL-CANCER | 1992 | N. Kemeny; K. Seiter; D. Niedzwiecki; D. Chapman; E. Sigurdson; A. Cohen; J. Botet; P. Oderman; P. Murray |
| A rare case of adenosquamous variant of intrahepatic cholangiocarcinoma | 2020 | A. R. Hudgi; M. Zandu; T. Le; A. N. Krutchik |
| A Rare Case of Isolated Hepatic Sarcoidosis With Cholestatic Jaundice and Review of Literature | 2014 | P. Devaraj; H. Rathinamanickam; S. Gayam |
| A Rare Case of Metastatic Hepatocellular Carcinoma to the Hard Palate | 2022 | P. R. P. Lyon; S. Trevathan; R. Talukdar; D. D. Nguyen; C. M. McPhaul; D. Rampisela |
| A rare case of oriental cholangiohepatitis | 2015 | N. S. Addepally; J. S. Klair; A. Kaur; A. Agarwal; F. Aduli |
| A Rare Case of Peliosis Hepatis in a Young Female with End-Stage Renal Disease on Chronic Hemodialysis | 2014 | V. R. Garlapati; A. Gupta; H. Hertan |
| A rare case of primary biliary cholangitis (PBC) and hereditary hemorrhagic telangiectasia (HHT) co-existing in an Asian male patient | 2018 | J. Kuang; K. Kaliyaperumal |
| A rare case with fever, jaundice and hepato-renal dysfunction | 2017 | K. Kurokohchi; H. Nagami; S. Yamagata; S. Honda; Y. Ishibashi |
| A rare cause of left back pain | 2014 | C.-H. Lo; H.-C. Chu |
| A rare cause of non-obstructive jaundice - Stauffer's syndrome - A case report | 2019 | C. Marius; N. C. Ciorba; E. Becica; S. Sandor; A. Pruna; R. Szilagyi; M. Greavu; S. Bataga |
| A rare genetic variant in the manganese transporter SLC30A10 and elevated liver enzymes in the general population | 2022 | A. S. Seidelin; B. G. Nordestgaard; A. Tybjærg-Hansen; H. Yaghootkar; S. Stender |
| A rare presentation of multiple myeloma: A case report of hepatic amyloidosis | 2019 | A. Sadeghi; M. Nejati; A. Moghaddas |
| A rare synchrony of adenocarcinoma of the ampulla with an ileal gastrointestinal stromal tumor: A case report | 2022 | V. V. K. Matli; G. B. Zibari; G. Wellman; P. Ramadas; S. Pandit; J. Morris |
| A Retrospective Study of Lenvatinib Monotherapy or Combined With Programmed Cell Death Protein 1 Antibody in the Treatment of Patients With Hepatocellular Carcinoma or Intrahepatic Cholangiocarcinoma in China | 2021 | S. Zhu; C. Liu; Y. Dong; J. Shao; B. Liu; J. Shen |
| A review of factors predicting perioperative death and early outcome in hepatopancreaticobiliary cancer surgery | 2010 | C. D. Mann; T. Palser; C. D. Briggs; I. Cameron; M. Rees; J. Buckles; D. P. Berry |
| A review of the risk stratification models used in the management of oncological hepato-pancreato-biliary surgical patients | 2021 | A. Coombs; C. Jordan; S. Hussain; O. Ghandour |
| A revised natural history model for primary sclerosing cholangitis | 2000 | W. R. Kim; T. M. Therneau; R. H. Wiesner; J. J. Poterucha; J. T. Benson; M. Malinchoc; N. F. LaRusso; K. D. Lindor; E. R. Dickson |
| A Severe Case of Drug-Induced Liver Injury after Gemcitabine Administration: A Highly Probable Causality Grading as Assessed by the Updated RUCAM Diagnostic Scoring System | 2020 | I. Mascherona; C. Maggioli; M. Biggiogero; O. Mora; L. Marelli |
| A simple scoring system to estimate perioperative mortality following liver resection for primary liver malignancy-the Hepatectomy Risk Score (HeRS) | 2021 | D. Moris; B. I. Shaw; C. Ong; A. Connor; M. L. Samoylova; S. J. Kesseli; N. Abraham; J. Gloria; R. Schmitz; Z. W. Fitch; B. M. Clary; A. S. Barbas |
| A Study on correlation between ICG regression test and 99mTc-GSA scintigraphy in liver function evaluation before hepatectomy for perihilar cholangiocarcinoma | 2018 | A. Inoue; T. Noji; Y. Nakanishi; T. Asano; T. Nakamura; T. Tsuchikawa; K. Okamura; T. Shichinohe; S. Hirano |
| A study on predicting cases that would benefit from proton beam therapy in primary liver tumors of less than or equal to 5 cm based on the estimated incidence of hepatic toxicity | 2022 | Y. Uchinami; N. Katoh; R. Suzuki; T. Kanehira; M. Tamura; S. Takao; T. Matsuura; N. Miyamoto; Y. Fujita; F. Koizumi; H. Taguchi; K. Yasuda; K. Nishioka; I. Yokota; K. Kobashi; H. Aoyama |
| A study on risk factors and diagnostic efficiency of posthepatectomy liver failure in the nonobstructive jaundice | 2018 | H. Wang; S.-C. Lu; L. He; J.-H. Dong |
| A Surgically Resected Pancreatic Schwannoma with Obstructive Jaundice with Special Reference to Differential Diagnosis from Other Cystic Lesions in the Pancreas | 2018 | T. Watanabe; K. Araki; N. Ishii; T. Igarashi; A. Watanabe; N. Kubo; H. Kuwano; K. Shirabe |
| A Transient Treatable Tumor of the Pancreas | 2022 | V. Boppana; S. Paleti; C. Ling; N. Volpicelli; D. McCarthy |
| A unique presentation of obstructive jaundice, severe cholangitis and ascites in a post chemoradiation patient | 2022 | S. D. Mohapatra; N. Premaletha; S. S. Sressh; J. John; A. Chakravorty; A. Verma; A. S. J. Raja |
| A young man from Peru with fever and abdominal pain | 2005 | B. M. Clark; B. A. Lloyd; G. W. Christopher; W. F. Foody |
| Ability of Biochemical Parameters to Distinguish between Bile Duct Cancer and Gall Bladder Stones - A Case Control Study in a Tertiary Care Hospital of Pokhara Valley | 2013 | S. K. Yadav; A. Mittal; K. Sapkota; S. P. Gupta; B. Sathian |
| Ablation approach for primary liver tumors: Peri-operative outcomes | 2018 | N. G. Berger; J. L. Herren; C. Liu; R. H. Burrow; J. P. Silva; S. Tsai; K. K. Christians; T. C. Gamblin |
| Abnormal aminotransferases, think beyond the liver | 2019 | S. Ghazaleh; Y. Khader; M. Ghanim; P. Shastri; T. Alhmoud |
| Abnormal liver enzymes secondary to congenital hepatic fibrosis with multiple biliary cysts | 2021 | M. Tseng; T. Syed; R. Sterling |
| Abnormal liver tests are not sufficient for diagnosis of hepatic graft-versus-host disease in critically ill patients | 2022 | A. H. Yang; M. A. T. Han; N. Samala; B. S. Rizvi; R. Marchalik; O. Etzion; E. C. Wright; R. Patel; V. Khan; D. Kapuria; V. Samala Venkat; D. E. Kleiner; C. Koh; J. A. Kanakry; C. G. Kanakry; S. Pavletic; K. M. Williams; T. Heller |
| Abstract No. 160 Unresectable intrahepatic cholangiocarcinoma treated with radiation segmentectomy/lobectomy using Y90-labeled glass microspheres | 2021 | P. Kumar; R. Mhaskar; A. Bibok; R. Kim; D. Anaya; J. Frakes; S. Hoffe; G. El-Haddad; J. Choi; B. Kis |
| Abstract No. 32 Multi-institutional review of patients receiving Y-90 transarterial radioembolization (TARE) with hepatic tumors status post partial hepatectomy | 2022 | A. Rohr; Z. Collins; A. Hodson; K. Zhang; H. Krebs; R. Ghandi; R. O'Hara; N. Akhter; E. Wang; C. Grilli; J. Brower; S. Peck; M. Petroziello; A. A. Aal; J. Golzarian; D. Brown |
| Abstract No. 560 Safety and efficacy of radioembolization for intrahepatic cholangiocarcinoma with ≥150 Gy MIRD: a single-center review | 2020 | J. Core; C. Padula; M. Elboraey; Z. Devcic; C. Ritchie; A. Lewis; J. McKinney; R. Paz-Fumagalli; G. Frey; B. Toskich |
| Abstract No. 561 Risk factors of new onset or worsening ascites after Y-90 radioembolization of primary liver tumors | 2022 | J. Marlow; F. Jabboure; N. Mani; N. Rostambeigi |
| Abstract No. 8 Yttrium-90 radioembolization in unresectable intrahepatic cholangiocarcinoma: a 17-year single-institution study | 2021 | A. Gupta; A. Gordon; A. Gabr; A. Riaz; K. Sato; B. Thornburg; K. Desai; R. Salem; R. Lewandowski |
| Acetaminophen Toxicity, the Red Herring of Lymphoma | 2017 | A. Gampa; J. Amin; A. Singh |
| Acute esophageal necrosis: Patient with a history of liver cancer on capecitabine | 2021 | Y. E. Muzahim; M. D. Patel; R. M. Coman; A. Ahmed |
| Acute kidney injury due to high-output external biliary drainage in a patient with malignant obstructive jaundice: A case report | 2019 | U. Jayarajah; O. Basnayake; P. K. Wijerathne; S. Sivaganesh |
| Acute liver injury in the context of immune checkpoint inhibitor-related colitis treated with infliximab | 2019 | H. C. Zhang; W. Luo; Y. Wang |
| Acute myeloid leukaemia: An unusual cause of biliary strictures | 2019 | A. Beck; H. Hunter; S. Jackson; D. Sheridan |
| Addition of molecular adsorbent recirculating system (MARS®) albumin dialysis for the preoperative management of jaundiced patients with hilar cholangiocarcinoma | 2013 | J. M. Regimbeau; D. Fuks; E. Chapuis-Roux; T. Yzet; C. Cosse; E. Bartoli; E. N'Guyen-Khac; B. Robert; E. Lobjoie |
| Adenocarcinoma of Ampulla Vater with Metastatic Process Manifest as Jaundice and Biliary Sepsis: A Rare Case Report | 2022 | A. Pramudita; H. Setiawan; S. Immanuel; K. Jaya; D. Alexander; L. Rianto |
| ADEQUATE BIOCHEMICAL RESPONSE TO URSODEOXYCHOLIC ACID IN PATIENTS WITH PRIMARY BILIARY CHOLANGITIS: IS NORMALIZATION OF SERUM LIVER TESTS IMPORTANT? | 2022 | C. Corpechot; S. Lemoinne; B. E. Hansen; G. Hirschfield; A. Gulamhusein; A. J. Montano-Loza; E. Lytvyak; C. Schramm; A. Pares; I. Olivas; J. E. Eaton; K. Osman; G. N. Dalekos; N. K. Gatselis; F. Nevens; N. Cazzagon; A. Zago; F. P. Russo; N. Abbas; P. Trivedi; D. Thorburn; F. Saffioti; L. Barkai; D. Roccarina; V. Calvaruso; A. Fichera; E. Medina-Morales; A. Bonder; V. Patwardhan; A. G. Villamil; N. Sobenko; C. Rigamonti; M. Carbone; P. Invernizzi; L. Cristoferi; A. Van Der Meer; R. De Veer; E. Zigmond; E. Yehezkel; A. E. Kremer; A. Deibel; T. Bruns; K. Große; A. Wetten; J. Dyson; D. Jones; J. Dumortier; G. P. Pageaux; V. De Ledinghen; O. Chazouilleres; F. Carrat |
| Adjuvant radiotherapy and chemotherapy offer a recurrence and survival benefit in patients with resected perihilar cholangiocarcinoma | 2021 | J. H. Im; G. H. Choi; W. J. Lee; D. H. Han; S. W. Park; S. Bang; H. J. Choi; J. Seong |
| ADULT DIAGNOSED PRIMARY SCLEROSING CHOLANGITIS (PSC) SUBJECTS HAVE WORSE INTRAHEPTIC BILIARY DISEASE ON MAGNETIC RESONANCE IMAGING AT DIAGNOSIS COMPARED TO PEDIATRIC PSC SUBJECTS | 2021 | C. Jarasvaraparn; L. Chi; S. Bhimaniya; J. M. Stoll; S. Kulkarni |
| Advanced Combined Hepatocellular Carcinoma and Cholangiocarcinoma (cHCC-CC) in a Patient With Alcoholism and Chronic Hepatitis C | 2015 | Y. Gao; T.-P. Lee; C. Fan |
| Advances in liver transplantation | 2005 | T. T. Tran; N. Nissen; F. F. Poordad; P. Martin |
| Advantages of laparoscopic left hemihepatectomy: A meta-analysis | 2019 | X. Yin; D. Luo; Y. Huang; M. Huang; S. Abd-Elsalam |
| Age-adjusted Charlson Comorbidity Index predicts survival in intrahepatic cholangiocarcinoma patients after curative resection | 2020 | W. F. Qu; P. Y. Zhou; W. R. Liu; M. X. Tian; L. Jin; X. F. Jiang; H. Wang; C. Y. Tao; Y. Fang; Y. F. Zhou; S. S. Song; Z. B. Ding; Y. F. Peng; Z. Dai; S. J. Qiu; J. Zhou; J. Fan; Z. Tang; Y. H. Shi |
| Agenesis of dorsal pancreas associated with periampullary pancreaticobiliary type adenocarcinoma | 2014 | R. M. Sannappa; J. Buragohain; D. Sarma; U. K. Saikia; B. K. Choudhury |
| Agenesis of gallbladder presenting with obstructive jaundice | 2016 | A. Al-Khazraji; H. Alkhawam; B. Garrido |
| Aids cholangiopathy due to mycobacterium avium complex: A case report | 2016 | A. Urbina; J. E. L. Yap; G. Vettiankal; B. M. Attar; J. H. Stroger |
| Alagille syndrome: An uncommon cause of intrahepatic cholestasis in adults | 2019 | W. Zhang; X. Zhao; J. Huang; X. Ou; J. Jia |
| Albumin-Bilirubin Grade and Tumor Burden Score Predict Outcomes Among Patients with Intrahepatic Cholangiocarcinoma After Hepatic Resection: a Multi-Institutional Analysis | 2023 | M. M. Munir; Y. Endo; H. A. Lima; L. Alaimo; Z. Moazzam; C. Shaikh; G. A. Poultsides; A. Guglielmi; L. Aldrighetti; M. Weiss; T. W. Bauer; S. Alexandrescu; M. Kitago; S. K. Maithel; H. P. Marques; G. Martel; C. Pulitano; F. Shen; F. Cauchy; B. G. Koerkamp; I. Endo; T. M. Pawlik |
| Albumin-Bilirubin Grade as a Novel Predictor of Survival in Advanced Extrahepatic Cholangiocarcinoma | 2018 | Y. Wang; Q. Pang; H. Jin; L. Zhou; X. Hu; Z. Qian; Z. Man; S. Yang; H. Liu |
| Albumin-bilirubin grade as a prognostic indicator for patients with non-hepatocellular primary and metastatic liver malignancy undergoing Yttrium-90 radioembolization using resin microspheres | 2020 | A. Azar; Z. Devcic; R. Paz-Fumagalli; L. L. C. Vidal; J. M. McKinney; G. Frey; A. R. Lewis; C. Ritchie; J. S. Starr; K. Mody; B. Toskich |
| Albumin-to-Alkaline Phosphatase Ratio is an Independent Prognostic Indicator in Combined Hepatocellular and Cholangiocarcinoma | 2020 | F. Zhang; S. Lu; M. Tian; K. Hu; R. Chen; B. Zhang; Z. Ren; Y. Shi; X. Yin |
| Alkaline Phosphatase at the Start of Liver Directed Radiation Is A Predictor of Long Term Liver Volume Changes | 2017 | K. A. Kovtun; N. Giacolone; S. Patel; J. Y. Wo; J. A. Wolfgang; T. S. Hong |
| Alkaline phosphatase normalization is associated with better prognosis in primary sclerosing cholangitis | 2011 | P. P. Stanich; E. Björnsson; A. A. Gossard; F. Enders; R. Jorgensen; K. D. Lindor |
| Alterations in hepatic lobar function in regenerating rat liver | 2015 | A. Fülöp; A. Budai; Z. Czigány; G. Lotz; K. Dezso; S. Paku; L. Harsányi; A. Szijártó |
| Alveolar echinococcosis of liver: A diagnostic problem in a nonendemic area | 2015 | K. S. Madhusudhan; D. N. Srivastava; N. R. Dash; A. Venuthurimilli; R. Sharma; S. Gamanagatti; A. K. Gupta |
| An autopsy case of primary squamous cell carcinoma of the liver | 2012 | K. Dohmen; H. Tanaka; M. Haruno; H. Fujiwara; I. Kobayashi; T. Kiyoshim; S. Shimoda; H. Sakai |
| An Efficient Nomogram for Discriminating Intrahepatic Cholangiocarcinoma From Hepatocellular Carcinoma: A Retrospective Study | 2022 | Y. Q. Si; X. Q. Wang; C. C. Pan; Y. Wang; Z. M. Lu |
| An Exploratory Study of Neoadjuvant Cetuximab Followed by Cetuximab and Chemoradiotherapy in Women with Newly Diagnosed Locally Advanced Cervical Cancer | 2022 | P. M. Fracasso; L. R. Duska; P. H. Thaker; F. Gao; I. Zoberi; F. Dehdashti; B. A. Siegel; L. Uliel; C. O. Menias; P. K. Rehm; S. A. Goodner; A. N. Creekmore; H. L. Lothamer; J. S. Rader |
| An extremely rare case of intrahepatic sarcomatoid cholangiocarcinoma | 2023 | D. Q. Lieu; T. N. Anh; T. T. Hoa; V. T. Phuong; N. M. Duc |
| An Imaging Biomarker for Assessing Hepatic Function in Patients With Primary Sclerosing Cholangitis | 2019 | J. Schulze; H. Lenzen; J. B. Hinrichs; B. Ringe; M. P. Manns; F. Wacker; K. I. Ringe |
| An open-label study to describe pharmacokinetic parameters of erlotinib in patients with advanced solid tumors with adequate and moderately impaired hepatic function | 2012 | C. L. O'Bryant; P. Haluska; L. Rosen; R. K. Ramanathan; B. Venugopal; S. Leong; R. Boinpally; A. Franke; K. Witt; J. Evans; C. Belani; S. Gail Eckhardt; S. Ramalingam |
| AN ORIGINAL RECONSTRUCTIVE METHOD AFTER PYLORUS-PRESERVING PANCREATICODUODENECTOMY | 1993 | G. Debernardinis; A. Agnifili; P. Gola; I. Ibi; F. Gianfelice; G. Carducci; R. Verzaro |
| An Unusual Case of Cholestatic Drug-Induced Liver Injury Following a Single Dose of Pembrolizumab | 2017 | J. R. Penn; V. Rustgi |
| An unusual case of jaundice in secondary diabetes | 2003 | R. M. Reynolds; S. R. Laidlaw; I. W. Campbell; I. Nawroz |
| An Unusual Case of Noninvasive Adenocarcinoma Arising in a Localized Adenomyoma of the Gallbladder and Review of Literature | 2016 | S. Patel; J. Slade; S. Jakate |
| An unusual cause of acute liver failure | 2006 | T. Tran; J. Wattacheril; H. Shelby |
| An unusual presentation of Hodgkin lymphoma | 2020 | C. T. Jagadeesan; S. Biswas Roy; N. Dennison; S. Roy; J. Raj |
| Analysis of donor risk factors associated with graft outcomes in orthotopic liver transplantation | 2012 | Y. Sirivatanauksorn; V. Taweerutchana; S. Limsrichamrern; P. Kositamongkol; P. Mahawithitwong; S. Asavakarn; C. Tovikkai; V. Sanphasitvong |
| Analysis of surgical treatment for intrahepatic cholangiocarcinoma | 2017 | N. Yasui; M. Inagaki; K. Kitada; N. Tokunaga; H. Iwagaki |
| Analysis of treatment methods and prognostic factors in 354 cases of hilar cholangiocarcinoma: A cohort study | 2020 | Z. Sun; Y. Feng; H. Liu; F. Shen; J. Xiao; X. Kang; Y. Zhao; H. Xiao; W. Zhao; X. Hu; J. Li; H. Ma; G. Cao; N. Su; B. Zhang |
| Anatomical barriers to treatment of malignant liver lesions with yttrium-90 microspheres | 2010 | B. Rathore; J. Novotny; K. Holdeman; Zahiri; S. Paknikar; J. Hankins |
| Anatomical location as a prognostic factor in surgical treatment of gallbladder carcinoma | 2020 | Z. Zhu; K. Luo; B. Wu; H. Liu; Z. Fang; Y. Bai; F. Li; W. Liu; L. Shan |
| Anti-GP2 IgA autoantibodies are associated with liver cirrhosis and severity of the disease in primary sclerosing cholangitis (PSC) | 2017 | G. L. Norman; E. Wunsch; M. Krawczyk; S. Encabo; J. Milo; C. Bentow; M. Mahler; D. Roggenbuck; P. Milkiewicz |
| ANTIPHOSPHOLIPID ANTIBODY SYNDROME in A YOUNG MALE with BILIARY ADENOCARCINOMA | 2021 | H. Ipalawatte; S. Sathian; K. Radicic; S. Mishra |
| Application of intraoperative navigation in 3D laparoscopic pancreaticoduodenectomy: A case report | 2022 | H. Dong; M. Luo; S. Ke; J. Zhan; X. Liu; Z. Li |
| Aspirin is associated with low recurrent risk in hepatitis B virus-related hepatocellular carcinoma patients after curative resection | 2020 | S. H. Young; G. Y. Chau; I. C. Lee; Y. C. Yeh; Y. Chao; T. I. Huo; C. W. Su; H. C. Lin; M. C. Hou; M. H. Lee; Y. H. Huang |
| Assessing resectability in cholangiocarcinoma | 2014 | T. Sano; Y. Shimizu; Y. Senda; T. Kinoshita; Y. Nimura |
| Assessing Textbook Outcomes Following Liver Surgery for Primary Liver Cancer Over a 12-Year Time Period at Major Hepatobiliary Centers | 2020 | D. I. Tsilimigras; K. Sahara; D. Moris; R. Mehta; A. Z. Paredes; F. Ratti; H. P. Marques; O. Soubrane; V. Lam; G. A. Poultsides; I. Popescu; S. Alexandrescu; G. Martel; A. Workneh; A. Guglielmi; T. Hugh; L. Aldrighetti; M. Weiss; T. W. Bauer; S. K. Maithel; C. Pulitano; F. Shen; B. G. Koerkamp; I. Endo; T. M. Pawlik |
| Assessing the Validity of Adult-derived Prognostic Models for Primary Sclerosing Cholangitis Outcomes in Children | 2020 | M. R. Deneau; P. L. Valentino; C. Mack; K. Alqoaer; M. Amin; A. Z. Amir; M. Aumar; M. Auth; A. Broderick; M. DiGuglielmo; L. G. Draijer; W. El-Matary; F. Ferrari; K. N. Furuya; F. Gottrand; N. Gupta; M. Homan; M. K. Jensen; B. M. Kamath; K. M. Kim; K.-L. Kolho; B. Koot; R. Iorio; M. Martinez; T. Miloh; P. Mohan; S. Palle; A. Papadopoulou; A. Ricciuto; L. Saubermann; P. Sathya; E. Shteyer; V. Smolka; A. Tanaka; R. Varier; V. Venkat; B. Vitola; M. Woynarowski; S. Guthery |
| Assessment for liver transplantation in patients with primary sclerosing cholangitis | 1994 | U. Broome; L. S. Eriksson |
| Assessment of blood-products use as predictor of pulmonary complications and surgical-site infection after hepatectomy for hepatocellular carcinoma | 2009 | H. Shiba; Y. Ishii; Y. Ishida; S. Wakiyama; T. Sakamoto; R. Ito; T. Gocho; T. Uwagawa; S. Hirohara; Y. Kita; T. Misawa; K. Yanaga |
| Assessment of functional liver reserve: Old and new in 99mTc-sulfur colloid scintigraphy | 2017 | M. M. Matesan; S. R. Bowen; T. R. Chapman; R. S. Miyaoka; J. W. Velez; M. F. Wanner; M. J. Nyflot; S. Apisarnthanarax; H. J. Vesselle |
| Assessment of Hepatic Arterial Infusion of Floxuridine in Combination with Systemic Gemcitabine and Oxaliplatin in Patients with Unresectable Intrahepatic Cholangiocarcinoma: A Phase 2 Clinical Trial | 2020 | A. Cercek; T. Boerner; B. R. Tan; J. F. Chou; M. Gönen; T. M. Boucher; H. F. Hauser; R. K. G. Do; M. A. Lowery; J. J. Harding; A. M. Varghese; D. Reidy-Lagunes; L. Saltz; N. Schultz; T. P. Kingham; M. I. D'Angelica; R. P. Dematteo; J. A. Drebin; P. J. Allen; V. P. Balachandran; K. H. Lim; F. Sanchez-Vega; N. Vachharajani; M. B. Majella Doyle; R. C. Fields; W. G. Hawkins; S. M. Strasberg; W. C. Chapman; L. A. Diaz; N. E. Kemeny; W. R. Jarnagin |
| Assessment of hepatic function decline after stereotactic body radiation therapy for primary liver cancer | 2017 | D. A. S. Toesca; E. C. Osmundson; R. von Eyben; J. L. Shaffer; A. C. Koong; D. T. Chang |
| Assessment of hepatic function decline after stereotactic body radiation therapy for primary liver tumors using the albumin- bilirubin (ALBI) score | 2016 | D. A. S. Toesca; E. Osmundson; J. Shaffer; R. Von Eyben; A. C. Koong; D. T. Chang |
| Assessment of hepatitis and fibrosis using Gd-EOB-DTPA MRI in dogs | 2020 | T. Tanaka; H. Nishida; K. Mie; H. Yamazaki; L.-S. Lin; H. Akiyoshi |
| Assessment of Liver Function for Evaluation of Long-Term Outcomes of Intrahepatic Cholangiocarcinoma: A Multi-Institutional Analysis of 620 Patients | 2020 | H. Li; J. Li; J. Wang; H. Liu; B. Cai; G. Wang; H. Wu |
| Assessment of the Outcomes of Intrahepatic Cholangiocarcinoma After Ultrasound-Guided Percutaneous Microwave Ablation Based on Albumin-Bilirubin Grade | 2021 | H. Yang; Z. Cheng; Z. Han; F. Liu; X. Yu; J. Yu; P. Liang |
| Associated factors in modulating aflatoxin B1-albumin adduct level in three Chinese populations | 2005 | P. Tao; L. Zhi-Ming; L. Tang-Wei; L. Le-Qun; P. Min-Hao; Q. Xue; Y. Lu-Nam; L. Ren-Xiang; W. Zong-Liang; W. Lian-Wen; W. Qiao; S. Han-Ming; O. Choon-Nam; R. M. Santella |
| Associated factors in modulating aflatoxin B-1-albumin adduct level in three Chinese populations | 2005 | T. Peng; Z. M. Liu; T. W. Liu; L. Q. Li; M. H. Peng; X. Qin; L. N. Yan; R. X. Liang; Z. L. Wei; L. W. Wang; Q. Wang; H. M. Shen; C. N. Ong; R. M. Santella |
| Associating pancreaticostomy and biliaryirrigation for staged pancreaticoduodenectomy approach to pancreatic intraductal papillary mucinous neoplasm with recurrent cholangitis and severe jaundice: A case report | 2016 | C. Dai; S. Lou; F. Zhou |
| Association between promoter hypomethylation and overexpression of autotaxin with outcome parameters in biliary atresia | 2017 | W. Udomsinprasert; N. Kitkumthorn; A. Mutirangura; V. Chongsrisawat; Y. Poovorawan; S. Honsawek |
| Association of serum biomarkers with liver stiffness assessed by transient elastography in 330 children with cholestatic liver disease | 2020 | D. H. Leung; S. Devaraj; N. P. Goodrich; X. Chen; D. B. Rajapakshe; W. Ye; V. Andreev; C. Minard; D. Guffey; J. P. Molleston; L. M. Bass; S. J. Karpen; B. M. Kamath; K. S. Wang; S. S. Sundaram; P. Rosenthal; P. J. McKiernan; K. M. Loomes; M. K. Jensen; S. P. Horslen; J. A. Bezerra; J. C. Magee; R. Merion; R. J. Sokol; B. L. Shneider |
| Association of systemic inflammation index with survival in patients with advanced perihilar cholangiocarcinoma treated with interventional therapy | 2022 | J. Li; L. Gao; T. Liu; D. Feng |
| Autoimmune pancreatitis with normal serum IgG4 levels | 2010 | C. Granato; R. Rong; A. Valente; A. Roy |
| Benefit of second-line chemotherapy for advanced biliary tract cancer | 2017 | F. Moik; J. M. Riedl; T. Winder; A. Bezan; C. Rossmann; J. Szkandera; A. K. Kasparek; R. Schaberl-Moser; M. Pichler; H. Stoger; M. Stotz; A. Gerger; F. Posch |
| Benefits and safety of photodynamic therapy in patients with hilar cholangiocarcinoma: A meta-analysis | 2022 | P. Chen; T. Yang; P. Shi; J. Shen; Q. Feng; J. Su |
| Benign recurrent intrahepatic cholestasis - 2 (BRIC-2)/ABCB11 deficiency in a young child - Report from a tertiary care center in South India | 2021 | K. V. Kalaranjini; J. A. Glaxon; S. Vasudevan; M. L. Arunkumar |
| Bezafibrate for the treatment of primary sclerosing cholangitis | 2010 | S. Mizuno; K. Hirano; M. Tada; K. Yamamoto; Y. Yashima; H. Yagioka; K. Kawakubo; Y. Ito; H. Kogure; T. Sasaki; T. Arizumi; O. Togawa; S. Matsubara; Y. Nakai; N. Sasahira; T. Tsujino; H. Isayama; T. Kawabe; M. Omata; K. Koike |
| Bile acid concentrations in the diagnosis of hepatobiliary disease in the dog | 1985 | S. A. Center; B. H. Baldwin; H. N. Erb; B. C. Tennant |
| Bile duct drainage using a short double-balloon endoscope for a hematoma due to hepatocellular carcinoma after Roux-en-Y reconstruction | 2015 | Y. Kondo; S. Shinozaki; H. Hatanaka; T. Yano; S. Watanabe; K. Tamada; A. K. Lefor; H. Yamamoto |
| Bile is a reliable and valuable source to study cfDNA in biliary tract cancers | 2022 | Z. Li; Y. Liu; J. Fu; J. Mugaanyi; J. Yan; C. Lu; J. Huang |
| Bile Leak Aft er Cholecystectomy | 2014 | S. Mahmood; M. M. Adnan; S. Nusrat; J. Maple |
| Biliary Cyst (aka Choledochal Cyst): A Case Report of an Unusual Cause of End Stage Liver Disease in an 11-year old Female | 2009 | K. Sullivan; T. Kinney; S. J. Schwarzenberg |
| Biliary drainage for obstructive jaundice enhances hepatic energy status in humans: A 31-phosphorus magnetic resonance spectroscopy study | 2002 | D. V. Mann; W. W. M. Lam; N. Magnus Hjelm; N. M. C. So; D. K. W. Yeung; C. Metreweli; W. Y. Lau |
| Biliary drainage improves the predictive value of modified glasgow prognostic scores in inoperable pancreatic cancer | 2016 | C. Iino; T. Shimoyama; T. Igarashi; T. Aihara; K. Ishii; J. Sakamoto; H. Tono; S. Fukuda |
| Biliary indocyanine green excretion as a predictor of hepatic adenosine triphosphate levels in patients with obstructive jaundice | 2000 | K. Chijiiwa; M. Watanabe; K. Nakano; H. Noshiro; M. Tanaka |
| Biliary Rhabdomyoscarcoma Mimicking Choledochal Cyst | 2009 | S. Ali; M. A. Russo; L. Margraf |
| Biopsy diagnosis of inherited liver disease | 2010 | A. Roy; M. J. Finegold |
| Black cohosh induced cholestatic liver injury | 2020 | H. S. Brar; R. Marathi |
| Blood cultures should be collected for acute cholangitis regardless of severity | 2022 | T. Otani; T. Ichiba; K. Seo; H. Naito |
| Brentuximab vedotin in combination with sequential procarbazine, cyclophosphamide and prednisolone for the management of Hodgkin's lymphoma-associated vanishing bile duct syndrome (VBDS) with severe obstructive liver failure | 2019 | M. Fong; S. Boyle; N. Gutta |
| Bridge of preoperative biliary drainage is a useful management for patients undergoing pancreaticoduodenectomy | 2019 | Y. Endo; H. Noda; F. Watanabe; N. Kakizawa; T. Fukui; T. Kato; K. Ichida; H. Aizawa; N. Kasahara; T. Rikiyama |
| Budesonide with Low-Dose 6-Mercaptopurine as a Possible New Treatment for IgG4-Related Sclerosing Cholangitis and Systemic IgG4-Related Disease: A Case Report | 2022 | B. P. M. Gummlich; A. S. A. Hosseini; H. Schwörer |
| Burkitt lymphoma presenting as acute pancreatitis-diagnostic problems with an unusual onset | 2015 | L. Maciejka-Kemblowska; M. Kozlowska; N. Irga-Jaworska; M. Niedzwiecki; M. Szalewska; E. Adamkiewicz-Drozynska |
| Calcified gallbladder cancer: Is it preventable? | 2019 | R. Thakrar; S. Monib; E. Pakdemirli; S. Thomson |
| Can conventional clinical chemistry tests help doctors in the monitoring of oncology patients? | 2021 | V. G. Zaitsev; A. A. Zheltova; S. A. Martynova; E. V. Tibirkova |
| Can immediately treating subcentimeter hepatocellular carcinoma improve the survival of patients? | 2020 | X. Sun; D. Hu; Y. Zhang; N. Lyu; L. Xu; Q. Chen; J. Lai; M. Chen; M. Zhao |
| Can pretreatment hepatic artery perfusion scintigraphy in patients with liver malignancies predict the treatment response of the selective internal radiation therapy with90Y resin microspheres? | 2022 | I. B. Guney; H. T. Balli; K. A. Kucuker; I. Unal; M. Kibar |
| Cannulation procedure optimization for patients with duodenal papillary tumors | 2021 | J. Hong; H. Zhu; W. Zuo; L. Zhu; X. Zhou; X. Zhou; G. Li; Z. Liu; P. Liu; H. Zhen; Y. Zhu; A. Wang; J. Ma; J. Yuan; X. Shu; Y. Zhu; N. Lu; Y. Chen |
| Case report of acute liver failure secondary to hemophagocytic lymphohistiocytosis | 2017 | I. Hader; M. Amin; A. Fisher; S. Tasleem; M. S. Cappell |
| Case Report: Delta bilirubin as an “interference” in direct bilirubin assays | 2015 | A. Chong; S. Saw; S. Sethi |
| Case Report: Trastuzumab Treatment in Adenosquamous Carcinoma of the Extrahepatic Biliary Tract With Her-2 Amplification | 2021 | Y. Hong; X. Li; D. Cao |
| Case series: Thrombus resolution in 2 patients with portal vein thrombosis without anticoagulation-do we need to anticoagulate patients with portal vein thrombosis? | 2013 | A. A. Sule; A. M. Borja; W. Xing; E. Lymen; B. Azucena; T. J. Chin |
| Case Series: Thrombus Resolution in 2 Patients with Portal Vein Thrombosis Without Anticoagulation—Do We Need to Anticoagulate Patients with Portal Vein Thrombosis? | 2013 | A. A. Sule; A. M. Borja; W. Xing; E. Lymen; B. Azucena; T. J. Chin; E. Lymen |
| cDNA microarray profiling of rat cholangiocarcinoma induced by thioacetamide | 2013 | C. N. Yeh; W. H. Weng; G. Lenka; L. C. Tsao; K. C. Chiang; S. T. Pang; T. W. Chen; Y. Y. Jan; M. F. Chen |
| Celiac axis stenosis due to median arcuate ligament compression in a patient who underwent pancreatoduodenectomy; Intraoperative assessment of hepatic arterial flow using Doppler ultrasonography: A case report | 2018 | M. Yamamoto; T. Itamoto; A. Oshita; Y. Matsugu |
| Central hepatobiliary toxicity after liver SBRT and hypo-fractionated Radiotherapy | 2020 | E. Gkika; C. Weis; S. Kirste; F. Mohammad; S. Adebahr; D. Baltas; T. Brunner; A. L. Grosu |
| Central liver toxicity after SBRT: An expanded analysis and predictive nomogram | 2017 | D. A. S. Toesca; E. C. Osmundson; R. V. Eyben; J. L. Shaffer; P. Lu; A. C. Koong; D. T. Chang |
| Changes in production of interleukin-1 and interleukin-2 associated with obstructive jaundice and biliary drainage in patients with gastrointestinal cancer | 1989 | Y. Haga; K. Sakamoto; H. Egami; Y. Yokoyama; M. Arai; K. Mori; M. Akagi |
| Characteristics of congenital hepatic fibrosis in a large cohort of patients with autosomal recessive polycystic kidney disease | 2013 | M. Gunay-Aygun; E. Font-Montgomery; L. Lukose; M. Tuchman Gerstein; K. Piwnica-Worms; P. Choyke; K. T. Daryanani; B. Turkbey; R. Fischer; I. Bernardini; M. Sincan; X. Zhao; N. G. Sandler; A. Roque; D. C. Douek; J. Graf; M. Huizing; J. C. Bryant; P. Mohan; W. A. Gahl; T. Heller |
| Characteristics of Liver Disease in 100 Individuals with Joubert Syndrome Prospectively Evaluated at a Single Center | 2018 | A. Strongin; T. Heller; D. Doherty; I. A. Glass; M. A. Parisi; J. Bryant; P. Choyke; B. Turkbey; K. Daryanani; D. Yildirimli; M. Vemulapalli; J. C. Mullikin; M. C. Malicdan; T. Vilboux; W. A. Gahl; M. Gunay-Aygun |
| Characterization and Prediction of Signal Intensity Changes in Normal Liver Parenchyma on Gadoxetic Acid-enhanced MRI Scans after Liver-directed Radiation Therapy | 2022 | A. D. Nehlsen; K. K. Sindhu; T. Wolken; F. Khan; C. K. Kyriakakos; S. C. Ward; E. Moshier; B. Taouli; M. Buckstein |
| Characterization of patients of different ethnicities with inflammatory bowel disease and primary sclerosing cholangitis | 2010 | O. Damas; D. Jahann; C. Levy |
| Characterization, outcome, and prognosis in 273 patients with primary sclerosing cholangitis: A single center study | 2007 | J. J. W. Tischendorf; H. Hecker; M. Krüger; M. P. Manns; P. N. Meier |
| Chemotherapy versus Best Supportive Care in Advanced Biliary Tract Carcinoma: A Multi-institutional Propensity Score Matching Analysis | 2018 | J. H. Ji; Y. S. Kim; I. Park; S. I. Lee; R. B. Kim; J. O. Park; S. Y. Oh; I. G. Hwang; J.-S. Jang; H.-N. Song; J.-H. Kang |
| Cholangiocarcinoma in liver cirrhosis | 2003 | C. K. Hui; M. F. Yuen; W. K. Tso; I. O. L. Ng; A. O. O. Chan; C. L. Lai |
| Cholangiocarcinoma is associated with a raised enhanced liver fibrosis score independent of primary sclerosing cholangitis | 2019 | F. Saffioti; D. Roccarina; M. Vesterhus; J. R. Hov; W. Rosenberg; M. Pinzani; S. P. Pereira; K. M. Boberg; D. Thorburn |
| Cholangiocarcinoma presenting as linitis plastica with unusual metastases to the psoas muscle and urinary bladder | 2018 | D. Banerjee; S. Raghavapuram; N. E. George; S. Korourian; F. A. Fedda; A. Perisetti; B. Tharian |
| Cholangiocarcinoma presenting as pseudoachalasia and gastroparesis | 2003 | V. K. S. Leung; P. S. Kan; M. S. Lai |
| Cholangiocarcinoma with a paraneoplastic leukemoid reaction mimicking a pyogenic liver abscess | 2015 | H. Ham; H. Y. Kim; K. J. Seo; S. L. Lee; C. W. Kim |
| Cholangiocarcinoma: A diagnostic dilemma on cytology | 2022 | F. Shamsuddin; M. K. Mallik; S. Hussein; A. A. Alali; K. Kapila |
| Cholecystokinin receptor blockade prevents hepatic injury and fibrosis in a murine model of sclerosing cholangitis | 2020 | A. A. Yassin; H. Cao; R. D. Tucker; M. D. Gay; S. Nadella; A. Kruger; J. P. Smith |
| Choledocho-nodal fistula: Uncommon cause of obstructive jaundice in a patient with hcc diagnosed by combined ERCP/EUS | 2021 | M. H. Emara; M. S. Zaghloul; A. M. Mahros; E. H. Emara |
| Chronic liver disease questionnaire to manage patients with chronic liver diseases | 2022 | K. Tajiri; Y. H. Futsukaichi; A. Murayama; M. Minemura; T. Takahara; I. Yasuda |
| Chronic rejection in children: Risk factors, diagnosis and outcome | 2018 | A. Cheng; K. Mysore; K. Hosek; R. Himes; D. Schady; J. Goss; T. Miloh |
| Clear bile (CB) and dark bile (Db) in malignant biliary obstruction with naïve papilla: Does it affect survival? | 2016 | C. Papafragkakis; S. Thaiudom; G. Lanke; R. C. Chakinala; W. Qiao; S. Jaganmohan; J. Lee |
| Clear bile on ERCP for malignant biliary obstruction - Incidental finding or an ominous sign? A comparative study | 2010 | S. Jaganmohan; R. P. Raju; W. A. Ross; M. Javle; G. R. Varadhachary; G. S. Raju; J. Abbruzzese; J. B. Fleming; J. R. Stroehlein; J. H. Lee |
| CLINICAL AND LABORATORY FINDINGS OF CARCINOMA OF THE PANCREAS AND PERIAMPULLARY STRUCTURES | 1984 | Y. T. N. Lee; M. D. Williams |
| Clinical and Surgical Management of Dog with Diffuse Cholangiocarcinoma | 2018 | A. B. Trindade; D. G. Gerardi |
| Clinical application of cytochrome a(+a3) assay of mitochondria from liver specimens: An aid in determining metabolic tolerance of liver remnant for hepatic resection | 1974 | K. Ozawa; Y. Yamaoka; O. Kitamura |
| Clinical application of regional and intermittent hepatic inflow occlusion in laparoscopic hepatectomy | 2022 | L. Shi; B. Luo; Y. Yang; Y. Miao; X. Li; D. Sun; Q. Zhu |
| CLINICAL CHARACTERISTICS OF INTRAHEPATIC CHOLANGIOCARCINOMA IN SPAIN. LIVER CIRRHOSIS AND HIGH AFP ARE NOT ALWAYS HEPATOCELLULAR CARCINOMA | 2016 | C. Rodriguez-Lope; M. Forne; J. Fuentes; M. Reig; V. Andreu; B. Minguez; M. Inarrairaegui; C. Fernandez; M. Roget; A. Lue; I. Ortiz; F. Jimenez; S. Montoliu; M. C. Garre; P. Rendon; M. Rodriguez; J. Crespo; J. Bruix; M. Varela; Geech |
| Clinical effect of ischemic preconditioning prior to hepatectomy | 2017 | X. Lv; Y. Zhou; C. Fang; X. Guo; T. Zhou; Y. Chen |
| Clinical efficacy analysis of transcatheter arterial chemoembolization (TACE) combined with radiofrequency ablation (RFA) in primary liver cancer and recurrent liver cancer | 2019 | Y. Sun; S. Ji; H. Ji; L. Liu; C. Li |
| Clinical Efficacy of Hypofractionated Proton Beam Therapy for Intrahepatic Cholangiocarcinoma | 2022 | T. H. Kim; S. M. Woo; W. J. Lee; J. W. Chun; Y. R. Cho; B. H. Kim; Y. H. Koh; S. S. Kim; E. S. Oh; D. Y. Lee; S. U. Lee; Y. G. Suh; S. H. Moon; J. W. Park |
| Clinical evaluation of percutaneous endovascular radiofrequency ablation for portal vein tumor thrombus: experience in 120 patients | 2023 | Y. L. Kong; J. J. Sun; H. Y. Zhang; Y. Xing; C. Wang; Y. Liu; X. J. He; L. H. Kong; C. L. Liu |
| Clinical factors associated with the survival of patients with intrahepatic cholangiocarcinoma | 2016 | C. F. Ting; W. H. Huang; C. L. Feng; C. J. Yu; C. Y. Peng; W. P. Su; H. C. Lai; K. S. Cheng; P. H. Chuang; J. T. Kao |
| Clinical Features and Genetic Analysis of Pediatric Patients with Alagille Syndrome Presenting Initially with Liver Function Abnormalities | 2018 | Y. Liu; H. Wang; C. Dong; J. X. Feng; Z. H. Huang |
| Clinical features in severe Opisthorchiasis viverrini | 1985 | S. Pungpak; M. Riganti; D. Bunnag; T. Harinasuta |
| Clinical importance and clarification of the WHO subclassification of combined hepatocellular and cholangiocarcinoma | 2019 | H. Nakayama; T. Takayama; Y. Midorikawa; M. Sugitani |
| Clinical outcomes and prognostic factors of hepatic arterial infusion (HAI) chemotherapy combination regimens in 202 patients with advanced cancer metastatic to the liver: The phase I program M. D. Anderson Cancer Center experience | 2010 | C. Vaklavas; A. M. Tsimberidou; S. Wen; S. Fu; D. S. Hong; J. Wheler; A. Naing; C. Uehara; R. A. Wolff; R. Kurzrock |
| Clinical outcomes of patients undergoing antiviral therapy while awaiting liver transplantation | 2017 | J. M. Pascasio; C. Vinaixa; M. T. Ferrer; J. Colmenero; A. Rubin; L. Castells; M. L. Manzano; S. Lorente; M. Testillano; X. Xiol; E. Molina; L. González-Diéguez; E. Otón; S. Pascual; B. Santos; J. I. Herrero; M. Salcedo; J. L. Montero; G. Sánchez-Antolín; I. Narváez; F. Nogueras; Á. Giráldez; M. Prieto; X. Forns; M. C. Londoño |
| Clinical outcomes of stereotactic body radiation therapy for small hepatocellular carcinoma | 2020 | S. Park; J. Jung; B. Cho; S. Y. Kim; S. C. Yun; Y. S. Lim; H. C. Lee; J. Park; J. H. Park; J. H. Kim; S. M. Yoon |
| Clinical outcomes of Y90 radioembolization for recurrent hepatocellular carcinoma following curative resection | 2017 | R. Ali; A. Riaz; A. Gabr; N. Abouchaleh; R. Mora; A. Al Asadi; J. C. Caicedo; M. Abecassis; N. Katariya; H. Maddur; L. Kulik; R. J. Lewandowski; R. Salem |
| Clinical presentation and outcome of hepatic subcapsular fluid collections | 2009 | C. J. Chen; W. H. Chang; S. C. Shih; T. E. Wang; C. W. Chang; M. J. Chen |
| Clinical profile and outcome of primary sclerosing cholangitis: A single-centre experience from western India | 2019 | P. Tibdewal; P. Bhatt; A. Jain; D. Gupta; S. Bhatia; A. Shukla |
| Clinical profile and outcomes of primary sclerosing cholangitis in a tertiary care centre | 2017 | P. S. Tibdewal; P. Bhatt; A. Sadalage; A. Jain; S. Bhatia; A. Shukla |
| Clinical results after biliary drainage by endoscopic retrograde cholangiopancreatography for analysis of metastatic cancer survival and prognostic factors | 2021 | P. Yuan; L. Zhang; S. Li; X. Li; Q. Wu |
| Clinical score to predict the risk of bile leakage after liver resection | 2016 | T. Kajiwara; Y. Midorikawa; S. Yamazaki; T. Higaki; H. Nakayama; M. Moriguchi; S. Tsuji; T. Takayama |
| Clinicohistological correlation of etiological spectrum of chronic liver disease diagnosed during noncirrhotic stages in children: Can need of liver biopsy be obviated? | 2021 | T. Samanta; R. Basu; R. Purkait; S. Kar; D. Das; S. Ganguly |
| Clinicopathological characteristics and diagnostic performance of Wisteria floribunda agglutinin positive Mac-2-binding protein as a preoperative serum marker of liver fibrosis in hepatocellular carcinoma | 2015 | M. Fujiyoshi; A. Kuno; M. Gotoh; M. Fukai; H. Yokoo; H. Kamachi; T. Kamiyama; M. Korenaga; M. Mizokami; H. Narimatsu; A. Taketomi; G. Hepatitis Glyco-biomarker Study |
| Clinicopathological Characteristics of Intrahepatic Cholangiocarcinoma in Patients with Cirrhosis | 2011 | Y.-Y. Li; D.-J. Chen; X.-R. Li; B.-N. Tian; W.-D. Liu; G. Liu; H. Li |
| Clinicopathological difference between invasive pancreatic duct cancer and distal bile duct cancer of the pancreas head after pancreaticoduodenectomy | 2020 | M. Ikeguchi; K. Endo |
| Clinicopathological features and outcomes of gallbladder cancer in southern Pakistan | 2012 | A. A. Burney; S. Memon; A. Ghaffar; A. Salam; Z. Aftab; I. A. Burney |
| Clinicopathological impact of anti-smooth muscle antibodies in patients with non-alcoholic fatty liver disease | 2019 | H. Elalfy; M. A. El-Maksoud; S. Abed; M. A. El Aziz; A. Z. Elsamanoudy; S. M. A. El-khair; M. A. Mohamed; W. Elkashef; K. Zalata; R. Farag; M. Arafa |
| Clinicopathological study in 80 patients with Biliary atresia treated by liver transplantation with and without prior Kasai portoenterostomy | 2018 | N. Vanderheyden; C. De Magnée; X. Stephenne; F. Smets; E. Sokal; R. Reding; M. Komuta |
| Combination of yttrium-90 radioembolization with stereotactic body radiation therapy in the treatment of portal vein tumor thrombosis | 2021 | J. Liu; C. Ladbury; A. Amini; S. Glaser; J. Kessler; A. Lee; Y. J. Chen |
| Combined hepatocellular and cholangiocarcinoma: Clinical features and prognostic study in a Thai population | 2006 | S. Chantajitr; C. Wilasrusmee; P. Lertsitichai; N. Phromsopha |
| Combined hepatocellular cholangiocarcinoma | 2007 | S. M. Nourani; E. Du; P. J. Pockros |
| Combined hepatocellular-cholangiocarcinoma in a patient with Abernethy malformation and tetralogy of Fallot: A case report | 2016 | S. Happaerts; A. Foucault; J. S. Billiard; B. Nguyen; F. Vandenbroucke-Menu |
| Combined hepatocellular-cholangiocarcinoma in liver cirrhosis HBV patient: A case report | 2020 | P. Ismail; K. F. Kalista |
| Combining Preoperative Clinical and Imaging Characteristics to Predict MVI in Hepatitis B Virus-Related Combined Hepatocellular Carcinoma and Cholangiocarcinoma | 2023 | S. S. Huang; M. X. Zuo; C. M. Xie |
| Common bile duct size in patients with cirrhosis | 2011 | C. Wongjitrat; A. Pattanapraphapan; N. Wongjitrat; W. Burivong |
| Comparison between dynamic gadoxetate-enhanced MRI and 99mTc-mebrofenin hepatobiliary scintigraphy with SPECT for quantitative assessment of liver function | 2019 | F. Rassam; T. Zhang; K. P. Cieslak; C. Lavini; J. Stoker; R. J. Bennink; T. M. van Gulik; L. J. van Vliet; J. H. Runge; F. M. Vos |
| Comparison between Endoscopic Stenting Alone versus Balloon Dilatation plus Stenting in Patients with Benign Common Bile Duct Strictures | 2022 | A. F. G. Abd-El-razik; A. M. El-Sharif; M. A. E. K. Eldahshan; Z. M. Zakarya; H. M. Abd-Elmageed; A. E. H. A. Mahmoud |
| Comparison of calculated body muscle mass and sarc-f as methods of screening for sarcopenia in patients with chronic liver disease | 2021 | T. Ichikawa; H. Miyaaki; S. Miuma; Y. Motoyoshi; M. Yamashima; S. Yamamichi; M. Koike; Y. Nakano; T. Honda; H. Yajima; R. Uehara; O. Miyazaki; Y. Kuribayashi; K. Kira; N. Taura; K. Nakao |
| Comparison of Choi criteria and Response Evaluation Criteria in Solid Tumors (RECIST) for intrahepatic cholangiocarcinoma treated with glass-microspheres Yttrium-90 selective internal radiation therapy (SIRT) | 2016 | L. Beuzit; J. Edeline; V. Brun; M. Ronot; A. Guillygomarc'H; K. Boudjema; Y. Gandon; E. Garin; Y. Rolland |
| Comparison of inflammation-based prognostic scores in patients with biliary tract cancer after surgical resection | 2021 | M. Utsumi; K. Kitada; N. Tokunaga; Y. Yoshida; T. Narusaka; R. Hamano; H. Miyasou; Y. Tsunemitsu; S. Otsuka; M. Inagaki |
| Comparison of Liver Fibrosis and Function Indices with Extracellular Volume Using Dual-energy CT: A Retrospective Study | 2022 | R. Kokubo; K. Saito; T. Yamada; T. Tanaka; Y. Tajima; K. Suzuki |
| Comparison of liver transplantation outcome in patients with primary sclerosing cholangitis primary biliary cirrhosis, and secondary biliary cirrhosis | 2010 | H. Sogawa; R. Arnon; R. A. Annunziato; T. A. Miloh; T. D. Schiano |
| Comparison of percutaneous transhepatic portal vein embolization and unilateral portal vein ligation | 2012 | H. Iida; T. Aihara; S. Ikuta; H. Yoshie; N. Yamanaka |
| Comparison of postoperative early and late complications between pancreas-sparing duodenectomy and pancreatoduodenectomy | 2017 | Y. Nakayama; M. Konishi; N. Gotohda; Y. Kato; H. Aizawa; M. Kudo; S. Okubo; D. Takahashi; Y. Nishida; K. Kitaguchi; S. Takahashi |
| Comparison of procedure-related complications between percutaneous cryoablation and radiofrequency ablation for treating periductal hepatocellular carcinoma | 2020 | S. E. Ko; M. W. Lee; H. Rhim; T. W. Kang; K. D. Song; D. I. Cha; H. K. Lim |
| Comparison of Seven Noninvasive Models for Predicting Decompensation and Hospitalization in Patients with Cirrhosis | 2021 | C. Y. Hsu; N. D. Parikh; T. I. Huo; E. B. Tapper |
| Comparison of the Clinical Features between Primary Biliary Cirrhosis and Drug-induced Liver Injury | 2018 | H. Wang |
| Compelling Long-Term Results for Liver Resection in Early Cholangiocarcinoma | 2021 | J. Bednarsch; Z. Czigany; L. R. Heij; D. Liu; M. den Dulk; G. Wiltberger; P. Bruners; T. F. Ulmer; U. P. Neumann; S. A. Lang |
| Complications after liver transplantation | 2013 | Z. S. Yu; Y. Jiang; Q. C. Cai; F. Yang; L. Z. Lü |
| Complications of percutaneous liver biopsy with Klatskin needles: a 36-year single-centre experience | 2017 | V. Takyar; O. Etzion; T. Heller; D. E. Kleiner; Y. Rotman; M. G. Ghany; N. Fryzek; V. H. Williams; E. Rivera; S. Auh; T. J. Liang; J. H. Hoofnagle; C. Koh |
| Comprehensive metabolome analysis for the pharmacological action of inchinkoto, a hepatoprotective herbal medicine | 2021 | H. Yamashita; K. Ohbuchi; M. Nagino; T. Ebata; K. Tsuchiya; H. Kushida; Y. Yokoyama |
| Concentration of Biliary Extracellular Vesicles Discriminates between Dominant Strictures in Primary Sclerosing Cholangitis and Cholangiocellular Carcinoma | 2018 | T. Worst; K. H. Weiss; B. Goeppert; P. Schirmacher; D. N. Gotthardt; P. Sauer; C. Rupp |
| CONCOMITANT EXTRA-HEPATIC, NON-IBD AUTOIMMUNE DISEASE IS ASSOCIATED WITH REDUCED TRANSPLANTATION-FREE SURVIVAL IN PATIENTS WITH PRIMARY SCLEROSING CHOLANGITIS | 2011 | C. Rupp; A. Mummelthei; P. Sauer; K. H. Weiss; P. Schirmacher; A. Stiehl; W. Stremmel; D. Gotthardt |
| Contrast-enhanced ultrasound manifestations of synchronous combined hepatocellular-cholangiocarcinoma and hepatocellular carcinoma: A case report | 2022 | L. Gao; J. Y. Huang; Z. J. Lu; Q. Lu |
| Contribution of molecular adsorbent recirculating system dialysis (mars®) in the preoperative management of jaundiced patients with hilar cholangiocarcinoma | 2012 | J. M. Regimbeau; E. Chapuis-Roux; D. Fuks; T. Yzet; R. Delcenserie; E. Lobjois; E. N'Guyen-Khac; J. M. Regimbeau |
| Contribution of resident stem cells to liver and biliary tree regeneration in human diseases | 2018 | D. Overi; G. Carpino; V. Cardinale; A. Franchitto; S. Safarikia; P. Onori; D. Alvaro; E. Gaudio |
| Correlation between hepatic oxidative damage and clinical severity and mitochondrial gene sequencing results in biliary atresia | 2019 | J. Wang; J. Xu; M. Xia; Y. Yang; Z. Shen; G. Chen; R. Dong; S. Zheng |
| Correlation between Liver Elasticity by Ultrasound Elastography and Liver Functional Reserve | 2019 | R. Sugiura; M. Kuwatani; M. Nishida; K. Hirata; I. Sano; S. Kato; K. Kawakubo; M. Nakai; T. Sho; G. Suda; K. Morikawa; K. Ogawa; N. Sakamoto |
| Correlation between plasma coagulation factors and some other findings in patients with obstructive jaundice | 1974 | M. Schneider; K. Schulz; W. Teichmann |
| Correlation of native liver parenchyma t1 and t2 relaxation times and liver synthetic function tests: A pilot study | 2021 | U. L. Fahlenkamp; J. Kunkel; K. Ziegeler; K. Neumann; L. C. Adams; G. Engel; S. M. Böker; M. R. Makowski |
| Coupled plasma filtration absorption (CPFA) in one case of hepatocellular and obstructive jaundice | 2018 | L. Meletti; I. Baragetti; E. Alberghini; L. Buzzi; G. Santagostino; S. Furiani; F. Ferrario; V. La Milia |
| COVID-19: Gastrointestinal manifestations, liver injury and recommendations | 2022 | Z. Ozkurt; Z. Ozkurt; E. Ç. Tanrıverdi |
| Crohn's disease complicated by pancreatic atrophy | 2011 | A. Srinath; A. Goyal |
| Cronkhite-Canada syndrome complicated by triple primary cancers | 2016 | K. Yamanouchi; Y. Sakata; N. Tsuruoka; R. Shimoda; M. Uchida; T. Akutagawa; S. Shirai; K. Fujimoto; R. Iwakiri |
| CT-Based Radiomics Analysis for Noninvasive Prediction of Perineural Invasion of Perihilar Cholangiocarcinoma | 2022 | P. C. Zhan; P. J. Lyu; Z. Li; X. Liu; H. X. Wang; N. N. Liu; Y. Zhang; W. Huang; Y. Chen; J. B. Gao |
| Cumulative damage effect of jaundice may be an effective predictor of complications in patients undergoing radical resection of Bismuth type II or above hilar cholangiocarcinoma | 2021 | L. Luo; Y. Yao; H. Liao; J. Huang; M. Liao; J. Wang; K. Yuan; Y. Zeng |
| Curcumin-loaded nanocomplexes: Acute and chronic toxicity studies in mice and hamsters | 2021 | C. Jantawong; A. Priprem; K. Intuyod; C. Pairojkul; P. Pinlaor; S. Waraasawapati; I. Mongkon; Y. Chamgramol; S. Pinlaor |
| Current Approaches and Future Directions for the Treatment of mTORopathies | 2021 | V. Karalis; H. S. Bateup |
| Cytological diagnosis of biliary cryptococcosis in an immunocompromised patient with mid common bile duct stricture masquerading as cholangiocarcinoma | 2017 | D. K. Burad; B. Ramakrishna |
| Cytoreductive surgery combined with hyperthermic intraperitoneal chemotherapy vs. cytoreductive surgery alone for intrahepatic cholangiocarcinoma with peritoneal metastases: A retrospective cohort study | 2021 | F. Feng; Q. Gao; Y. Wu; C. Liu; Y. Yu; B. Li; K. Chu; B. Yi; Q. Cheng; X. Jiang |
| Defining the Safety Profile for Performing Pancreatoduodenectomy in the Setting of Hyperbilirubinemia | 2020 | B. Chen; M. T. Trudeau; L. Maggino; B. L. Ecker; L. J. Keele; R. P. DeMatteo; J. A. Drebin; D. L. Fraker; M. K. Lee; R. E. Roses; C. M. Vollmer |
| Delayed gastric emptying after standard pancreaticoduodenectomy versus pylorus-preserving pancreaticoduodenectomy: An analysis of 200 consecutive patients | 1997 | M. I. Van Berge Henegouwen; T. M. Van Gulik; L. T. DeWit; J. H. Allema; E. A. J. Rauws; H. Obertop; D. J. Gouma |
| Denosumab-Induced Immune Hepatitis | 2021 | V. Ostrovsky; S. Malnick; S. Ish-Shalom; N. Ziv Sokolowskaia; A. Yosepovich; M. Neuman |
| Detection of serum alpha-fetoprotein in dogs with hepatic tumors | 1991 | L. A. Lowseth; N. A. Gillett; I. Y. Chang; B. A. Muggenburg; B. B. Boecker |
| Development and external validation of preoperative risk models for operative morbidities after total gastrectomy using a Japanese web-based nationwide registry | 2017 | H. Kikuchi; H. Miyata; H. Konno; K. Kamiya; A. Tomotaki; M. Gotoh; G. Wakabayashi; M. Mori |
| Development and internal validation of laboratory prognostic score to predict 14-day mortality in terminally ill patients with gastrointestinal malignancy | 2022 | H. Nagai; N. Kawai; N. Yuasa |
| Development and performance assessment of novel machine learning models to predict pneumonia after liver transplantation | 2021 | C. Chen; D. Yang; S. Gao; Y. Zhang; L. Chen; B. Wang; Z. Mo; Y. Yang; Z. Hei; S. Zhou |
| Development and Preliminary Testing of a Translational Model of Hepatocellular Carcinoma for MR Imaging and Interventional Oncologic Investigations | 2012 | S. M. Thompson; M. R. Callstrom; B. Knudsen; J. L. Anderson; R. E. Carter; J. P. Grande; L. R. Roberts; D. A. Woodrum |
| Development and validation of a 90-day mortality prediction model following endobiliary drainage in patients with unresectable malignant biliary obstruction | 2022 | P. Termsinsuk; P. Charatcharoenwitthaya; N. Pausawasdi |
| Development and Validation of a Nomogram for Differentiating Combined Hepatocellular Cholangiocarcinoma From Intrahepatic Cholangiocarcinoma | 2020 | T. Wang; W. Wang; J. Zhang; X. Yang; S. Shen; W. Wang |
| Development and Validation of a Nomogram Model to Predict the Prognosis of Intrahepatic Cholangiocarcinoma | 2022 | Y. Chen; L. Huang; Z. Wei; X. Liu; L. Chen; B. Wang |
| Development and Validation of a Prognostic Model to Predict Recurrence-Free Survival After Curative Resection for Perihilar Cholangiocarcinoma: A Multicenter Study | 2022 | Z. P. Liu; W. Y. Chen; Z. R. Wang; X. C. Liu; H. N. Fan; L. Xu; Y. Pan; S. Y. Zhong; D. Xie; J. Bai; Y. Jiang; Y. Q. Zhang; H. S. Dai; Z. Y. Chen |
| Development and validation of a risk score for predicting clinical success after endobiliary stenting for malignant biliary obstruction | 2022 | N. Pausawasdi; P. Termsinsuk; P. Charatcharoenwitthaya; J. Limsrivilai; U. Kaosombatwattana |
| DHEA Protects Human Cholangiocytes and Hepatocytes against Apoptosis and Oxidative Stress | 2022 | E. Kilanczyk; D. Ruminkiewicz; J. M. Banales; P. Milkiewicz; M. Milkiewicz |
| Diabetes and PKM2 affect prognosis in patients with intrahepatic cholangiocarcinoma | 2020 | F. U. Kui; X. Yang; W. U. Hao; J. Gong; L. I. Xiuyun |
| Diagnosis and management of biliary obstruction | 2012 | P. J. Laing; D. G. Adler |
| Diagnostic and surgical therapeutic features of extrahepatic bile duct carcinoma without jaundice | 2004 | H.-H. Tang; S. Chang; X.-W. Wang; Y. Huang; X.-J. Gong; J. Zhou |
| Diagnostic benefit of cytological and histopathological examinations for recurrent vaginal cancer metastasizing to the duodenum: A case report | 2020 | K. Sonoda; M. Okadome; R. Sugimoto; T. Fujimoto; K. Taguchi; T. Saito |
| Dickkopf-1 and amphiregulin as novel biomarkers and potential therapeutic targets in hepatocellular carcinoma | 2019 | A. E. Awad; M. A. Ebrahim; L. A. Eissa; M. M. El-Shishtawy |
| Different clinical and genetic features of Alagille patients with progressive disease versus a jaundice-free course | 2022 | C. M. Chiang; Y. M. Jeng; M. C. Ho; M. W. Lai; H. Y. Li; P. L. Chen; N. C. Lee; J. F. Wu; Y. C. Chiu; B. Y. Liou; Y. H. Ni; H. Y. Hsu; M. H. Chang; H. L. Chen |
| Differential diagnoses in 83 dogs with icterus | 2020 | M. C. Andrade; L. B. Oliveira; Á. F. Santos; M. V. L. Moreira; F. Pierezan; R. Ecco |
| Differential diagnosis of chronical elevated liver parameters | 2008 | B. Rodeck |
| Direct Hyperbilirubinemia in an Infant | 2017 | M. A. Magyar; D. Metropulos; J. W. Antoon |
| Distinctive Morphologic Pattern and In Situ Hybridization for Albumin Distinguishes Intrahepatic Cholangiocarcinoma from Metastatic Adenocarcinoma | 2016 | G. Acosta-Gonzalez; M. Ettel; O. Eze; S. Gera; C. H. Hajdu; J. S. Park; S. Sigal; R. Xu |
| Dithiothreitol supplementation mitigates hepatic and renal injury in bile duct ligated mice: Potential application in the treatment of cholestasis-associated complications | 2018 | R. Heidari; V. Ghanbarinejad; H. Mohammadi; A. Ahmadi; A. Esfandiari; N. Azarpira; H. Niknahad |
| DLBCL Mimicking a Cholangiocarcinoma: Earlier Diagnosis, Better Outcome? | 2019 | A. Khadilkar; C. Mateja; S. Thomas |
| Do Biliary Complications after Proton Beam Therapy for Perihilar Hepatocellular Carcinoma Matter? | 2020 | G. S. Yoo; J. Il Yu; H. C. Park; D. Hyun; W. K. Jeong; H. Y. Lim; M. S. Choi; S. Y. Ha |
| Does Pre-operative Biliary Drainage Influence Long-Term Survival in Patients With Obstructive Jaundice With Resectable Pancreatic Head Cancer? | 2020 | Z. Shen; J. Zhang; H. Chen; W. Wang; W. Xu; X. Lu; Y. Zhou; S. Zhao; Z. Xu; X. Deng; J. Wang; Y. Weng; B. Shen |
| Double plastic stenting for inoperable malignant biliary stricture among cirrhotic patients as a possible cost-effective treatment: A pilot study | 2019 | M. I. Radwan; M. H. Emara; M. S. Zaghloul; A. M. S. Zaghloul |
| Drug-induced sarcoidosis-like reaction three months after BNT162b2 mRNA COVID-19 vaccination: A case report and review of literature | 2023 | S. R. Kim; S. K. Kim; T. Fujii; H. Kobayashi; T. Okuda; T. Hayakumo; A. Nakai; Y. Fujii; R. Suzuki; N. Sasase; A. Otani; Y. I. Koma; M. Sasaki; T. Kumabe; O. Nakashima |
| Duct reveals architectural mechanisms contributing to bile duct recovery in a mouse model for alagille syndrome | 2021 | S. Hankeova; J. Salplachta; T. Zikmund; M. Kavkova; N. Van Hul; A. Brinek; V. Smekalova; J. Laznovsky; F. Dawit; J. Jaros; V. Bryja; U. Lendahl; E. Ellis; A. Nemeth; B. Fischler; E. Hannezo; J. Kaiser; E. R. Andersson |
| Duodenal diverticulum metastasis from pulmonary squamous cell carcinoma diagnosed by EUS-FNA | 2022 | K. Sunaga; T. Yoshioka; M. Shigekawa; K. Sato; R. Sakamori; T. Tatsumi; K. Ukon; E. Morii; T. Takehara |
| Durability of virologic response, risk of de novo hepatocellular carcinoma, liver function and stiffness 2 years after treatment with ombitasvir/paritaprevir/ritonavir±dasabuvir±ribavirin in the AMBER, real-world experience study | 2018 | R. Flisiak; E. Janczewska; M. Łucejko; E. Karpińska; D. Zarębska-Michaluk; K. Nazzal; B. Bolewska; J. Białkowska; H. Berak; K. Fleischer-Stępniewska; K. Tomasiewicz; K. Karwowska; K. Simon; A. Piekarska; O. Tronina; E. Tuchendler; A. Garlicki |
| Early Diagnosis of a Life-threatening Cause of Obstructive Jaundice | 2017 | L. D. M. Concepcion; J. Martin; A. F. Perez |
| Early experience of atezolizumab plus bevacizumab treatment for unresectable hepatocellular carcinoma BCLC-B stage patients classified as beyond up to seven criteria – Multicenter analysis | 2022 | A. Hiraoka; T. Kumada; T. Tada; M. Hirooka; K. Kariyama; J. Tani; M. Atsukawa; K. Takaguchi; E. Itobayashi; S. Fukunishi; K. Tsuji; T. Ishikawa; K. Tajiri; H. Ochi; S. Yasuda; H. Toyoda; C. Ogawa; T. Nishimura; T. Hatanaka; S. Kakizaki; N. Shimada; K. Kawata; A. Naganuma; T. Tanaka; H. Ohama; K. Nouso; A. Morishita; A. Tsutsui; T. Nagano; N. Itokawa; T. Okubo; T. Arai; M. Imai; Y. Koizumi; S. Nakamura; K. Joko; H. Iijima; Y. Hiasa; M. Kudo |
| Early hepatocellular carcinoma developed within dysplastic nodule as nodule-within-nodule appearance: Case report with literature review | 2022 | F. Aassouani; F. Z. Lkharrat; Y. Charifi; A. Attar; M. Lahlali; N. El Bouardi; H. Abid; M. Haloua; B. Alami; M. Boubbou; M. Maaroufi; M. Y. A. Lamrani |
| Early percutaneous catheter drainage in protecting against prolonged fever among patients with pyogenic liver abscess: a retrospective cohort study | 2022 | Y. Liu; Z. Li; A. Liu; J. Xu; Y. Li; J. Liu; Y. Liu; H. Zhu |
| Early postoperative serum aspartate aminotransferase for prediction of post-hepatectomy liver failure | 2022 | W. Vassanasiri; N. Rungsakulkij; W. Suragul; P. Tangtawee; P. Muangkaew; S. Mingphruedhi; S. Aeesoa |
| Early response and safety of lenvatinib for patients with advanced hepatocellular carcinoma in a real-world setting | 2020 | T. Sho; G. Suda; K. Ogawa; M. Kimura; T. Shimazaki; O. Maehara; T. Shigesawa; K. Suzuki; A. Nakamura; M. Ohara; M. Umemura; N. Kawagishi; M. Natsuizaka; M. Nakai; K. Morikawa; K. Furuya; M. Baba; Y. Yamamoto; T. Kobayashi; T. Meguro; A. Saga; T. Miyagishima; H. Yokoo; T. Kamiyama; A. Taketomi; N. Sakamoto |
| Economic evaluation of palliative biliary drainage in unresectable hilar cholangiocarcinoma | 2018 | T. Suttichaimongkol; S. Borntrakulpipat; A. Sangchan; P. Mairiang; E. Mairiang; W. Sukeepaisarnjaroen; K. Chunlertlith; K. Sawadpanich |
| EFFECT OF BILIARY DECOMPRESSION ON MORBIDITY AND MORTALITY OF PANCREATODUODENECTOMY | 1984 | J. H. Thomas; C. S. Connor; G. E. Pierce; R. I. Macarthur; J. I. Iliopoulos; A. S. Hermreck |
| Effect of Biliary Tract Invasion with Obstructive Jaundice on the Prognosis of Patients With Unresectable Hepatocellular Carcinoma | 2023 | W. Jiang; X. Fu; G. Wang; H. Qi; Z. Chen; F. Gao |
| Effect of delta α-fetoprotein on the detection of liver cancer recurrence | 2020 | L. Y. Sun; Y. He; Q. Liu; F. Wang |
| Effect of percutaneous transhepatic cholangial drainag plus radiofrequency ablation combined with biliary stent implantation on the liver function of patients with cholangiocarcinoma complicated with malignant obstructive jaundice | 2021 | S. Qi; H. Yan |
| Effect of preoperative biliary drainage on liver function changes in patients with malignant obstructive jaundice in the low bile duct before and after pancreaticoduodenectomy | 2008 | D. Chen; L. J. Liang; B. G. Peng; Q. Zhou; S. Q. Li; D. Tang; L. Huang; J. F. Huang |
| Effect of preoperative biliary drainage on surgical outcome after pancreaticoduodenectomy | 2006 | Y.-F. Tsai; J.-F. Shyu; T.-H. Chen; Y.-M. Shyr; C.-H. Su |
| Effect of pre-operative bilirubin levels on post-operative mortality and morbidity after pancreatico-duodenectomy for pancreatic cancer. An analysis of 4850 patients from NSQIP database | 2014 | P. Aggarwal; D. Marx; K. Turaga; A. Forse |
| Effect of PTCD-based biliary stent placement combined with 125I particle intracavitary irradiation in treating pancreatic head cancer | 2020 | J. Cao; Z. Wang; H. Cai; J. Zhang; Y. Yue; X. Liu; D. Zhang |
| Effect of radical resection combined with antiviral therapy in patients with hepatitis B virus-associated hepatocellular carcinoma and prognostic analysis | 2021 | C. Xiao; L. Huang; N. Xiao; J. Lun |
| Effect of Remote Ischemic Preconditioning in Patients Undergoing Hepatectomy With Portal Triad Clamping: A Randomized Controlled Trial | 2019 | X. Liu; L. Cao; T. Zhang; R. Guo; W. Lin |
| Effective albumin concentration predicts survival with native liver in cirrhotic children | 2022 | V. Gautam; R. Khanna; B. Mathew; G. Tripathi; A. Saini; V. Sood; B. Bihari Lal; S. Sarah Thomas; J. Maras; S. Alam |
| Effectiveness and Toxicity of Yttrium-90 Microsphere Brachytherapy | 2014 | C. J. Baden; J. D. Roberson; R. Jacob; O. L. Burnett |
| Effectiveness of drug-eluting bead transarterial chemoembolization versus conventional transarterial chemoembolization for small hepatocellular carcinoma in Child-Pugh class A patients | 2019 | I. J. Lee; J. H. Lee; Y. B. Lee; Y. J. Kim; J. H. Yoon; Y. H. Yin; M. Lee; S. Hur; H. C. Kim; H. J. Jae; J. W. Chung |
| Effectiveness of endoscopic biliary drainage for unresectable hepatocellular carcinoma associated with obstructive jaundice | 2001 | K. Matsueda; H. Yamamoto; F. Umeoka; T. Ueki; T. Matsumura; T. Tezen; I. Doi |
| Effectiveness of Repeated Stereotactic Body Radiation Therapy for Hepatocellular Carcinoma—Consideration of the Locations of Target Lesions | 2023 | S. Yano; T. Kimura; T. Kawaoka; T. Kinami; S. Yamasaki; Y. Johira; M. Kosaka; K. Amioka; K. Naruto; Y. Ando; K. Yamaoka; Y. Fujii; S. Uchikawa; H. Fujino; A. Ono; T. Nakahara; E. Murakami; W. Okamoto; M. Yamauchi; M. Imamura; J. Hirokawa; Y. Nagata; H. Aikata; S. Oka |
| Effectiveness of the ligasure small jaw vessel-sealing system in hepatic resection | 2014 | M. Yoshimoto; K. Endo; T. Hanaki; J. Watanabe; N. Tokuyasu; T. Sakamoto; S. Honjo; Y. Hirooka; M. Ikeguchi |
| Effects of biliary obstruction on hepatic deoxyribonucleic acid and protein synthesis after partial hepatectomy | 1997 | F. Kimura; M. Miyazaki; H. Itoh |
| Effects of body mass index and serum albumin on overall survival in patients with cancer undergoing pancreaticoduodenectomy: a single-center retrospective cohort study | 2022 | L. Cui; H. Yu; Q. Sun; Y. Miao; K. Jiang; X. Fang |
| Effects of systemic chemotherapy on the liver | 2010 | G. Ramadori; S. Cameron |
| Efficacy and safety analysis of enhanced recovery after partial hepatectomy for hepatocellular carcinoma: A controlled study with propensity score matching | 2018 | L. Ma; X. H. Zhao; S. L. Zhu; L. Q. Li; B. D. Xiang |
| Efficacy and safety of CalliSpheres drug-eluting bead embolization for treating spontaneous rupture and hemorrhage of primary hepatocellular carcinoma | 2022 | Y. Zhou; Z. Fang; Y. Tang; S. Wu; S. Cai; H. Yang |
| Efficacy and safety of CalliSPHEres® drug-eluting beads transarterial chemoembolization in Barcelona clinic liver cancer stage C patients | 2019 | Y. Liu; W. Huang; M. He; H. Lian; Y. Guo; J. Huang; J. Zhou; K. Zhu |
| Efficacy and Safety of Drug-Eluting Beads Transarterial Chemoembolization Combining Immune Checkpoint Inhibitors in Unresectable Intrahepatic Cholangiocarcinoma: A Propensity Score Matching Analysis | 2022 | X. G. Yang; Y. Y. Sun; D. S. Li; G. H. Xu; X. Q. Huang |
| Efficacy and Safety of Sorafenib in Unresectable Hepatocellular Carcinoma with Bile Duct Invasion | 2020 | T. Tanaka; T. Kuzuya; M. Ishigami; T. Ito; Y. Ishizu; T. Honda; T. Ishikawa; M. Fujishiro |
| Efficacy of Artificial Liver Support System in Severe Immuneassociated Hepatitis Caused by Camrelizumab: A Case Report and Review of the Literature | 2021 | Y. W. Tan; L. Chen; X. B. Zhou |
| Efficacy of Cyproheptadine Monotherapy in Hepatocellular Carcinoma With Bone Metastasis: A Case Report | 2021 | Y. M. Feng; T. H. Chen; D. Berman; C. K. Chou; K. S. Liao; M. C. Hsieh; C. Y. Chen |
| Efficacy of gadoxetic acid-enhanced magnetic resonance cholangiography compared with T2-weighted magnetic resonance cholangiography in patients with liver cirrhosis | 2015 | W. P. Wu; R. C. Chen; C. W. Lee; Y. L. Chen; K. W. Lee; H. K. Wu; C. T. Chou |
| Efficacy of hepatic resection vs transarterial chemoembolization for solitary huge hepatocellular carcinoma | 2015 | S. L. Zhu; J. H. Zhong; Y. Ke; L. Ma; X. M. You; L. Q. Li |
| Efficacy of radiofrequency ablation for initial recurrent hepatocellular carcinoma after curative treatment: Comparison with primary cases | 2015 | T. Fukuhara; H. Aikata; H. Hyogo; Y. Honda; K. Morio; R. Morio; M. Hatooka; T. Kobayashi; N. Naeshiro; T. Kawaoka; M. Tsuge; A. Hiramatsu; M. Imamura; Y. Kawakami; K. Chayama |
| Elevated CA 19-9 associated with heavy black tea consumption | 2019 | A. C. Jiang; E. Y. Chan; S. Jakate; N. Shah |
| Emerging indications for MARS® dialysis | 2002 | G. Schachschal; S. Morgera; S. Küpferling; H. H. Neumayer; H. Lochs; H. H. J. Schmidt |
| Endoluminal radiofrequency ablation with SpyGlass™ in the management of cholangiocarcinoma | 2019 | R. Mansilla-Vivar; L. Argüello-Viúdez; C. Sánchez-Montes; N. Alonso-Lázaro; V. P. Beltrán |
| Endoscopic biliary drainage in unresectable biliary obstruction: The role of endoscopic ultrasound-guidance in a cohort study | 2019 | M. Puga; N. Pallarès; J. Velásquez-Rodríguez; A. García-Sumalla; C. F. Consiglieri; J. Busquets; B. Laquente; M. Calvo; J. Fabregat; J. Castellote; J. B. Gornals |
| Endoscopic diagnosis of common bile duct varices by percutaneous trans- hepatic choledochoscopy: Differential diagnosis from bile duct carcinoma | 1994 | T. Ikegami; Y. Matsuzaki; Y. Saito; M. Nishi; N. Tanaka; T. Osuga; K. Orii; K. Fukao; Y. Iwasaki; H. Matsumoto |
| Endoscopic Management of Choledochoduodenal Fistula Presenting as Pneumobilia | 2017 | E. Golikov; M. Sheinman; S. Salem |
| Endoscopic palliation and prognostication of malignant hilar biliary strictures: A single centre experience | 2021 | W. On; M. A. Saleem; V. S. Hegade; M. T. Huggett; B. Paranandi; S. Everett |
| Endoscopic palliation and survival in malignant biliary obstruction | 2001 | S. A. Weaver; B. S. F. Stacey; S. J. Hayward; G. J. Taylor; N. I. Rooney; D. A. F. Robertson |
| Endoscopic therapy for biliary tract disease before orthotopic liver transplantation | 2001 | R. Shrestha; H. Lasch |
| Endoscopic-catheter-directed infusion of diluted (-)-noradrenaline for atypical hemobilia caused by liver abscess: A case report | 2022 | H. Zou; Y. Wen; Y. Pang; H. Zhang; L. Zhang; L. J. Tang; H. Wu |
| Enhanced Liver Fibrosis Score Predicts Transplant-Free Survival in Primary Sclerosing Cholangitis | 2015 | M. Vesterhus; J. R. Hov; A. Holm; E. Schrumpf; S. Nygård; K. Godang; I. M. Andersen; S. Næss; D. Thorburn; F. Saffioti; M. Vatn; O. H. Gilja; F. Lund-Johansen; T. Syversveen; K. Brabrand; A. Parés; C. Y. Ponsioen; M. Pinzani; M. Färkkilä; B. Moum; T. Ueland; H. Røsjø; W. Rosenberg; K. M. Boberg; T. H. Karlsen |
| Epidemiological Characteristics of Primary Liver Cancer in Mainland China From 2003 to 2020: A Representative Multicenter Study | 2022 | J. Lin; H. Zhang; H. Yu; X. Bi; W. Zhang; J. Yin; P. Zhao; X. Liang; C. Qu; M. Wang; M. Hu; K. Liu; Y. Wang; Z. Zhou; J. Wang; X. Tan; W. Liu; Z. Shao; J. Cai; W. Tang; G. Cao |
| Epidemiology of primary sclerosing cholangitis in inflammatory bowel disease: A longitudinal saudi cohort study | 2017 | N. A. Azzam; O. Alharbi; A. Aljebreen; M. Mosli; N. Musibeeh; M. Alowais; A. Alshaya; M. Almadi |
| Erdheim-Chester Disease (ECD): A rare cause of cholestatic hepatitis | 2010 | V. Oza; N. Mehta; C. Anand |
| Establishment of a pretreatment nomogram to predict the 6-month mortality rate of patients with advanced biliary tract cancers undergoing gemcitabine-based chemotherapy | 2021 | C. E. Wu; W. K. Huang; W. C. Chou; C. H. Hsieh; J. W. C. Chang; C. Y. Lin; C. N. Yeh; J. S. Chen |
| Establishment of risk prediction model of postoperative pancreatic fistula after pancreatoduodenectomy: 2016 edition of definition and grading system of pancreatic fistula: a single center experience with 223 cases | 2021 | J. Yu; C. Y. Ren; J. Wang; W. Cui; J. J. Zhang; Y. J. Wang |
| Estimate of hepatocellular carcinoma incidence in patients with alcoholic cirrhosis | 2018 | N. Ganne-Carrié; C. Chaffaut; V. Bourcier; I. Archambeaud; J. M. Perarnau; F. Oberti; D. Roulot; C. Moreno; A. Louvet; T. Dao; R. Moirand; O. Goria; E. Nguyen-Khac; N. Carbonell; T. Antonini; S. Pol; V. de Ledinghen; V. Ozenne; J. Henrion; J. M. Péron; A. Tran; G. Perlemuter; X. Amiot; J. P. Zarski; M. Beaugrand; S. Chevret |
| Estrogen can stimulate cholangiocarcinoma cells proliferation and invasion and associate with poor prognosis | 2011 | P. Thuwajit; T. Hunsawong; C. Thuwajit |
| Estrogen is increased in male cholangiocarcinoma patients' serum and stimulates invasion in cholangiocarcinoma cell lines in vitro | 2012 | T. Hunsawong; E. Singsuksawat; N. In-chon; W. Chawengrattanachot; C. Thuwajit; B. Sripa; A. Paupairoj; S. Chau-in; P. Thuwajit |
| Evaluation for hepatic diseases in a primary care setting | 2002 | M. Elhajj; I. Elhajj |
| Evaluation of 2D- shear wave elastography for characterisation of focal liver lesions | 2017 | L. Gerber; D. Fitting; K. Srikantharajah; N. Weiler; G. Kyriakidou; J. Bojunga; F. Schulze; D. Bon; S. Zeuzem; M. Friedrich-Rust |
| Evaluation of abnormal liver function tests | 1996 | R. H. Moseley |
| Evaluation of abnormal liver tests | 2014 | T. A. Woreta; S. A. Alqahtani |
| Evaluation of jaundice in adults | 2017 | M. V. Fargo; S. P. Grogan; A. Saguil |
| Evaluation of Liver Function and the Role of Biliary Drainage before Major Hepatic Resections | 2021 | Y. Yamamoto |
| Evaluation of liver function in patients with liver cirrhosis and chronic liver disease using functional liver imaging scores at different acquisition time points | 2022 | G. Tang; J. Liu; P. Liu; F. Huang; X. Shao; Y. Chen; A. Xie |
| Evaluation of Preoperative Inflammation-Based Prognostic Scores in Patients With Intrahepatic Cholangiocarcinoma: A Multicenter Cohort Study | 2021 | C. He; C. Zhao; J. Lu; X. Huang; C. Chen; X. Lin |
| Evaluation of reserved hepatic function in patients with hepatobiliary tumor by 99mTc-GSA: Effect of hyperbilirubinemia and usefulness of regional reserved hepatic functional imaging | 1996 | J. Wu; N. Ishikawa; T. Takeda; M. Sato; K. Fukunaga; T. Todoroki; T. Okumura; R. Hatakeyama; Y. Itai |
| Evaluation of the safety and pathological effects of neoadjuvant full-dose gemcitabine combination radiation therapy in patients with biliary tract cancer | 2015 | S. Kobayashi; A. Tomokuni; K. Gotoh; H. Takahashi; H. Akita; S. Marubashi; T. Yamada; T. Teshima; K. Nishiyama; M. Yano; H. Ohigashi; O. Ishikawa; M. Sakon |
| Evaluation of the Utility of Prognostic Models for Patients Diagnosed with Peri-hilar Cholangiocarcinoma | 2021 | N. Manu; N. Bird; T. Gilbert; M. QuinnL; S. Fenwick; R. Diaz-Nieto; R. jones; H. Malik |
| Evaluation of thyroid function tests in children with chronic liver diseases | 2020 | Ş. Şebnem Ön; S. Acar; K. Demir; A. Abacı; Y. Öztürk; S. K. Çelik; E. Böber |
| Evolution from primary biliary cirrhosis to primary biliary cirrhosis/autoimmune hepatitis overlap syndrome | 2008 | W. S. Twaddell; J. Lefkowitch; P. D. Berk |
| Excess intraoperative fluid volume administration is associated with pancreatic fistula after pancreaticoduodenectomy | 2017 | I. W. Han; H. Kim; J. Heo; M. G. Oh; Y. S. Choi; S. E. Lee; C. S. Lim |
| Exhaled volatile organic compounds for cholangiocarcinoma diagnosis | 2022 | N. Siriwong; T. Sukaram; R. Tansawat; T. Apiparakoon; T. Tiyarattanachai; S. Marukatat; R. Rerknimitr; R. Chaiteerakij |
| Experience in Pancreatoduonectomy in a Low Volume Center | 2019 | R. R. Garcia Matus; O. L. Garcia; J. H. Cortez |
| Extending the reach of the ALBI score for primary hepatic malignancies: Does ALBI work for cholangiocarcinoma? | 2020 | F. Hahn; L. Müller; C. Düber; A. Weinmann; R. Kloeckner; A. Mähringer-Kunz |
| External beam radiotherapy (EBRT) with capecitabine (CT) in advanced gallbladder carcinoma (GBC) : A randomized controlled trial | 2011 | V. Sharma; V. D. Maheshwari; V. Rathore |
| Extraction of intra-biliary hepatocellular carcinoma by endoscopic retrograde cholangiopancreatography | 2020 | C. S. Koo; K. Y. Ho; Y. H. Pang; D. Q. Huang |
| Extrahepatic biliary tract visualization using near-infrared fluorescence imaging with indocyanine green: optimization of dose and dosing time | 2021 | Q. Chen; R. Zhou; J. Weng; Y. Lai; H. Liu; J. Kuang; S. Zhang; Z. Wu; W. Wang; W. Gu |
| Extrahepatic drug transporters in liver failure: Focus on kidney and gastrointestinal tract | 2020 | M. Droździk; S. Oswald; A. Droździk |
| Extrahepatic Malignancies in Primary Biliary Cirrhosis: A Comparative Study at Two European Centers | 2015 | A. Floreani; A. Spinazzè; L. Caballeria; A. Reig; N. Cazzagon; I. Franceschet; A. Buja; P. Furlan; K. Harada; P. S. C. Leung; M. E. Gershwin; A. Pares |
| Extrahepatic portal venous obstruction with hepatic enzyme elevation resembling hepatitis in patients with cancer | 2019 | T. Y. Liao; C. C. Liaw; H. C. Hsu; C. H. Hsieh; J. W. C. Chang; Y. H. Juan |
| Extramedullary plasmacytoma involving the abdominal vessels and pancreas [6] | 2007 | R. Ali; F. Ozkalemkas; T. Ozcelik; A. Ozkan; V. Ozkocaman; H. Ozturk; M. Kurt; Y. Sadikoglu; G. Elbuken-Ozer; A. Tunali |
| Extramedullary plasmacytoma of the gallbladder diagnosed by endoscopic ultrasound fine needle aspiration (EUS-FNA) | 2015 | P. St. Romain; S. Desai; S. Bean; X. Jiang; R. A. Burbridge |
| Factors associated with failed endoscopic drainage requiring percutaneous drainage in hilar cholangiocarcinoma | 2016 | T. Kerdsirichairat; P. Perera; J. Jorgensen; M. Anderson; G. Elta; J. Scheiman; M. M. Xu; R. Kwon |
| Factors Associated With Growth After Deceased and Live Donor Pediatric Liver Transplantation | 2019 | O. M. Andacoglu; A. Himmler; X. Geng; J. Ahn; E. Conlon; K. Khan; N. Yazigi; T. M. Fishbein |
| Factors influencing postoperative morbidity, mortality, and survival after resection for hilar cholangiocarcinoma | 1996 | C. H. Su; S. H. Tsay; C. C. Wu; Y. M. Shyr; K. L. King; C. H. Lee; W. Y. Lui; T. J. Liu; F. K. Peng |
| Factors influencing the success rates of endoscopic nasobiliary drainage in treatment of obstructive jaundice | 1995 | S. C. Wen; K. H. Lai; C. F. Chang; E. M. Wang; G. H. Lo; J. S. Cheng; R. L. Huang |
| Factors predicting benefits of proton therapy in liver tumors of ≤5cm based on the hepatic toxicity | 2022 | Y. Uchinami; N. Katoh; R. Suzuki; T. Kanehira; S. Takao; H. Taguchi; K. Kobashi; I. Yokota; H. Aoyama |
| Factors predicting concurrent cholangiocarcinomas associated with hepatolithiasis | 2003 | Y. T. Kim; J. S. Byun; J. Kim; Y. H. Jang; W. J. Lee; J. K. Ryu; S. W. Kim; Y. B. Yoon; C. Y. Kim |
| Factors predicting overall response and overall survival in hepatocellular carcinoma patients undergoing balloon-occluded transcatheter arterial chemoembolization: A retrospective cohort study | 2018 | T. Hatanaka; H. Arai; M. Shibasaki; H. Tojima; D. Takizawa; M. Toyoda; H. Takayama; T. Abe; K. Sato; S. Kakizaki; M. Yamada |
| Factors predicting requirement of high-dose transdermal fentanyl in opioid switching from oral morphine or oxycodone in patients with cancer pain | 2011 | Y. Kanbayashi; T. Hosokawa; K. Okamoto; S. Fujimoto; H. Konishi; E. Otsuji; T. Yoshikawa; T. Takagi; T. Miki; M. Taniwaki |
| Factors predicting surgical resection in patients with intrahepatic cholangiocarcinoma and cirrhosis | 2014 | H. Li; J. S. Wu; X. T. Wang; P. Lv; L. S. Gong; G. Liu; B. N. Tian; Y. Y. Li; B. Jiang |
| Factors related to stent patency and early elimination of jaundice using bile duct stent combined with iodine-125 seed implantation in malignant obstructive jaundice | 2022 | L. Q. Han; N. J. Xiao; F. Liu; X. D. Wang; Z. K. Wang; W. Li |
| FAT10 promotes hepatocellular carcinoma (HCC) carcinogenesis by mediating P53 degradation and acts as a prognostic indicator of HCC | 2021 | Y. Zhang; Z. Zuo; B. Liu; P. Yang; J. Wu; L. Han; T. Han; T. Chen |
| Fatal liver gas gangrene after biliary surgery | 2017 | Y. Miyata; H. Kashiwagi; K. Koizumi; J. Kawachi; M. Kudo; S. Teshima; N. Isogai; K. Miyake; R. Shimoyama; R. Fukai; H. Ogino |
| Favorable prognostic factors of surgical outcomes in hepatocellular carcinoma patients with portal vein tumor thrombosis | 2016 | D. B. Moon; S. G. Lee; K. H. Kim; Y. J. Lee; S. Hwang; C. S. Ahn; G. W. Song; T. Y. Ha; D. H. Jung; G. C. Park |
| Feasibility and tolerability of sintilimab plus anlotinib as the second-line therapy for patients with advanced biliary tract cancers: An open-label, single-arm, phase II clinical trial | 2023 | S. Jin; R. Zhao; C. Zhou; Q. Zhong; J. Shi; C. Su; Q. Li; X. Su; H. Chi; X. Lu; G. Jiang; R. Chen; J. Han; M. Jiang; S. Qiao; J. Liu; M. Song; L. Song; Y. Du; Z. Chang; M. Wang; M. Dong; Y. Zhong; P. Yu; X. Zhang; H. Zong |
| Fecal elastase-1 testing and serum nutritional markers in biliopancreatic cancer patients | 2020 | D. Oyón; L. Zabalza; M. Rullán; G. De La Higuera B; C. Saldaña; M. Casi; E. Borobio; F. Bolado; J. Urman; D. Ruiz-Clavijo |
| Fever, jaundice, and liver failure in an 18-year-old male | 2012 | P. Tanpowpong; S. Sabharwal; U. Shah |
| Fibroblast growth factor 23-secreting pancreaticobiliary malignancy | 2018 | V. Barta; N. Lalkiya; H. Phan; I. Miller; M. Sachdeva |
| Fibrolamellar hepatocellular carcinoma with extensive vascular thrombosis | 2015 | M. Bhagat; S. Kembhavi; S. Qureshi |
| First-In-Human Effects of PPT1 Inhibition Using the Oral Treatment with GNS561/Ezurpimtrostat in Patients with Primary and Secondary Liver Cancers | 2022 | J. J. Harding; A. Awada; G. Roth; T. Decaens; P. Merle; N. Kotecki; C. Dreyer; C. Ansaldi; M. Rachid; S. Mezouar; A. Menut; E. N. Bestion; V. Paradis; P. Halfon; G. K. Abou-Alfa; E. Raymond |
| First-in-man histotripsy of hepatic tumors: the THERESA trial, a feasibility study | 2022 | J. Vidal-Jove; X. Serres; E. Vlaisavljevich; J. Cannata; A. Duryea; R. Miller; X. Merino; M. Velat; Y. Kam; R. Bolduan; J. Amaral; T. Hall; Z. Xu; F. T. Lee; T. J. Ziemlewicz |
| First-line Nab-paclitaxel and gemcitabine in patients with metastatic pancreatic cancer from routine clinical practice | 2014 | A. Vogel; U. Pelzer; A. B. Salah-Eddin; W. Köster |
| Fucosyl-agalactosyl IgG1 induces cholangiocarcinoma metastasis and early recurrence by activating tumor-associated macrophage | 2018 | T. T. Chang; H. W. Tsai; C. H. Ho |
| Functional Correlation between the Pancreas and the Small Intestine in Humans the First Evaluation Using a Newly Developed Enteroscopy | 2018 | D. Hayashi; Y. Hirooka; H. Kawashima; E. Ohno; T. Ishikawa; T. Kuwahara; M. Kawai; T. Yamamura; K. Furukawa; K. Funasaka; M. Nakamura; R. Miyahara; O. Watanabe; M. Ishigami; S. Hashimoto; H. Goto |
| Functional liver reserve parameters predictive for posthepatectomy complications | 2013 | A. Nanashima; T. Abo; J. Arai; H. Matsumoto; T. Kudo; T. Nagayasu |
| Gall bladder cancer in a child: A rare occurrence | 2012 | D. K. Muduly; S. S. Deo; N. K. Shukla; A. A. Kallianpur; R. Prakash; T. Jayakrishnan |
| Gallbladder adenoma in a domestic shorthair cat | 2021 | C. Broadbridge; S. S. Taylor; H. Renfrew; F. Gemignani; V. Livet; T. Vicek; M. Dobromylskyj |
| Gallbladder metastasis of renal clear cell carcinoma 15 years after primary cancer excision: A case report | 2018 | Y. Saito; H. Okuda; M. Yoshida; S. Okimasa; T. Fukuda; M. Yano; M. Ochi; Y. Okamoto; H. Nakayama; E. Ono; H. Ohdan |
| Gallbladder metastasis secondary to gastric cancer as a first site of recurrence presented with acute cholecystitis: Case report and literature review | 2012 | A. Bilici; M. Seker; B. B. Öven Ustaalioglu; S. Hallaç Keser; F. Cinaral; M. Gümüş |
| Gamma glutamyl transferase ‘To be or not to be’ a liver function test? | 2016 | J. F. Dillon; M. H. Miller |
| Gangliocytic paraganglioma of the common bile duct | 2008 | M. Muthuswamy; Z. Zhang; E. Stueben |
| Gastrectomy history as a predictor of post-hepatectomy complications | 2022 | N. Harimoto; K. Araki; K. Hagiwara; T. Yamanaka; N. Ishii; M. Tsukagoshi; T. Igarashi; A. Watanabe; N. Kubo; K. Shirabe |
| Gastric outlet obstruction as an unusual presentation for metastatic lobular breast cancer | 2019 | H. Swerdloff; J. Duchesne; J. B. Hamner; E. Elliott; A. Smith; M. Moe; A. Stolier; P. Daroca; A. Sholl; A. Souers |
| Gastrointestinal Tuberculosis (TB): A Great Mimicker! | 2011 | T. Zolfaghari; G. Goel; I. Donepudi; H. Hertan |
| Gemcitabine and Cisplatin as Neo-Adjuvant for Cholangiocarcinoma Patients Prior to Liver Transplantation: Case-Series | 2022 | M. Abdelrahim; H. Al-Rawi; A. Esmail; J. Xu; G. Umoru; F. Ibnshamsah; A. Abudayyeh; D. Victor; A. Saharia; R. McMillan; E. Al Najjar; D. Bugazia; M. Al-Rawi; R. M. Ghobrial |
| Gemcitabine-induced hemolytic uremic syndrome mimicking scleroderma renal crisis presenting with Raynaud's phenomenon, positive antinuclear antibodies and hypertensive emergency | 2014 | Y. Yamada; K. Suzuki; H. Nobata; H. Kawai; R. Wakamatsu; N. Miura; S. Banno; H. Imai |
| General condition, including serum albumin level, is considered to decide performance of ERCP for patients with unresectable pancreatic head cancer | 2015 | S. Onoue; T. Matsuura; T. Yamada |
| General management of nonagenarian patients: A review of the literature | 2014 | R. Rivoirard; C. Chargari; J. C. Tronec; A. T. Falk; J. B. Guy; H. Eddekkaoui; R. Lahmar; C. Pacaut; B. Mery; A. Assouline; P. Auberdiac; G. Moriceau; R. Gonthier; A. Guillota; Y. Merrouche; N. Magne |
| Genetic interaction of hepatocyte nuclear factor 6 and notch signaling within the liver | 2010 | C. Vanderpool; E. Sparks; K. A. Huppert; S. S. Huppert |
| Giant duodenal diverticulum with mucinous carcinoma of distal bile duct, mimmicking Lemmel syndrome: A rare case report | 2022 | B. P. Budiono; M. A. Chionardes; S. A. Prasetyo; I. Riwanto |
| Glasgow Prognostic Score predicts poor prognosis among advanced biliary tract cancer patients with good performance status | 2014 | T. Moriwaki; K. Ishige; M. Araki; S. Yoshida; M. Nishi; M. Sato; T. Yamada; Y. Yamamoto; M. Ozeki; H. Ishida; T. Yamaguchi; K. Matsuda; T. Murashita; M. Abei; I. Hyodo |
| Glasgow Prognostic Score predicts prognosis of intrahepatic cholangiocarcinoma | 2017 | Q. X. Pan; Z. J. Su; J. H. Zhang; C. R. Wang; S. Y. Ke |
| Growth and nutritional evaluation in children with chronic liver disease (CLD) before and after liver transplantation | 2010 | B. Predieri; A. R. Di Biase; R. Berri; P. Bruzzi; A. Colecchia; L. Iughetti |
| Growth hormone resistance and somatomedins in children with end-stage liver disease awaiting transplantation | 1998 | R. M. Greer; P. Quirk; G. J. Cleghorn; R. W. Shepherd |
| Gut-liver relationship: Frequently a forgotten feature in clinical practice | 2004 | A. Federico; C. Tuccillo; F. Terracciano; M. V. D'Auria; C. De Simone; C. D. Blanco; C. Loguercio |
| Hemobilia requiring arterial embolization as an immediate acute complication of Radiofrequency Ablation Therapy (RFA) for hepatocellular carcinoma (HCC): A case report and literature review | 2010 | F. Rzouq; H. Hatoum |
| Hemoglobin and Neutrophil Count as Prognostic Factors in Cholangiocarcinoma Patients in 2nd Line Treatment Setting: Results from a Small Monocentric Retrospective Study | 2023 | C. Liguori; C. Copparoni; C. Felicetti; F. Pecci; A. Lupi; G. Pinterpe; R. Berardi; R. Giampieri |
| Hepatectomy versus hepatectomy with lymphadenectomy in hepatocellular carcinoma | 2015 | X. Wu; B. Li; J. Qiu; J. Shen; Y. Zheng; Q. Li; Y. Liao; W. He; R. Zou; Y. Yuan |
| Hepatic Abscess following Yttrium-90 Radioembolization in Patients with Surgical Bilioenteric Anastomosis or Compromised Sphincter of Oddi: A Tertiary Cancer Center Experience | 2022 | K. N. Agahi; A. Mahvash; M. E. Abdelsalam |
| Hepatic Arterial Infusion Therapy in Advanced Cancer and Liver-Predominant Disease: The MD Anderson Experience | 2013 | A. M. Tsimberidou; C. Vaklavas; S. Fu; S. Wen; J. A. Lim; D. Hong; J. Wheler; A. Naing; C. Uehara; M. Wallace; R. Kurzrock |
| Hepatic autotaxin overexpression in infants with biliary atresia | 2018 | W. Udomsinprasert; P. Vejchapipat; N. Klaikeaw; V. Chongsrisawat; Y. Poovorawan; S. Honsawek |
| Hepatic dysfunction and MELD score improve after orthotopic heart transplantation and predict post-transplantation mortality | 2010 | A. Chokshi; F. Cheema; K. Schaefle; T. Khawaja; M. Farr; H. Takayama; Y. Naka; D. Mancini; P. Christian Schulze |
| Hepatic Extraction Fraction: Interest of its Quantification in Patients with Hepatic Tumours | 2012 | J. G. Tralhao; A. M. Abrantes; E. Hoti; D. Cardoso; R. Figueirinha; M. F. Botelho; F. Castro-Sousa |
| Hepatic functional reserve in patients with biliary malignancies: an assessment by technetium 99m galactosyl human serum albumin hepatic scintigraphy | 1999 | K. Fukunaga; T. Todoroki; Y. Takada; M. Otsuka; T. Kawamoto; K. Fukao |
| Hepatic functional reserve in patients with obstructive jaundice: An assessment by the redox tolerance test | 1995 | T. Tsubono; K. Tsukada; K. Hatakeyama |
| Hepatic hemangioma as a rare cause of bilious ascites | 2017 | N. Orekondy; V. Desai; J. Mitchell |
| Hepatic insufficiency and nutritional problems after major hepatobiliary surgery | 1984 | B. Launois; D. Grossetti; E. Marcade |
| Hepatic perihilar amphicrine cholangiocarcinoma: A case report | 2018 | T. W. Czeczok; D. J. Schembri-Wismayer; T. C. Smyrk; M. J. Truty; T. Mounajjed |
| Hepatic resection for hepatocellular carcinoma with obstructive jaundice due to biliary tumor thrombi | 2004 | C. N. Yeh; Y. Y. Jan; W. C. Lee; M. F. Chen |
| Hepatic tuberculosis in an immunocompetent patient-a diagnostic challenge | 2016 | A. Nawaz; K. Malik; A. Alvi; B. Ali |
| Hepatitis C virus infection as a likely etiology of intrahepatic cholangiocarcinoma | 2004 | S. Yamamoto; S. Kubo; S. Hai; T. Uenishi; T. Yamamoto; T. Shuto; S. Takemura; H. Tanaka; O. Yamazaki; K. Hirohashi; T. Tanaka |
| Hepatobiliary and pancreatic: A woman with jaundice and anorexia | 2001 | N. Mikami; K. Okuda |
| Hepatobiliary and pancreatic: An old woman with stones and jaundice | 2002 | N. Mikami; K. Okuda; I. C. Roberts-Thomson |
| Hepatobiliary cancers | 2009 | A. B. Benson Iii; T. A. Abrams; E. Ben-Josef; P. M. Bloomston; J. F. Botha; B. M. Clary; A. Covey; S. A. Curley; M. I. D'Angelica; R. Davila; W. D. Ensminger; J. F. Gibbs; D. Laheru; M. P. Malafa; J. Marrero; S. G. Meranze; S. J. Mulvihill; J. O. Park; J. A. Posey; J. Sachdev; R. Salem; E. R. Sigurdson; C. Sofocleous; J. N. Vauthey; A. P. Venook; L. W. Goff; Y. Yen; A. X. Zhu |
| Hepatobiliary phase images using gadolinium-ethoxybenzyl-diethylenetriamine penta-acetic acid-enhanced MRI as an imaging surrogate for the albumin-bilirubin grading system | 2016 | Y. Takatsu; S. Kobayashi; T. Miyati; T. Shiozaki |
| Hepatobiliary Quiz Answers – 18 (2016) | 2016 | S. Rathi; R. K. Dhiman |
| Hepatocarcinogenesis in multidrug-resistant P-glycoprotein 3 deficiency | 2017 | M. Vij; N. P. Shanmugam; M. S. Reddy; S. Govil; M. Rela |
| Hepatocellular carcinoma as predominant cancer subgroup accounting for sex differences in post-hepatectomy liver failure, morbidity and mortality | 2022 | G. De la Cruz Ku; M. Aizpuru; H. Hackl; D. S. Ubl; E. B. Habermann; R. Pery; M. Driedger; A. Assinger; D. M. Nagorney; S. P. Cleary; R. L. Smoot; P. Starlinger |
| Hepatocellular carcinoma complicating biliary cirrhosis caused by biliary atresia: Report of a case | 1995 | M. Kohno; H. Kitatani; H. Wada; T. Kajimoto; H. Matuno; M. Tanino; T. Nakagawa; A. Takarada |
| Hepatocellular Carcinoma Risk, Outcomes, and Screening After Hepatitis C Eradication | 2021 | R. D’Ambrosio; G. N. Ioannou |
| Hepatocellular carcinoma secondary to cholecystectomy: A one in a million chance | 2002 | R. A. E. Clayton; V. Bettschart; R. W. Parks; O. J. Garden |
| Hepatocellular carcinoma with steatohepatitic features: A clinicopathological study of Japanese patients | 2014 | J. Shibahara; S. Ando; Y. Sakamoto; N. Kokudo; M. Fukayama |
| Hepatocellular carcinoma: Epidemiology, screening, and assessment of hepatic reserve | 2020 | S. Z. Frager; J. M. Schwartz |
| Hepatocellular uptake index obtained with gadoxetate disodium-enhanced magnetic resonance imaging in the assessment future liver remnant function after major hepatectomy for biliary malignancy | 2021 | T. Notake; A. Shimizu; K. Kubota; T. Ikehara; H. Hayashi; K. Yasukawa; A. Kobayashi; A. Yamada; Y. Fujinaga; Y. Soejima |
| Hepatogastric fistula as a rare complication of pyogenic liver abscess | 2017 | K. W. Lee; H. Y. Kim; C. W. Kim; Y. K. Kim; O. Kwon; M. A. Kim; Y. Cho; K. Yang |
| Hepatoprotective effect of the hydroalcoholic extract of Cichorium intybus in a rat model of obstructive cholestasis | 2021 | M. R. Moloudi; K. Hassanzadeh; M. Abdi; F. Zandi; K. Rahimi; E. Izadpanah |
| HHLA2 in intrahepatic cholangiocarcinoma: An immune checkpoint with prognostic significance and wider expression compared with PD-L1 | 2019 | C. Y. Jing; Y. P. Fu; Y. Yi; M. X. Zhang; S. S. Zheng; J. L. Huang; W. Gan; X. Xu; J. J. Lin; J. Zhang; S. J. Qiu; B. H. Zhang |
| High mobility group box 1 release from cholangiocytes in patients with acute-on-chronic liver failure | 2014 | H. Xu; H. Li; Y. Qu; J. Zheng; J. Lu |
| High Prevalence and Conservative Management of Acute Cholecystitis during Lenvatinib for Advanced Thyroid Cancer | 2021 | M. Di Stefano; C. Colombo; S. De Leo; M. Perrino; M. Viganò; L. Persani; L. Fugazzola |
| HIGH SERUM LEVELS OF ALKALINE-PHOSPHATASE ARE ASSOCIATED WITH CANCER IN CHOLESTATIC JAUNDICE | 1985 | J. W. Wilson; I. J. Kronborg; I. C. Robertsthomson |
| Histoplasmosis presenting as new-onset ascites in a crohn's disease patient on adalimumab | 2021 | H. Hall; L. Stefaniwsky; K. De Felice; W. Bivin |
| Hodgkin's disease presenting with chylous ascites and cavernous transformation of the portal vein | 2007 | C. Gonen; M. Akarsu; D. Solmaz; T. Yilmaz; M. Secil; F. Obuz; A. Kargi |
| Hyaluronic acid and TGF-β1 in dogs with hepatobiliary diseases | 2018 | V. Ceplecha; K. Řeháková; C. Lendon; P. Proks; M. Škorič; G. C. M. Grinwis; B. Hřibová; M. Vávra; J. Lorenzová; M. Crha |
| Hybrid extracorporeal therapies as a bridge to pediatric liver transplantation | 2018 | A. A. Arikan; P. Srivaths; R. W. Himes; N. T. Pekkucuksen; F. Lam; T. Nguyen; T. Miloh; M. Braun; J. Goss; M. S. Desai |
| Hypercalcemia related to cholangiocellular carcinoma | 2019 | E. Onalan |
| Hyperthermia associated with biliary obstruction during living donor liver transplantation | 2018 | H. Kang; J. Park; J. J. Lee; G. S. Kim |
| Iatrogenic portobiliary fistula complicating endoscopic stenting of a pancreatic cyst, with possible carcinoma: A case report | 2011 | W. D. Stableforth; S. Pathmakanthan; A. Holt; G. Haydon; D. Freshwater; K. Mangat |
| ICG immunofluorescence guided pure laparoscopic right hepatectomy for HCC in patient with cirrhosis | 2016 | T. T. Cheung |
| Identification of Remaining Life Expectancy Less Than Two Weeks by C-Reactive Protein/Albumin Ratio, Prognostic Nutritional Index, Fibrosis-4 Index, and Albumin-Bilirubin Score in Terminal Cancer Patients | 2022 | S. Ieda; T. Miyamoto; K. Hosomi; M. Takegami; A. Kawabata |
| IgG4 cholangiopathy masquerading as a Klatskin tumor: An interesting case | 2021 | B. Varda; S. Aslam; Z. Ansari; K. Kadkhodayan; M. Patel |
| IgG4-Related Disease: A Constellation of Abdominal Manifestations | 2022 | K. Wattamwar; J. Holder |
| Immediate and long-term outcomes of hepatectomy for hepatolithiasis | 2004 | D. W. Chen; R. Tung-Ping Poon; C. L. Liu; S. T. Fan; J. Wong |
| Immunoglobulin (IgG4) Sclerosing Cholecystitis-Camouflaging Gall Bladder Cancer—a Case Report and Review of Literature | 2023 | R. Singh; P. Mahajan; R. Prasher; V. Rajdev; J. Singh; C. M. Kattoor; K. Mardi; J. Gupta |
| Immunoglobulin G4-related disease mimicking gallbladder cancer with associated choledochal cyst: A case report of a malignant masquerade | 2019 | A. A. Kulkarni; P. Soni; V. K. Sharma; A. Bal; S. S. Rana; R. Gupta |
| Immunoglobulin G4-related sclerosing cholangitis | 2015 | D. Joshi; G. J. Webster |
| Immunotherapy-related hepatitis: Real-world experience from a tertiary centre | 2019 | V. Cheung; T. Gupta; M. Payne; M. R. Middleton; J. D. Collier; A. Simmons; P. Klenerman; O. Brain; J. F. Cobbold |
| Impact of hepatitis C virus on survival in patients undergoing resection of intrahepatic cholangiocarcinoma: Report of a Japanese nationwide survey | 2021 | M. Kaibori; K. Yoshii; K. Kashiwabara; T. Kokudo; K. Hasegawa; N. Izumi; T. Murakami; M. Kudo; S. Shiina; M. Sakamoto; O. Nakashima; Y. Matsuyama; S. Eguchi; T. Yamashita; T. Takayama; N. Kokudo; S. Kubo |
| Impact of JMJD6 on intrahepatic cholangiocarcinoma | 2022 | Y. Kosai-Fujimoto; S. Itoh; K. Yugawa; T. Fukuhara; D. Okuzaki; T. Toshima; N. Harada; Y. Oda; T. Yoshizumi; M. Mori |
| Impact of nucleos(t)ide analog treatment on the development of malignancy in patients with chronic hepatitis B | 2018 | S. S. Kim; E. K. Ahn; S. Y. Cho; R. W. Park; H. J. Cho; J. H. Kim; H. G. Kim; G. R. Lee; S. H. Hwang; M. J. Yang; J. Y. Cheong; S. W. Cho |
| Impact of perioperative blood transfusion on long-term survival in patients with different stages of perihilar cholangiocarcinoma treated with curative resection: A multicentre propensity score matching study | 2022 | Z. P. Liu; Z. J. Cheng; H. S. Dai; S. Y. Zhong; D. C. Zhao; Y. Gong; J. H. Zuo; X. Y. Che; W. Y. Chen; Z. R. Wang; T. Yu; J. J. Cheng; X. C. Liu; J. Bai; Y. Jiang; Y. Q. Zhang; W. Y. Lau; S. Q. Deng; Z. Y. Chen |
| Impact of post-hepatectomy liver failure on morbidity and short- and long-term survival after major hepatectomy | 2022 | R. Baumgartner; S. Gilg; B. Björnsson; K. Hasselgren; P. Ghorbani; C. Sauter; P. Stål; P. Sandstöm; E. Sparrelid; J. Engstrand |
| Impact of preoperative biliary drainage on postoperative complications and prognosis after pancreaticoduodenectomy: A single-center retrospective cohort study | 2022 | D. Wang; H. Lin; C. Guan; X. Zhang; P. Li; C. Xin; X. Yang; Z. Feng; Y. Min; X. Gu; W. Guo |
| Impact of viral aetiology in the phase III HIMALAYA study of tremelimumab (T) plus durvalumab (D) in unresectable hepatocellular carcinoma (uHCC) | 2022 | L. S. Chan; M. Kudo; B. Sangro; R. K. Kelley; J. Furuse; J. W. Park; P. Sunpaweravong; A. Fasolo; T. Yau; T. Kawaoka; A. L. Cheng; S. Azevedo; M. E. Reig Monzon; E. Assenat; M. Yarchoan; A. R. He; M. Makowsky; D. Ran; A. Negro; G. K. Abou-Alfa |
| Improved Outcomes for Liver Transplantation in Patients with Biliary Atresia Since Pediatric End-Stage Liver Disease Implementation: Analysis of the Society of Pediatric Liver Transplantation Registry | 2020 | S. A. Taylor; V. Venkat; R. Arnon; V. V. Gopalareddy; P. Rosenthal; J. Erinjeri; R. Anand; J. F. Daniel |
| Improvement of Serum Alkaline Phosphatase to Less Than 1.5 the Upper Limit of Normal is Associated With a Better Prognosis in Patients With Primary Sclerosing Cholangitis (PSC) | 2012 | S. Al Mamari; J. Djordjevic; J. S. Halliday; R. W. Chapman |
| Incidence and risk factors for sustained hepatic function toxicity 6 months after radioembolization: Analysis of the radiation-emitting sir-spheres in non-resectable liver tumor (RESIN) registry | 2021 | D. Brown; H. Krebs; J. Brower; R. O'Hara; E. Wang; K. Vaheesan; L. Du; L. Matsuoka; D. D'Souza; D. Y. Sze; J. Golzarian; R. Gandhi; A. Kennedy |
| Incidence, prevalence, and natural history of primary sclerosing cholangitis in the United Kingdom | 2017 | H. Liang; S. Manne; J. Shick; T. Lissoos; P. Dolin |
| Increased Serum Levels of Carbohydrate Antigen 19-9 in Primary Sclerosing Cholangitis is Associated With Reduced Survival Even in Patients Without Cholangiocarcinoma | 2012 | U. Navaneethan; P. G. K. Venkatesh; B. Lashner; F. H. Remzi; P. R. Kiran; J. J. Fung |
| Increased serum mitochondrial creatine kinase activity as a risk for hepatocarcinogenesis in chronic hepatitis C patients | 2014 | K. Enooku; H. Nakagawa; Y. Soroida; R. Ohkawa; Y. Kageyama; B. Uranbileg; N. Watanabe; R. Tateishi; H. Yoshida; K. Koike; Y. Yatomi; H. Ikeda |
| Increasing Yttrium-90 Dose Conformality Using Proximal Radioembolization Enabled by Distal Angiosomal Truncation for the Treatment of Hepatic Malignancy | 2020 | J. M. Core; G. T. Frey; A. Sharma; S. T. Bussone; J. D. Legout; J. M. McKinney; A. R. Lewis; C. Ritchie; Z. Devcic; R. Paz-Fumagalli; B. B. Toskich |
| Independent factors predicting postoperative 30-day mortality in 101 infants following liver transplantation | 2017 | C. W. Cho; S. Lee; J. M. Kim; G. S. Choi; C. H. D. Kwon; J. W. Joh; S. K. Lee |
| Index of convexity: A novel liver function index using Tc-GSA scintigraphy | 2013 | K. Miki; Y. Matsui; M. Teruya; M. Kaminishi; N. Kokudo |
| Indocyanine green fluorescence-guided laparoscopic hepatectomy versus conventional laparoscopic hepatectomy for hepatocellular carcinoma: A single-center propensity score matching study | 2022 | W. Jianxi; Z. Xiongfeng; Z. Zehao; Z. Zhen; P. Tianyi; L. Ye; J. Haosheng; J. Zhixiang; W. Huiling |
| Indocyanine green plasma clearance rate and 99mTc-galactosyl human serum albumin single-photon emission computed tomography evaluated preoperative remnant liver | 2022 | K. Iwaki; S. Kaihara; R. Kita; K. Kitamura; H. Hashida; K. Uryuhara |
| Inflammation-based prognostic score predicts biliary stent patency in patients with unresectable malignant biliary obstruction | 2014 | Y. Iwasaki; M. Ishizuka; M. Kato; J. Kita; M. Shimoda; K. Kubota |
| Inflammation-based prognostic scores in patients with extrahepatic bile duct lesions treated by percutaneous transhepatic biliary stenting combined with 125I seeds intracavitary irradiation | 2019 | X. Hu; Q. Pang; H. Liu; Z. Qian; H. Jin; L. Zhou; Y. Wang; Z. Man; Z. Li; S. Yang |
| Influence of cirrhosis on outcomes of patients with advanced intrahepatic cholangiocarcinoma receiving chemotherapy | 2022 | C. D'Abrigeon; M. G. McNamara; S. Le Sourd; A. Lamarca; A. Lièvre; R. A. Hubner; J. W. Valle; J. Edeline |
| Influence of halloysite nanotubes on the efficiency of Asparaginase against mice Ehrlich solid carcinoma | 2022 | B. M. M. Baharoon; A. M. Shaik; S. M. El-Hamidy; R. E. El-Araby; A. H. Batawi; M. A. Salam |
| Influence of histological inflammatory activity on regenerative capacity of liver after percutaneous transhepatic portal vein embolization | 1999 | H. Tanaka; K. Hirohashi; S. Kubo; T. Ikebe; T. Tsukamoto; H. Hamba; T. Shuto; K. Wakasa; H. Kinoshita |
| Influence of the water jet system vs cavitron ultrasonic surgical aspirator for liver resection on the remnant liver | 2022 | T. Hanaki; A. Tsuda; T. Sunaguchi; K. Goto; M. Morimoto; Y. Murakami; K. Kihara; T. Matsunaga; M. Yamamoto; N. Tokuyasu; T. Sakamoto; T. Hasegawa; Y. Fujiwara |
| Infrahepatic Inferior Vena Cava Clamping Reduces Blood Loss during Liver Transection for Cholangiocarcinoma | 2021 | N. Leeratanakachorn; V. Luvira; T. Tipwaratorn; S. Theeragul; A. Jarearnrat; A. Titapun; T. Srisuk; S. Kamsa-Ard; A. Pugkhem; N. Khuntikeo; C. Pairojkul; V. Bhudhisawasdi |
| Initial experience of drug-eluting bead-transcatheter arterial chemoembolization after lipiodol-based transcatheter arterial chemoembolization failure for patients with advanced hepatocellular carcinoma | 2021 | K. C. Liu; W. F. Lv; D. Lu; C. L. Hou; J. Xie; Y. H. Lu; Q. S. Cao; Y. L. Tan; Y. Z. Zhang; J. Liu |
| Integrating Machine Learning and Tumor Immune Signature to Predict Oncologic Outcomes in Resected Biliary Tract Cancer | 2021 | G.-W. Ji; K. Wang; Y.-X. Xia; J.-S. Wang; X.-H. Wang; X.-C. Li |
| Integration of chemoembolization and radioembolization into multimodal treatment of cholangiocarcinoma | 2015 | R. Seidensticker; J. Ricke; M. Seidensticker |
| Internal radioembolization for colorectal carcinoma liver metastases | 2009 | S. M. Ibrahim; A. Riaz; R. J. Lewandowski; M. F. Mulcahy; A. B. Benson Iii; R. Salem |
| Interventional transhepatic biliary drainage for obstructive jaundice caused by hilar cholangiocarcinoma: An analysis of related factors influencing the prognosis | 2012 | C. Xu; H. B. Shi; S. Liu; Z. Q. Yang; W. Z. Zhou; L. S. Li |
| Intraarterial 5-fluorouracil and interferon therapy is safe and effective for nonresectable biliary tract adenocarcinoma | 2015 | Y. Yashima; S. Sato; T. Kawai; T. Sugimoto; T. Sato; M. Kanda; S. Obi |
| Intraductal Hepatocellular Carcinoma Without Evidence of Hepatic Parenchymal Involvement | 2017 | S. Levy; J. P. Norvell |
| Intraductal mucosal-spreading mucin-producing peripheral cholangiocarcinoma of the liver | 2000 | J. H. Lim; Y. I. Kim; C. K. Park |
| Intrahepatic biliary cysts presenting before hepatic portoenterostomy in biliary atresia | 2006 | E. Mas; F. Alvarez; L. L. Oligny; S. Martin |
| Intrahepatic Cholangiocarcinoma Treated with Transarterial Yttrium-90 Glass Microsphere Radioembolization: Results of a Single Institution Retrospective Study | 2018 | A. Gangi; J. Shah; N. Hatfield; J. Smith; J. Sweeney; J. Choi; G. El-Haddad; B. Biebel; N. Parikh; B. Arslan; S. E. Hoffe; J. M. Frakes; G. M. Springett; D. A. Anaya; M. Malafa; D.-T. Chen; Y. Chen; R. D. Kim; R. Shridhar; B. Kis |
| Intrahepatic cholangiocarcinoma with portal vein tumor thrombus and elevated level of α-fetoprotein: A case report | 2008 | T. Kato; S. Katagiri; S. Ariizumi; Y. Kotera; Y. Takahashi; K. Imai; T. Noguchi; M. Yamamoto |
| Intrahepatic multicystic biliary hamartoma: A case report | 2022 | C. Y. Wang; F. Y. Shi; W. F. Huang; Y. Tang; T. Li; G. L. He |
| Intraoperative blood requirements during living donor liver transplantation in pediatric recipients | 2013 | D. Sachan; N. Shanmugam; K. Kaliamoorthy; V. Srinivasan; J. Venkataraman; M. Rela |
| Intraoperative Positive Pancreatic Parenchymal Resection Margin: Is It a True Indication of Completion Total Pancreatectomy after Partial Pancreatectomy for Pancreatic Ductal Adenocarcinoma? | 2022 | J. H. Jung; S. J. Yoon; O. J. Lee; S. H. Shin; J. S. Heo; I. W. Han |
| Irinotecan-induced NASH and liver failure | 2021 | M. Araz; F. Kilinc; U. Kerimoglu; M. Keskin; T. Kucukkartallar |
| Irreversible Electroporation Treatment With Intraoperative Biliary Stenting for Unresectable Perihilar Cholangiocarcinoma: A Pilot Study | 2021 | P. C. Yang; Y. J. Chen; X. Y. Li; C. Y. Hsiao; B. B. Cheng; Y. Gao; B. Z. Zhou; S. Y. Chen; S. Q. Hu; Q. Zeng; K. W. Huang |
| Is recurrent pyogenic cholangitis an independent poor prognostic indicator for resectable intrahepatic cholangiocarcinoma? | 2019 | Y. M. Zhang; Z. T. Zhou; G. M. Liu |
| Is the Patient a Candidate for Liver Transplantation? | 2012 | A. N. Fox; R. S. Brown |
| Ischemic preconditioning provides no additive clinical value in liver resection of cirrhotic and non-cirrhotic patients under portal triad clamping: A prospective randomized controlled trial | 2014 | B. Ye; H. Zhao; H. Hou; G. Wang; F. Liu; Y. Zhao; Z. Zhang; K. Xie; L. Zhu; X. Geng |
| Isolated IgG4-related sclerosing cholangitis with normal serum IgG4 levels—A case report | 2020 | Y. Shu; J. Cheng; J. Ye; X. Pan |
| Isolated intrahepatic changes on cholangiography at the time of Primary Sclerosing Cholangitis diagnosis is associated with a better long term survival and reduced risk of cholangiocarcinoma | 2012 | S. Al Mamari; B. Braden; J. Halliday; R. W. Chapman |
| Issues to be considered to address the future liver remnant prior to major hepatectomy | 2021 | Y. Kishi; J. N. Vauthey |
| Jack in the Duct: A case of common biliary duct schwannoma | 2019 | K. M. Kolhe; A. Amarapurkar; M. Ingle; V. Pandey; M. Amonkar; H. Khairnar; S. G. Chauhan; S. Walke; A. Shukla |
| Jaundice | 2017 | C. Novo; F. Welsh |
| Jaundice as a Diagnostic and Therapeutic Problem: A General Practitioner's Approach | 2022 | A. P. Markovic; M. Stojkovic Lalosevic; D. D. Mijac; T. Milovanovic; S. Dragasevic; A. Sokic Milutinovic; M. N. Krstic |
| Laboratory diagnostics of chronic liver diseases | 2005 | Z. Šumarac; V. Dopsaj |
| Langerhans Cell Histiocytosis Manifests with Acute Severe Hypernatremia during Hospitalization | 2022 | K. Takkavatakarn; H. Poparn; P. Katavetin |
| Langerhans'-cell histiocytosis with thoracic involvement in infant and young child: CT findings | 2017 | S. L. Shih; K. Tsai; W. Huang; F. S. Yang |
| Laparoscopic hepatopancreaticoduodenectomy for synchronous gallbladder cancer and extrahepatic cholangiocarcinoma: a case report | 2022 | G. L. Yao |
| Laparoscopic pancreaticoduodenectomy: A retrospective study of 200 cases and the optimization of the single-center learning curve | 2021 | Y. C. Tang; Q. Q. Liu; Y. G. He; J. Li; X. B. Huang |
| Laparoscopic Revision of Benign Hepaticojejunostomy Stricture Following Previous Open Pancreaticoduodenectomy | 2022 | R. K. Thota; S. Gadiyaram |
| Laparoscopic surgery for oncologic extended resection of T1b and T2 incidental gallbladder carcinoma at a high-volume center: a single-center experience in China | 2021 | P. Regmi; H. J. Hu; Y. Chang-Hao; F. Liu; W. J. Ma; C. D. Ran; J. K. Wang; A. Paudyal; N. S. Cheng; F. Y. Li |
| Laparoscopic versus open hepatectomy for intrahepatic cholangiocarcinoma in patients aged 60 and older: a retrospective cohort study | 2022 | J. Wang; W. Wang; X. Chen; D. Ma; G. Du; T. Xia; Z. Jiang; B. Jin |
| Large intrahepatic cholangiocarcinoma with tumor infiltrative lymphocytes and autoimmune hepatitis-like features | 2010 | S. Izumi; S. Nakamura; S. Mano |
| Leakage and stenosis of the hepaticojejunostomy following surgery for perihilar cholangiocarcinoma | 2020 | J. Bednarsch; Z. Czigany; D. Heise; S. A. Lang; S. W. M. Olde Damink; T. Luedde; P. Bruners; T. F. Ulmer; U. P. Neumann |
| Lemmel syndrome: an extraordinary cause of obstructive jaundice - a case report | 2022 | L. M. Goroztieta-Rosales; J. Gómez-Farías; K. D. López-García; D. O. Davila-Rodriguez |
| Lenvatinib Beyond First-Line Therapy in Patients With Advanced Biliary Tract Carcinoma | 2022 | Y. Wang; X. Yang; D. Wang; X. Yang; Y. Wang; J. Long; J. Zhou; Z. Lu; Y. Mao; X. Sang; M. Guan; H. Zhao |
| Lenvatinib-induced tumor lysis syndrome in a patient with advanced hepatocellular carcinoma: a case report | 2021 | Y. Shimizu; H. Sunagozaka; K. Yamagata; H. Hirai; M. Miura; Y. Yonemoto; Y. Naito; K. Hasatani; J. Yoshikawa; H. Aoyagi; S. Kaneko |
| Lethargy and jaundice in the context of erythropoietic protoporphyria | 2020 | E. Zhang; N. Sandanayake |
| Leveraging Blood-Based Diagnostics to Predict Tumor Biology and Extend the Application and Personalization of Radiotherapy in Liver Cancers | 2022 | F. Hauth; H. J. Roberts; T. S. Hong; D. G. Duda |
| Liver abscesses in liver transplant recipients | 2011 | I. Justo; N. Fakih; O. Caso; S. Olivares; A. Garcia; J. Calvo; A. Manrique; M. Abradelo; F. Cambra; E. Moreno; C. Jimenez |
| Liver cirrhosis on the colonic anastomotic healing in rats | 2011 | M. di Bonifacio; R. S. Parra; A. L. Normanha Ribeiro de Almeida; J. J. Ribeiro da Rocha; O. Feres |
| Liver decompensation during ombitasvir-paritaprevir/ritonavirdasabuvir and ribavirin regimen in HCV infected patients with Child-Pugh A cirrhosis | 2016 | C. Popescu; C. Dragomirescu; A. Leuştean; C. Murariu; L. Stratan; A. Badea; R. CatanǍ; A. Orfanu; R. M. NǍstase; V. Molagic; D. Munteanu; C. Tilişcan; V. AramǍ |
| Liver disease and HPLC quantification of disialotransferrin for heavy alcohol use: A case series | 2010 | S. H. Stewart; S. Comte-Walters; E. Bowen; R. F. Anton |
| Liver Disease and Risk of Hepatocellular Carcinoma in Children With Mutations in TALDO1 | 2022 | T. Grammatikopoulos; N. Hadzic; P. Foskett; S. Strautnieks; M. Samyn; R. Vara; A. Dhawan; J. Hertecant; F. Al Jasmi; O. Rahman; M. Deheragoda; L. N. Bull; R. J. Thompson |
| Liver disorder and its clinical investigation | 2015 | S. E. Haque |
| Liver Failure in patients treated with chemotherapy for colorectal liver metastases: Role of chronic disease scores in patients undergoing major liver surgery. A case-matched analysis | 2014 | F. Ratti; F. Cipriani; M. Catena; M. Paganelli; L. Aldrighetti |
| Liver functional volumetry by Tc-99m mebrofenin hepatobiliary scintigraphy before major liver resection: A game changer | 2018 | M. Gupta; P. S. Choudhury; S. Singh; D. Hazarika |
| Liver mass in a tufted capuchin (Cebus apella) | 2018 | C. Thurman; M. Klinger |
| Liver Mass in a Young Male With Ollier Disease | 2021 | R. Govalan; M. Guindi; J. D. Yang |
| Liver regeneration after anatomical resections | 2001 | A. Szubert; L. Zajac; M. Walski; M. Faryna; Z. Biejat; J. Polański |
| Liver regeneration after major liver hepatectomy: Impact of body mass index | 2016 | N. Amini; G. A. Margonis; S. Buttner; S. Besharati; Y. Kim; F. Gani; F. Sobhani; I. R. Kamel; T. M. Pawlik |
| Liver resections: Complications and survival outcome | 2007 | G. L. Grazi |
| Liver scoring system and hepatectomy outcomes: a veterans affairs corporate data warehouse analysis | 2021 | F. Maegawa; Y. Ashouri; T. Riall; I. Konstantinidis |
| Liver stiffness assessed by transient elastography is associated with parameters of liver disease and portal hypertension in 535 cholestatic children: Baseline results of the multi-center prospective force study | 2019 | B. L. Shneider; N. P. Goodrich; W. Ye; C. Sawyer; J. P. Molleston; R. Merion; D. H. Leung; S. J. Karpen; B. M. Kamath; L. Cavallo; K. S. Wang; J. H. Teckman; J. E. Squires; S. S. Sundaram; P. Rosenthal; R. Romero; K. F. Murray; K. M. Loomes; M. K. Jensen; J. A. Bezerra; L. M. Bass; R. J. Sokol; J. C. Magee |
| Liver transplantation and autoimmunity | 1999 | E. Jaeckel; H. L. Tillmann; M. P. Manns |
| Liver transplantation and whipple surgery combined with chemoradiotherapy for treatment of hilar cholangiocarcinoma in patients with primary sclerosing cholangitis | 2015 | S. Nikeghbalian; A. Shamsaeefar; A. Eshraghian; M. R. Mansoorian; K. Kazemi; B. Geramizadeh; S. A. Malek-Hosseini |
| Liver transplantation in a 7-month-old girl with Caroli's disease | 2011 | R. D. Kim; L. Book; A. Haafiz; J. J. Schwartz; J. B. Sorensen; R. P. Gonzalez-Peralta |
| Liver Tumor Markers, HALP Score, and NLR: Simple, Cost-Effective, Easily Accessible Indexes for Predicting Prognosis in ICC Patients after Surgery | 2022 | D. Zhang; H. Zeng; Y. Pan; Y. Zhao; X. Wang; J. Chen; J. Wang; Y. Zhang; Z. Zhou; L. Xu; M. Chen; D. Hu |
| Living Donor Liver Transplantation for Hepatocellular Carcinoma with Bile Duct Tumor Thrombi | 2018 | U. Uylas; H. K. Tolan; V. Ince; C. Kayaalp; S. Yilmaz |
| Locoregional Approaches in Cholangiocarcinoma Treatment | 2022 | A. E. Hare; M. S. Makary |
| Locoregional therapies in patients with recurrent intrahepatic cholangiocarcinoma after curative resection | 2020 | H. T. Liu; S. B. Cheng; C. Y. Lai; Y. J. Chen; T. C. Su; C. C. Wu |
| Long- and short-term survival following laparoscopic and open pancreaticoduodenectomy for patients with periampullary tumors in Vietnam | 2021 | T. M. Hung; T. Q. Son; T. H. Hoc; T. T. Tung; T. V. Truong; L. M. Cuong; V. D. Kien |
| Long term impact of fibrates in primary sclerosing cholangitis with incomplete biochemical response to ursodeoxycholic acid : The French-Spanish experience | 2015 | S. Lemoinne; C. Corpechot; A. D. D. Kemgang Fankem; F. Gaouar; R. Poupon; C. Housset; A. Pares; O. Chazouillères |
| Long-term Clinical Outcomes and Predictive Factors for Living-donor Liver Transplant Recipients with Biliary Strictures | 2022 | J. W. Han; J. Y. Choi; S. K. Lee; P. S. Sung; J. W. Jang; S. K. Yoon; Y. H. Choi; I. S. Lee; J. S. Oh; H. J. Chun; H. J. Choi; Y. K. You |
| Long-term effects of radium exposure in female dial workers: Liver function and liver disease | 1979 | A. P. Polednak |
| Long-term fenofibrate treatment in bile duct loss caused by drugs induced liver disease improves biochemistry | 2020 | L. Han; Y. Sun; S. Liu; Z. S. Zou; H. Xie; X. Wang |
| Long-term prognosis of combined hepatocellular and cholangiocarcinoma after curative resection comparison with hepatocellular carcinoma and cholangiocarcinoma | 2011 | J. H. Lee; G. E. Chung; S. J. Yu; S. Y. Hwang; J. S. Kim; H. Y. Kim; J. H. Yoon; H. S. Lee; N. J. Yi; K. S. Suh; K. U. Lee; J. J. Jang; Y. J. Kim |
| Loss of heat shock factor 1 promotes hepatic stellate cell activation and drives liver fibrosis | 2022 | A. Choudhury; A. Ratna; A. Lim; R. M. Sebastian; C. L. Moore; A. A. Filliol; J. Bledsoe; C. Dai; R. F. Schwabe; M. D. Shoulders; P. Mandrekar |
| Loss of microRNA-122 in mice causes hepatic inflammation and hepatocarcinogenesis | 2011 | S. H. Hsu; B. Wang; S. Costinean; S. Bai; H. Kutay; L. Yu; G. Nuovo; S. Jacob; K. Ghoshal |
| Lower incidence of biliary carcinoma in patients with primary sclerosing cholangitis and high serum levels of immunoglobulin E | 2012 | K. Hirano; M. Tada; S. Mizuno; H. Isayama; N. Takahara; R. Nagano; T. Hamada; K. Miyabayashi; Y. Ito; D. Mohri; K. Kawakubo; T. Sasaki; H. Kogure; N. Yamamoto; N. Sasahira; N. Yamashiki; Y. Sugawara; N. Kokudo; N. Toda; K. Koike |
| Mac-2-binding protein glycan isomer predicts all malignancies after sustained virological response in chronic hepatitis C | 2022 | K. Kawata; M. Atsukawa; K. Ohta; T. Chida; H. Noritake; T. Arai; K. Iwakiri; S. Yasuda; H. Toyoda; T. Okubo; A. Hiraoka; T. Watanabe; H. Uojima; A. Nozaki; J. Tani; A. Morishita; F. Kageyama; Y. Sasada; M. Nagasawa; M. Matsushita; T. Oyaizu; S. Mikami; T. Ikegami; H. Abe; K. Matsuura; Y. Tanaka; A. Tsubota |
| Machine-learning radiomics to predict early recurrence in perihilar cholangiocarcinoma after curative resection | 2021 | H. Qin; X. Hu; J. Zhang; H. Dai; Y. He; Z. Zhao; J. Yang; Z. Xu; X. Hu; Z. Chen |
| Magnetic Resonance Cholangiopancreatography Severity Predicts Disease Outcomes in Pediatric Primary Sclerosing Cholangitis: A Reliability and Validity Study | 2020 | K. Patil; A. Ricciuto; A. Alsharief; J. Al-Rayahi; A. Amirabadi; P. C. Church; B. M. Kamath; M. L. C. Greer |
| Major liver resection in elderly patients: A multi-institutional analysis | 2011 | S. K. Reddy; A. S. Barbas; R. S. Turley; T. C. Gamblin; D. A. Geller; J. W. Marsh; A. Tsung; B. M. Clary; S. Lagoo-Deenadayalan |
| Major liver resection in hilar cholangiocarcinoma: Analysis of postoperative complications | 2011 | V. Ardiles; F. Bersano; G. Moriconi; N. Resio; M. Lenz; O. Mazza; J. Pekollj; E. De Santibañes |
| Management of Hepatocellular Carcinoma | 2013 | E. H. Asham; A. Kaseb; R. M. Ghobrial |
| Marked elevation of serum alpha-fetoprotein following Clonorchis sinensis infection: A rare case report | 2022 | Y. Lin; X. Zong; M. Li; S. Wan; H. Yu; X. Wei |
| Marked Eosinophilia as the First Manifestation of Sclerosing Cholangitis | 2009 | K. Horiuchi; S. Kakizaki; T. Kosone; T. Ichikawa; K. Sato; H. Takagi; M. Mori; S. Sakurai; T. Fukusato |
| MDR3 DEFICIENCY MIMICKING WILSON DISEASE | 2021 | M. Flanagan; R. Little; I. Siddiqui; N. Jones; V. Ng |
| Mesenteric lymphangioma | 2018 | A. Aglan; M. B. Mazza |
| Meta-analysis: Ursodeoxycholic acid for primary sclerosing cholangitis | 2011 | C. K. Triantos; N. M. Koukias; V. N. Nikolopoulou; A. K. Burroughs |
| Metabolic alterations in obstructive jaundice: Effect of duration of jaundice and bile-duct decompression | 1991 | R. N. Younes; N. A. Vydelingum; P. Derooij; F. Scognamiglio; L. Andrade; M. C. Posner; M. F. Brennan |
| Metachronous intraductal papillary neoplasm of the bile duct and intraductal papillary mucinous neoplasm of the pancreas in a patient diagnosed with mucinous adenocarcinoma | 2019 | G. T. Brennan; J. G. Lee |
| Metastasis as initial presentation of squamous cell carcinoma of gallbladder: A rare clinical entity | 2017 | P. Kendre; P. Kataria; A. Patel; L. Mittal; T. Mule |
| Microbial profile and antibiotic sensitivity pattern in acute bacterial cholangitis | 2011 | M. K. Sahu; A. Chacko; A. K. Dutta; J. A. J. Prakash |
| Microwave ablation vs. surgical resection for treatment naïve hepatocellular carcinoma within the Milan criteria: a follow-up of at least 5 years | 2022 | J. Dou; Z. Cheng; Z. Han; F. Liu; Z. Wang; X. Yu; J. Yu; P. Liang |
| Minimal hepatic encephalopathy detected by brain MRI/ spectroscopy in children with chronic liver disease and/or porto-systemic shunting | 2016 | S. Hanquinet; C. Morice; L. Merlini; V. Cousin; V. McLin; M. Anooshiravani |
| Minimally invasive surgery for hilar cholangiocarcinoma: a multicenter retrospective analysis of 158 patients | 2021 | L. Jingdong; X. Yongfu; G. Yang; X. Jian; H. Xujian; L. Jianhua; Z. Wenxing; Q. Renyi; Y. Xinming; Z. Shuguo; L. Xiao; P. Bin; Z. Qifan; L. Dewei; T. Zhao-hui |
| Mixed hepatocellular cholangiocarcinoma tumors: Cholangiolocellular carcinoma is a distinct molecular entity | 2017 | A. Moeini; D. Sia; Z. Zhang; G. Camprecios; A. Stueck; H. Dong; R. Montal; L. Torrens; I. Martinez-Quetglas; M. I. Fiel; K. Hao; A. Villanueva; S. N. Thung; M. E. Schwartz; J. M. Llovet |
| Modified gemcitabine and oxaliplatin or gemcitabine + cisplatin in unresectable gallbladder cancer: Results of a phase III randomised controlled trial | 2019 | A. Sharma; B. Kalyan Mohanti; S. Pal Chaudhary; V. Sreenivas; R. Kumar Sahoo; N. Kumar Shukla; S. Thulkar; S. Pal; S. V. Deo; S. Pathy; N. Ranjan Dash; S. Kumar; S. Bhatnagar; R. Kumar; S. Mishra; P. Sahni; V. K. Iyer; V. Raina |
| Modified percutaneous transhepatic papillary balloon dilation for patients with refractory hepatolithiasis | 2020 | B. Liu; P. K. Cao; Y. Z. Wang; W. J. Wang; S. L. Tian; Y. Hertzanu; Y. L. Li |
| Molecular Adsorbent Recirculating System in Liver Transplantation: Safety and Efficacy | 2006 | R. Gaspari; A. W. Avolio; L. Zileri Dal Verme; S. Agnes; R. Proietti; M. Castagneto; A. Gasbarrini |
| Mucinous Cystic Neoplasm of the Liver and Extrahepatic Biliary Tract with Ascending Cholangitis: a Case Report and Review of the Literature | 2020 | T. Srinivas; S. P. Bhat; G. Sunder; R. Sivarajan |
| Mucobilia: An Unusual Cause of Persistent Jaundice | 2017 | J. Satiya; A. J. Lee; A. Restrepo; C. Simpfendorfer; T. Erim |
| Mucoepidermoid carcinoma of the liver: Report of a rare case and review of the literature | 2008 | Y. Arakawa; M. Shimada; T. Ikegami; T. Kubo; S. Imura; Y. Morine; H. Kanemura; H. Mori |
| Multifactorial analysis of biliary infection after percutaneous transhepatic biliary drainage treatment of malignant biliary obstruction | 2018 | H. Yu; Y. Sun; Z. Guo; W. Xing; T. Si; X. Guo; F. Liu |
| Multiple hypervascular liver nodules in a heavy drinker of alcohol | 2005 | S. R. Kim; Y. Maekawa; T. Ninomiya; S. Imoto; T. Matsuoka; K. Ando; K. Mita; K. Ku; T. Koterazawa; T. Nakajima; K. Fukuda; Y. Yano; M. Nakaji; M. Kudo; K. I. Kim; M. Hirai; Y. Hayashi |
| Multiple Myeloma Manifesting as Acute Ascites | 2017 | J. Alukal; J. Laster; A. Thomas |
| Multivariate analysis of factors associated with renal dysfunction in patients with obstructive jaundice | 2005 | F. J. Padillo; A. Cruz; J. Briceño; A. Martin-Malo; C. Pera-Madrazo; A. Sitges-Serra |
| Muscularity Defined by the Combination of Muscle Quantity and Quality is Closely Related to Both Liver Hypertrophy and Postoperative Outcomes Following Portal Vein Embolization in Cancer Patients | 2022 | S. Yao; N. Kamo; K. Taura; Y. Miyachi; S. Iwamura; M. Hirata; T. Kaido; S. Uemoto |
| Mushroom of “IM” mortality | 2011 | M. Muthuswamy; A. Gupta |
| N-acetyltransferase 2 activities in human surgical liver samples: Genetic and other disposition factors | 2010 | U. D. Kuhn; U. Settmacher; M. Hippius; A. Lupp |
| National survey of hepatobiliary and pancreatic surgery in hemophilia patients in Japan | 2022 | T. Yoshimoto-Haramura; M. Hidaka; K. Hasegawa; K. Suzumura; N. Takemura; N. Hama; T. Mizuno; T. Nomi; T. Kobayashi; K. Sano; H. Yokomizo; H. Nitta; M. Kurata; Y. Hasegawa; M. Nagayama; M. Tani; T. Fukumoto; M. Ohta; H. Hayashi; H. Taniguchi; S. Ishino; T. Aihara; T. Murase; A. Tsuchida; T. Shimamura; S. Marubashi; J. Kaneko; T. Hara; H. Matsushima; A. Soyama; T. Endo; S. Eguchi |
| Natural History and Prognostic Factors of Cholangiocarcinoma With Spinal Metastasis | 2018 | A. Sangsin; D. Saiudom; S. Pongmanee; J. Saengsin; T. Leerapun; H. Murakami |
| Natural history of small duct primary sclerosing cholangitis: a prospective and comparative study | 2005 | M. Thome; C. Guma; L. Viola; F. Alvarez |
| Neoadjuvant S-1 With Concurrent Radiotherapy Followed by Surgery for Borderline Resectable Pancreatic Cancer: A Phase II Open-label Multicenter Prospective Trial (JASPAC05) | 2022 | S. Takahashi; I. Ohno; M. Ikeda; M. Konishi; T. Kobayashi; T. Akimoto; M. Kojima; S. Morinaga; H. Toyama; Y. Shimizu; A. Miyamoto; M. Tomikawa; N. Takakura; W. Takayama; S. Hirano; T. Otsubo; M. Nagino; W. Kimura; K. Sugimachi; K. Uesaka |
| Neoadjuvant Yttrium-90 Transarterial Radioembolization with Resin Microspheres Prescribed Using the Medical Internal Radiation Dose Model for Intrahepatic Cholangiocarcinoma | 2021 | A. Sarwar; A. Ali; D. Ljuboja; J. L. Weinstein; A. S. Shenoy-Bhangle; I. A. Nasser; M. K. Morrow; S. Faintuch; M. P. Curry; A. J. Bullock; M. Ahmed |
| Neonatal Cholestasis | 2017 | E. Lane; K. F. Murray |
| Neonatal cholestasis revisited! | 2019 | J. Kaur; N. Wadhwa |
| Neonatal cholestasis: A primer of selected etiologies | 2018 | R. Ananth |
| Neural Networks for Deep Radiotherapy Dose Analysis and Prediction of Liver SBRT Outcomes | 2019 | B. Ibragimov; D. A. S. Toesca; Y. Yuan; A. C. Koong; D. T. Chang; L. Xing |
| Neutrophil to lymphocyte ratio as a prognostic marker in metastatic gallbladder cancer | 2020 | M. Mady; K. Prasai; S. H. Tella; S. Yadav; C. L. Hallemeier; S. Rakshit; L. Roberts; M. Borad; A. Mahipal |
| Neutrophil-to-lymphocyte ratio as a prognostic marker in patients with metastatic gallbladder cancer | 2019 | M. Mady; K. Prasai; S. Yadav; M. A. M. Hassan; L. R. Roberts; M. J. Borad; A. Mahipal |
| Neutrophil-to-lymphocyte ratio, platelet-to-lymphocyte ratio, and their dynamic changes during chemotherapy is useful to predict a more accurate prognosis of advanced biliary tract cancer | 2017 | K. M. Cho; H. Park; D. Y. Oh; T. Y. Kim; K. H. Lee; S. W. Han; S. A. Im; T. Y. Kim; Y. J. Bang |
| New Insights Into a Classification-Based Microvascular Invasion Prediction Model in Hepatocellular Carcinoma: A Multicenter Study | 2022 | W. Xu; Y. Wang; Z. Yang; J. Li; R. Li; F. Liu |
| New prognostic factors and scoring system for patients with skeletal metastasis | 2014 | H. Katagiri; R. Okada; T. Takagi; M. Takahashi; H. Murata; H. Harada; T. Nishimura; H. Asakura; H. Ogawa |
| Non-familial double malignancy of the colon and ampulla of vater: A case report and review of literature | 2012 | R. Rajalingam; A. Javed; R. Gondal; A. Arora; H. Nag; A. Agarwal |
| Non-functioning well-differentiated neuroendocrine tumor of the extrahepatic bile duct: An unusual suspect? | 2007 | H. Sethi; M. Madanur; P. Srinivasan; B. Portmann; N. Heaton; M. Rela |
| Nonhepatic cancer in liver cirrhosis: A retrospective study of prevalence, complication rate after specific oncological treatment, follow-up and prognostic predictors of outcome in 354 patients with cirrhosis | 2011 | F. Gundling; H. Seidl; F. Schmidtler; N. Löffler; I. Strassen; P. Wolf; C. Pehl; T. Schmidt; W. Schepp |
| Non-hodgkin's lymphoma presenting as obstructive jaundice | 2016 | S. O. Okoli; S. J. Kallus; F. G. Mirza; A. D. Seager; T. Deng; M. R. Patel |
| Nonstandard Exception Requests Impact Outcomes for Pediatric Liver Transplant Candidates | 2016 | H. J. Braun; E. R. Perito; J. L. Dodge; S. Rhee; J. P. Roberts |
| Not so Small Biliary Obstruction: A Case of Small Cell Carcinoma of the Ampulla of Vater | 2019 | P. Gomez; A. Naim; K. Zucker; C. Kiafar; A. Gomez |
| NOTCH2 variants in children with cholestatic liver disease | 2017 | T. Grammatikopoulos; S. Strautnieks; M. Sambrotta; P. Foskett; M. Deheragoda; A. Knisely; R. Thompson |
| NOTCH2 variants in cholestatic liver disease | 2015 | T. Grammatikopoulos; S. Strautnieks; M. Sambrotta; P. Foskett; M. Deheragoda; A. S. Knisely; R. J. Thompson |
| Novel prognostic model for primary sclerosing cholangitis: The importance of including laboratory values | 2015 | E. M. De Vries; J. Wang; M. Leeflang; R. Geskus; K. Boonstra; U. Beuers; C. Ponsioen |
| Novel superactive leptin antagonists and their potential therapeutic applications | 2014 | A. Gertler; E. Elinav |
| Nutritional prognostic scores in patients with hilar cholangiocarcinoma treated by percutaneous transhepatic biliary stenting combined with 125 i seed intracavitary irradiation | 2018 | P. Cui; Q. Pang; Y. Wang; Z. Qian; X. Hu; W. Wang; Z. Li; L. Zhou; Z. Man; S. Yang; H. Jin; H. Liu |
| Obstructive jaundice and bilirubin level | 1987 | H. Röding |
| Obstructive Jaundice Due to Hodgkin Lymphoma: A Rare Entity | 2012 | A. Agarwal; M. Bansal |
| Obstructive jaundice induced by biliary ascariasis | 2012 | A. Keating; J. A. Quigley; A. F. Genterola |
| OBSTRUCTIVE-JAUNDICE AND BILIRUBIN LEVEL | 1987 | H. Roding |
| Oesophageal hepatoid carcinoma with liver metastasis, a diagnostic dilemma | 2019 | A. Yahaya; W. S. Wa Kammal; N. Abd Shukor; S. S. Osman |
| Ominous yet benign double duct sign in a patient on long-term methadone maintenance | 2014 | V. Tewari; D. Tewari; R. Chigurupati; A. Rangaraju; S. Dwivedi |
| Oncological liver resection in elderly – A retrospective comparative study | 2022 | C. E. Riediger; S. Löck; L. Frohneberg; R. Hoffmann; C. Kahlert; J. Weitz |
| Operative Complications of Pancreatoduodenectomy in Patients With Elevated Serum Bilirubin | 2011 | A. E. Mukhtar; G. A. Cote; J. A. Waters; K. D. Lillemoe; C. M. Schmidt; M. G. House |
| Operative Microwave Ablation for Hepatocellular Carcinoma Within 3 cm and 3 Nodules: Experience in 559 Patients | 2022 | T. Ryu; Y. Takami; Y. Wada; H. Saitsu |
| Orthotopic Liver Transplantation for Biliary Atresia Complicated by Incidental Cholangiocarcinoma | 2012 | A. Vera; D. Villaveces; R. Lopez |
| Outcome after pancreaticoduodenectomy for periampullary cancer: An analysis from the veterans affairs national surgical quality improvement program | 2003 | K. G. Billingsley; K. Hur; W. G. Henderson; J. Daley; S. F. Khuri; R. H. Bell |
| Outcome and prognostic factors for periampullary carcinoma after pancreaticoduodenectomy: A single tertiary center experience | 2019 | P. Kositamongkol; T. Kumjornkijbovorn; C. Tovikkai; P. Mahawithitwong; W. Dumronggittigule; P. Sangserestid; S. Limsrichamrern; Y. Sirivatanauksorn |
| Outcome in 38 dogs surgically treated for hepatic abscessation | 2023 | V. Dickerson; B. Poses; P. Hyndman; J. McPhetridge; V. Scharf; B. Matz; A. Singh; J. A. Grimes |
| Outcome of minimally invasive liver resection for extrapancreatic biliary malignancies: A single-institutional experience | 2021 | K. Chin; D. Chua; S. Lee; C. Chan; B. Goh |
| Outcome of postoperative radiation therapy for cholangiocarcinoma and analysis of dose-volume histogram of remnant liver | 2019 | Y. Mukai; R. Matsuyama; I. Koike; T. Kumamoto; H. Kaizu; Y. Homma; S. Takano; Y. Sawada; M. Sugiura; Y. Yabushita; E. Ito; M. Sato; I. Endo; M. Hata |
| Outcome predictors of gemcitabine-based or fluoropyrimidine-based chemotherapy for unresectable intrahepatic cholangiocarcinoma | 2022 | C.-T. Chi; I. C. Lee; M.-H. Chen; P.-C. Lee; Y.-P. Hung; M.-C. Hou; Y. Chao; Y.-H. Huang |
| Outcomes following liver transplantation in young infants: Data from the SPLIT registry | 2021 | A. K. Jain; R. Anand; S. Lerret; G. Yanni; J. Y. Chen; S. Mohammad; M. Doyle; G. Telega; S. Horslen |
| OUTCOMES FOLLOWING PROTON BEAM THERAPY FOR HEPATOCELLULAR CARCINOMA AND INTRAHEPATIC CHOLANGIOCARCINOMA | 2020 | A. Yang; N. H. Urrunaga; O. Siddiqui; A. Wu; M. Schliep; J. Molitoris; K. Shetty; Z. Lominadze |
| Outcomes in patients receiving palliative chemotherapy for advanced biliary tract cancer | 2022 | F. Thol; S. J. Gairing; C. Czauderna; T. Thomaidis; T. Gamstatter; Y. Huber; J. Vollmar; J. Lorenz; M. Michel; F. Bartsch; L. Muller; R. Kloeckner; P. R. Galle; M.-A. Worns; J. U. Marquardt; M. Moehler; A. Weinmann; F. Foerster |
| Outcomes of hepatectomy for hepatocellular carcinoma with bile duct tumour thrombus | 2015 | T. C. L. Wong; T. T. Cheung; K. S. H. Chok; A. C. Y. Chan; W. C. Dai; S. C. Chan; R. T. P. Poon; S. T. Fan; C. M. Lo |
| Outcomes of Hepatectomy for Hepatolithiasis Based on 3-Dimensional Reconstruction Technique | 2013 | C.-h. Fang; J. Liu; Y.-f. Fan; J. Yang; N. Xiang; N. Zeng |
| Overexpressions of CK2β and XIAP are associated with poor prognosis of patients with cholangiocarcinoma | 2014 | F. Zhou; J. Xu; G. Ding; L. Cao |
| Painless jaundice: A rare presentation of primary biliary cirrhosis | 2014 | A. Kapila; P. Patel; P. Costello; M. Young |
| Palliation for nonpancreatic malignant obstruction of the biliary tract | 1990 | M. D. Finch; J. A. Butler |
| PALLIATION OF INOPERABLE BILIARY OBSTRUCTION WITH SELF-EXPANDING METAL ENDOPROSTHESES - A REVIEW OF 77 PATIENTS | 1993 | A. A. Nicholson; C. M. S. Royston |
| Pancreas-preserving duodenectomy after living donor liver transplantation for invasive cytomegalovirus disease | 2017 | R. Vincenzi; E. A. Fonseca; P. Chapchap; M. C. C. Machado; K. M. O. Roda; H. L. Candido; M. R. Benavides; M. A. D'Assuncao; R. C. Afonso; P. Turine; F. P. Marson; J. S. Neto |
| Pancreatic Cancer: The Great Masquerader | 2015 | A. Kahlon; P. Wander; D. Lowe; W. Syed; A. Dhillon; F. Allam |
| Pancreatic Head Tumor in an Infant With New-Onset Jaundice | 2015 | D. Heintz; S. Megison; S. Cope-Yokoyama; A. Goyal |
| Pancreatic Tumor a Rare Subtype | 2016 | B. A. Altamimi |
| Pancreaticoduodenectomy for periampullary malignancies: the effect of bile colonization on the postoperative outcome | 2007 | A. M. Isla; J. Griniatsos; A. Riaz; E. Karvounis; R. C. N. Williamson |
| Paraneoplastic cholestasis associated with prostate carcinoma | 2004 | M. Koruk; M. Büyükberber; C. Savaş; A. Kadayifçi |
| Partial internal biliary diversion for Alagille syndrome: Case report and review of the literature | 2012 | S. Sheflin-Findling; R. Arnon; S. Lee; J. Chu; F. Henderling; N. Kerkar; K. Iyer |
| Pathogenesis and management of pruritus in PBC and PSC | 2015 | A. E. Kremer; B. Namer; R. Bolier; M. J. Fischer; R. P. Oude Elferink; U. Beuers |
| Pathogenic Novel Heterozygous Variant c.1076c>T p. (Ser359Phe) chr1: 120512166 in NOTCH2 Gene, Type 2 Alagille Syndrome Causing Neonatal Cholestasis: A Case Report | 2022 | M. S. Uddin; S. Al Fulayyih; F. F. Al Denaini; M. M. Al Hatlani |
| Pathologic complete response after gemcitabine and S-1 chemotherapy for far advanced intrahepatic cholangiocarcinoma | 2018 | T. Tatsuguchi; K. Gotoh; S. Kobayashi; K. Asukai; A. Tomokuni; H. Akita; H. Wada; H. Takahashi; M. Ohue; M. Yano; M. Sakon |
| Pathology in practice | 2021 | S. J. Reeves; S. G. M. Kirejczyk; E. W. Howerth |
| Pathophysiological consequences of obstructive jaundice and perioperative management | 2018 | E. T. Pavlidis; T. E. Pavlidis |
| Pediatric living donor liver transplantation in the United States: A retrospective review of the national experience | 2019 | D. Yoeli; R. Choudhury; T. Nydam; A. Rana; M. Adams; J. Pomposelli; M. Wachs; E. Pomfret |
| Pediatric living donor liver transplantation: A retrospective review of the national experience | 2019 | D. Yoeli; T. Nydam; A. Rana; M. Adams; J. Pomposelli; M. Wachs; E. Pomfret |
| Peliosis hepatis as a late and fatal complication of thorotrast liver disease. Report of five cases | 1981 | K. Okuda; M. Omata; Y. Itoh |
| Percutaneous biliary drainage in malign obstructive jaundice: Is it really necessary for all patients with malign obstructive jaundice? | 2016 | C. Hocazade; I. Akmangit; B. Sever Sayin; M. Doǧan; Y. Bozkaya; G. U. Erdem; N. Zengin |
| Percutaneous biliary metal wall stenting in malignant obstructive jaundice | 2003 | A. A. Indar; D. N. Lobo; A. D. Gilliam; R. Gregson; I. Davidson; S. Whittaker; J. Doran; B. J. Rowlands; I. J. Beckingham |
| Percutaneous biliary stent with intraluminal brachytherapy versus palliative surgery in the management of extrahepatic cholangiocarcinoma | 2021 | Y. Wang; Z. Man; X. Hu; L. Zhou; H. Jin; H. Liu; Q. Pang |
| Percutaneous drainage of malignant biliary obstruction: Clinical outcomes | 2016 | W. Tuqan; A. Innabi; A. Alawneh; M. Khatib |
| Percutaneous microwave ablation versus surgical resection for ovarian cancer liver metastasis | 2020 | S. Zhuo; J. Zhou; G. Ruan; S. Zeng; H. Ma; C. Xie; C. An |
| Percutaneous transhepatic biliary drainage (PTBD) for gastrointestinal adenocarcinoma with liver metastasis | 2010 | M. Duxbury; S. Wigmore; R. Ravindran |
| Percutaneous transhepatic biliary drainage and stenting: A single centre experience | 2015 | V. Kronsten; A. Speirs; F. Ahmad; M. Gibson; J. Booth; N. Chandra |
| Percutaneous transhepatic cholangial drainage combined with percutaneous endoscopic jejunostomy for maintaining nutrition state in patients with advanced ampullary neoplasms | 2018 | Y. Sun; W. Li; D. Sun; S. Li; Q. Xu; Y. Li; Y. Lin; Y. Qi; T. Yang; K. Su; Y. Cen; X. Chen; P. Xu |
| Percutaneous transhepatic portal embolization using foam ethanolamine oleate and carbon dioxide (CO: A pilot study | 2015 | S. Inoue; O. Ikeda; Y. Nakasone; T. Beppu; T. Masuda; K. Yokoyama; D. Utsunomiya; H. Baba; Y. Yamashita |
| Percutaneous-endoscopic rendezvous technique for treatment of malignant biliary obstruction in a gastric cancer patient after billroth II gastrectomy | 2021 | L. Siregar; I. M. Loho; A. S. Waspodo; H. Saulata; V. A. Akbar; R. Swadari |
| Performance status is a good predictor for selective internal radiation therapy (S.I.R.T.) treatment of neoplastic liver lesions | 2020 | M. G. G. Bavetta; M. R. Barcellona; F. Bronte; S. Ialuna; L. Coniglio; D. Scalisi; F. D'Amato; G. Malizia; F. Verderame; F. Valenza; R. Virdone |
| PERFORMANCE STATUS IS A GOOD PREDICTOR FOR SELECTIVE INTERNAL RADIATION THERAPY (SIRT) TREATMENT OF NEOPLASTIC LIVER LESIONS | 2020 | M. G. G. Bavetta; M. R. Barcellona; F. Bronte; S. Ialuna; L. Coniglio; D. Scalisi; F. D'Amato; G. Malizia; F. Verderame; F. Valenza; R. Virdone; L. Multidisciplinary Oncology |
| Periampullary carcinoid: An uncommon tumor at an unusual site | 2011 | A. Somani; A. K. Jain; V. K. Dixit |
| Perihilar cholangiocarcinoma arising in hepatitis C virus-related liver cirrhosis with hepatocellular carcinoma | 2007 | T. Fujii; Y. Zen; Y. Nakanuma |
| Perioperative hepatic functional risk assessed with technetium-99m diethylenetriamine pentaacetic acid-galactosyl human serum albumin liver scintigraphy in patients undergoing pancreaticoduodenectomy complicated by obstructive jaundice | 1999 | H. Nakano; K. Kumada; Y. Takekuma; S. Hasebe; Y. Yoshizawa; M. Yamaguchi; D. Jaeck |
| Perioperative von Willebrand factor dynamics are associated with liver regeneration and predict outcome after liver resection | 2018 | P. Starlinger; D. Pereyra; S. Haegele; P. Braeuer; L. Oehlberger; F. Primavesi; A. Kohler; F. Offensperger; T. Reiberger; A. Ferlitsch; B. Messner; G. Beldi; S. Staettner; C. Brostjan; T. Gruenberger |
| Peripheral Primitive Neuroectodermal Tumor (pPNET): A Rare Cause of Elevated Liver Enzymes | 2012 | J. Eckert |
| Personalised radioembolization improves outcomes in refractory intra-hepatic cholangiocarcinoma: a multicenter study | 2019 | H. Levillain; I. D. Derijckere; L. Ameye; T. Guiot; A. Braat; C. Meyer; B. Vanderlinden; N. Reynaert; A. Hendlisz; M. Lam; C. M. Deroose; H. Ahmadzadehfar; P. Flamen |
| Pharmacokinetics and safety of infigratinib (BGJ398) in subjects with chronic hepatic impairment and in matched healthy adults | 2021 | M. Reyes; S. Guptha; E. Fuentes; C. Zamora; S. Andrews; K. Roupe; S. Moran; D. Martin |
| Pharmacologically stimulated portal flow measurement by magnetic resonance imaging for assessment of liver function | 1999 | S. Nakano; T. Katoh; M. Ohki; Y. Mori; J. Kageyama; Y. Toyama; I. Hino; K. Satoh; M. Ohkawa |
| Phase I and pharmacokinetic evaluation of the anti-telomerase agent KML-001 with cisplatin in advanced solid tumors | 2016 | M. J. Edelman; R. Lapidus; J. Feliciano; M. Styblo; J. H. Beumer; T. Liu; J. Gobbru |
| Photodynamic therapy with indwelling self-expandable metal stents is not associated with worse survival in patients with unresectable hilar cholangiocarcinoma | 2011 | J. Tian; T. H. Baron; E. C. Gorospe; G. A. Prasad; L. S. Lutzke; K. K. Wang |
| PHOTOSTENT-02: Porfimer sodium photodynamic therapy plus stenting versus stenting alone in patients with locally advanced or metastatic biliary tract cancer | 2018 | S. P. Pereira; M. Jitlal; M. Duggan; E. Lawrie; S. Beare; P. O'Donoghue; H. S. Wasan; J. W. Valle; J. Bridgewater |
| PIVKA-II is a useful marker in patients with modified UICC T3 stage hepatocellular carcinoma | 2013 | J. M. Kim; C. H. D. Kwon; J. W. Joh; J. B. Park; J. H. Lee; S. J. Kim; S. W. Paik; C. K. Park |
| Population Pharmacokinetics of Methadone after Oral Administration in Japanese Patients with Cancer-Related Pain | 2020 | H. Kokubun; C. Takigawa; S. Chihara; S. Hara; Y. Uezono |
| Portal vein thrombosis as a cause of massive ascites in a non-cirrhotic patient | 2015 | C. B. Ramirez; I. Preeshagul; J. G. Sanchez; J. A. Shrensel; M. Kutner; K. Favila |
| Post-cholecystectomy biliary strictures: Not always benign | 2008 | A. Sharma; A. Behari; S. S. Sikora; A. Kumar; R. Saxena; V. K. Kapoor |
| Post-ERCP pancreatitis and its related factors: A prospective study in Cipto Mangunkusumo National General Hospital | 2015 | D. Makmun; M. Abdullah; A. F. Syam; A. Fauzi |
| Post-operative ascites of unknown origin after laparoscopic cholecystectomy: Case report | 2022 | B. Alavi Farzaneh; M. Alipour; V. Reisi-Vanani |
| Postoperative pulmonary edema, transfusion-related? - A case report | 2003 | Y. T. Chung; Y. C. Wu; Y. H. Chen |
| Potential Efficacy of Allergen Removed Rhus Verniciflua Stokes Extract to Maintain Progression-Free Survival of Patients With Advanced Hepatobiliary Cancer | 2018 | J. Chae; S. Lee; S. Lee |
| Potential of Lenvatinib for an Expanded Indication from the REFLECT Trial in Patients with Advanced Hepatocellular Carcinoma | 2020 | S. Maruta; S. Ogasawara; Y. Ooka; M. Obu; M. Inoue; N. Itokawa; Y. Haga; A. Seki; S. Okabe; R. Azemoto; E. Itobayashi; M. Atsukawa; N. Sugiura; H. Mizumoto; K. Koroki; K. Kanayama; H. Kanzaki; K. Kobayashi; S. Kiyono; M. Nakamura; N. Kanogawa; T. Saito; T. Kondo; E. Suzuki; S. Nakamoto; A. Tawada; T. Chiba; M. Arai; T. Kanda; H. Maruyama; N. Kato |
| Pre operative radiation for icteric type hepatocellular carcinoma: A case report | 2008 | A. Nugroho; T. J. M. Lalisang; S. Gondhowiarjo |
| Predicting (avoiding) incidental gallbladder cancer | 2014 | K. Patel; A. Sautter; T. Buddensick; B. Wu; H. Ferdosi; D. Narducci; M. Siddique; L. Setiawan; H. Shaukat; G. Sulkowski; S. O. Farooqui; G. C. Kowdley; S. C. Cunningham |
| Predicting Disease-Specific Survival for Patients With Primary Cholangiocarcinoma Undergoing Curative Resection by Using a Decision Tree Model | 2022 | B. Quan; M. Li; S. Lu; J. Li; W. Liu; F. Zhang; R. Chen; Z. Ren; X. Yin |
| Predicting early mortality following percutaneous stent insertion for malignant biliary obstruction: a multivariate risk factor analysis | 2000 | R. Rai; R. Dick; N. Doctor; N. Dafnios; R. Morris; B. R. Davidson |
| Predicting Operative Outcomes in Patients with Liver Disease: Albumin-Bilirubin Score vs Model for End-Stage Liver Disease-Sodium Score | 2021 | G. A. Taylor; A. M. Fagenson; L. E. Kuo; H. A. Pitt; K. N. Lau |
| Prediction Efficacy of Prognostic Nutritional Index and Albumin-Bilirubin Grade in Patients With Intrahepatic Cholangiocarcinoma After Radical Resection: A Multi-Institutional Analysis of 535 Patients | 2021 | Q. Li; C. Chen; J. Zhang; H. Wu; Y. Qiu; T. Song; X. Mao; Y. He; Z. Cheng; W. Zhai; J. Li; D. Zhang; Z. Geng; Z. Tang |
| Prediction of 90Y selective internal radiation therapy outcome using pre therapeutic biomarkers in unresectable and refractory intra-hepatic cholangiocarcinoma patients | 2019 | H. Levillain; I. D. Derijckere; L. Ameye; T. Guiot; A. Braat; C. Meyer; B. Vanderlinden; N. Reynaert; A. Hendlisz; M. Lam; C. Deroose; H. Ahmadzadehfar; P. Flamen |
| Prediction of overall survival in resectable intrahepatic cholangiocarcinoma: ISICC-applied prediction model | 2020 | M. Tian; W. Liu; C. Tao; Z. Tang; Y. Zhou; S. Song; L. Jin; H. Wang; X. Jiang; P. Zhou; Y. Fang; W. Qu; Z. Ding; Y. Peng; X. Fu; S. Qiu; J. Zhou; J. Fan; Y. Shi |
| Prediction of portal pressure from intraoperative ultrasonography | 2014 | A. Nanashima; T. Abo; J. Arai; T. Tominaga; K. Takagi; K. Mochinaga; K. Furukawa; T. Nagayasu |
| Prediction of Post-hepatectomy Liver Failure in Patients With Hepatocellular Carcinoma Based on Radiomics Using Gd-EOB-DTPA-Enhanced MRI: The Liver Failure Model | 2021 | Y. Chen; Z. Liu; Y. Mo; B. Li; Q. Zhou; S. Peng; S. Li; M. Kuang |
| Prediction of Posthepatectomy Liver Failure Proposed by the International Study Group of Liver Surgery | 2018 | Y. Mizutani; T. Hirai; S. Nagamachi; A. Nanashima; K. Yano; K. Kondo; M. Hiyoshi; N. Imamura; T. Terada |
| Prediction of the Remnant Liver Hypertrophy Ratio after Preoperative Portal Vein Embolization | 2013 | Y. Kasai; E. Hatano; K. Iguchi; S. Seo; K. Taura; K. Yasuchika; A. Mori; T. Kaido; S. Tanaka; T. Shibata; T. Shibata; S. Uemoto |
| Predictive factors associated with cholangitis following endoscopic retrograde cholangiopancreatography | 2017 | J. Tierney; N. Bhutiani; B. Stamp; J. Richey; M. H. Bahr; G. C. Vitale |
| Predictive Factors for Post-Hepatectomy Liver Failure in Patients with Cholangiocarcinoma | 2023 | W. Kriengkrai; B. Somjaivong; A. Titapun; P. Wonggom |
| Predictive factors for survival time of inoperable hilar cholangiocarcinoma patients | 2016 | S. Srikhajonjit; P. Mairiang; E. Mairiang; A. Sangchan; K. Sawadpanitch; Y. Thavornpitak |
| PREDICTIVE FACTORS FOR THE EFFECTIVENESS OF BILIARY DECOMPRESSION USING ENDOBILIARY STENT IN PATIENTS WITH UNRESECTABLE MALIGNANT BILIARY OBSTRUCTION | 2020 | P. Termsinsuk; N. Pausawasdi; P. Charatcharoenwitthaya; J. Limsrivilai; U. Kaosombatwattana; M. Rugivarodom; K. Maipang |
| Predictive Factors of Chemotherapy Initiation after Biliary Drainage for Advanced Biliary Tract Cancer: A Retrospective Multicenter Study | 2021 | M. Azarfane; A. Lievre; H. Senellart; B. Dessomme; P. Guillouche; J. Meyer; J. Bennouna; T. Wallenhorst; M. Salimon; J. Gournay; T. Matysiak-Budnik; A. Lim; J. Edeline; Y. Touchefeu |
| Predictive risk factors associated with cholangitis following ERCP | 2018 | J. Tierney; N. Bhutiani; B. Stamp; J. S. Richey; M. H. Bahr; G. C. Vitale |
| Predictive value of indocyanine green plasma disappearance rate on liver function and complications after liver transplantation | 2018 | Y. Sun; L. Yu; Y. Liu |
| Predictive value of risk factors in patients with obstructive jaundice | 2004 | M. Pitiakoudis; K. Mimidis; A. K. Tsaroucha; V. Papadopoulos; A. Karayiannakis; C. Simopoulos |
| Predictive value of the albumin-bilirubin grade on long-term outcomes of CT-guided percutaneous microwave ablation in intrahepatic cholangiocarcinoma | 2019 | J.-Y. Ni; C. An; T.-Q. Zhang; Z.-M. Huang; X.-Y. Jiang; J.-H. Huang |
| Predictors of septic shock in initially stable patients with pyogenic liver abscess | 2017 | H. Cho; E. S. Lee; Y. S. Lee; Y. J. Kim; C. H. Sohn; S. Ahn; D. W. Seo; J. H. Lee; W. Y. Kim; K. S. Lim |
| Predictors of stent dysfunction in palliative endoscopic drainage of malignant biliary obstruction | 2013 | S. Khorrami; E. Iyo; C. Paez; C. Garrido; A. Llompart |
| Predictors of unresectable proximal cholangiocarcinoma in potentially resectable patients | 2018 | P. Muangkaew; S. Mingphruedhi; N. Rungsakulkij; P. Tangtawee; P. Sompoppokaset; W. Suragul |
| Prednisolone: Role in amoxicillin-clavulanate-induced cholestatic liver injury | 2021 | M. Q. Lee; R. Chigozie; I. Khan; G. O'Mara |
| Pre-hepatectomy gamma-GTP concentration predicts postoperative liver function recovery | 2017 | M. Ishii; Y. Hirano; H. Kashiwagi; M. Nishioka; H. Iio; Y. Kabeshima |
| Preliminary results from a phase Ib study of neoadjuvant ipilimumab plus nivolumab prior to liver resection for hepatocellular carcinoma: The PRIME-HCC trial | 2022 | A. D'Alessio; M. Pai; D. Spalding; P. Rajagopal; T. Talbot; R. Goldin; C. A. M. Fulgenzi; C. Ward; V. Yip; S. Slater; M. Sodergren; P. Tait; N. A. Habib; R. Thomas; A. Cortellini; R. Sharma; D. J. J. Pinato |
| Premalignant lesions of cholangiocarcinoma: characteristics on ultrasonography and MRI | 2019 | S. Siripongsakun; W. Sapthanakorn; P. Mekraksakit; S. Vichitpunt; S. Chonyuen; J. Seetasarn; S. Bhumiwat; T. Sricharunrat; S. Srittanapong |
| Preoperative 3D reconstruction and fluorescent indocyanine green for laparoscopic duodenum preserving pancreatic head resection: A case report | 2023 | X. L. Li; L. S. Gong |
| Preoperative biliary drainage in pancreatic head cancer patients | 2018 | E. S. Aljahdli |
| Preoperative biliary stenting and major morbidity after pancreatoduodenectomy: Does elapsed time matter? The Fragerita Study Group | 2018 | M. Sandini; K. C. Honselmann; D. J. Birnbaum; F. Gavazzi; M. Chirica; U. Wellner; T. Guilbaud; L. Bolm; M. Angrisani; V. Moutardier; M. Cereda; É. Girard; M. Montorsi; T. Keck; A. Zerbi; L. Gianotti |
| Preoperative CEA levels are supplementary to CA19-9 levels in predicting prognosis in patients with resectable intrahepatic cholangiocarcinoma | 2018 | C. He; Y. Zhang; Y. Song; J. Wang; K. Xing; X. Lin; S. Li |
| Preoperative Cholangitis and Future Liver Remnant Volume Determine the Risk of Liver Failure in Patients Undergoing Resection for Hilar Cholangiocarcinoma | 2016 | D. Ribero; G. Zimmitti; T. A. Aloia; J. Shindoh; F. Forchino; M. Amisano; G. Passot; A. Ferrero; J.-N. Vauthey |
| Preoperative enteral nutritional support in patients undergoing hepatectomy for hepatocellular carcinoma a strengthening the reporting of observational studies in epidemiology article | 2015 | H. Yao; X. Bian; L. Mao; X. Zi; X. Yan; Y. Qiu |
| Preoperative indocyanine green (ICG) clearance test: Can we really trust it to predict post hepatectomy liver failure? A systematic review of the literature and meta-analysis of diagnostic test accuracy | 2022 | S. Granieri; G. Bracchetti; A. Kersik; S. Frassini; A. Germini; A. Bonomi; L. Lomaglio; E. Gjoni; A. Frontali; F. Bruno; S. Paleino; C. Cotsoglou |
| Preoperative management protocol for perihilar cholangiocarcinoma – A Survey of 2778 cases in Japan | 2018 | R. J. Chaudhary; R. Higuchi; M. Nagino; K. Hasegawa; I. Endo; T. Wakai; M. Ohtsuka; M. Unno; S. Hirano; M. Yamamoto |
| Preoperative neutrophil-to-lymphocyte ratio as a prognostic marker in patients with gallbladder carcinoma | 2018 | J. Yao; S. Gao; J. Luo |
| Preoperative peripheral blood inflammatory markers especially the fibrinogen-to-lymphocyte ratio and novel FLR-N score predict the prognosis of patients with early-stage resectable extrahepatic cholangiocarcinoma | 2022 | S. Li; X. Zhang; C. Lou; Y. Gu; J. Zhao |
| Preoperative Predictors for 90-Day Mortality after Pancreaticoduodenectomy in Patients with Adenocarcinoma of the Ampulla of Vater: A Single-Centre Retrospective Cohort Study | 2021 | R. Fernandez-Placencia; F. Berrospi-Espinoza; K. Uribe-Rivera; J. Medina-Cana; I. Chavez-Passiuri; N. Sanchez-Bartra; K. Paredes-Galvez; C. Luque-Vasquez Vasquez; J. Celis-Zapata; E. Ruiz-Figueroa |
| Preoperative predictors of conversion as indicators of local inflammation in acute cholecystitis: strategies for future studies to develop quantitative predictors | 2018 | R. Z. Panni; S. M. Strasberg |
| Preoperative Resolution of Jaundice Following Biliary Stenting Predicts More Favourable Early Survival in Resected Pancreatic Ductal Adenocarcinoma | 2008 | R. A. Smith; K. Dajani; S. Dodd; P. Whelan; M. Raraty; R. Sutton; F. Campbell; J. P. Neoptolemos; P. Ghaneh |
| Preoperative risk factors for the early failure of the Kasai portoenterostomy in patients with biliary atresia | 2021 | M. A. Capparelli; V. H. Ayarzabal; E. T. Halac; H. A. Questa; M. J. Minetto; G. Cervio; M. E. Barrenechea |
| Preoperative Risk Score and Prediction of Long-Term Outcomes after Hepatectomy for Intrahepatic Cholangiocarcinoma | 2018 | K. Sasaki; G. A. Margonis; N. Andreatos; F. Bagante; M. Weiss; C. Barbon; I. Popescu; H. P. Marques; L. Aldrighetti; S. K. Maithel; C. Pulitano; T. W. Bauer; F. Shen; G. A. Poultsides; O. Soubrane; G. Martel; B. G. Koerkamp; A. Guglielmi; E. Itaru; F. N. Aucejo; T. M. Pawlik |
| Preoperative Serum Markers and Risk Classification in Intrahepatic Cholangiocarcinoma: A Multicenter Retrospective Study | 2022 | M. Kaibori; K. Yoshii; H. Kosaka; M. Ota; K. Komeda; M. Ueno; D. Hokutou; H. Iida; K. Matsui; M. Sekimoto |
| Preoperative serum total bilirubin level predicts therapeutic outcome after pancreaticoduodenectomy for pancreatic ductal adenocarcinoma | 2015 | N. Okui; H. Shiba; Y. Shirai; T. Sakamoto; K. Furukawa; R. Iwase; T. Horiuchi; Y. Fujiwara; K. Haruki; K. Yanaga |
| Prevalence and characteristics of non-cirrhotic patients with thrombosis of the portal system | 2020 | C. A. Campoverde-Espinoza; F. Higuera de la Tijera; J. A. Meléndez-Andrade; A. Servín-Caamaño |
| Prevalence and clinical relevance of serum anti-p53 antibodies in patients with cholangiocarcinoma | 2000 | P. Tangkijvanich; K. Kasemsupatana; A. Janchai; P. Kullavanijaya; A. Theamboonlers; Y. Poovorawan |
| Prevalence of gallbladder sludge and associated abnormalities in cats undergoing abdominal ultrasound | 2022 | J. Villm; S. DeMonaco; M. Larson |
| Prevalence of hepatobiliary dysfunction in a regional group of patients with chronic inflammatory bowel disease | 1991 | V. Wewer; C. Gluud; P. Schlichting; F. Burcharth; V. Binder |
| Preventive Effect of High-Dose Digestive Enzyme Management on Development of Nonalcoholic Fatty Liver Disease after Pancreaticoduodenectomy: A Randomized Controlled Clinical Trial | 2020 | K. Yasukawa; A. Shimizu; T. Yokoyama; K. Kubota; T. Notake; H. Seki; A. Kobayashi; Y. Soejima |
| Primary amyloidosis presenting as non-obstructive biliary dilation | 2021 | A. Sharma; A. Syed; T. Singh |
| Primary biliary cirrhosis and granulomatous pulmonary infiltrates: Causal association or casual association? | 2011 | T. Suehiro; M. Matsushita; T. Matsui; H. Kanayama; M. Morishita; Y. Nakamura; Y. Kobayashi; M. Nakano |
| Primary cancer of the liver: a clinical study | 1973 | K. J. Rostock; R. Claus |
| Primary carcinoma of the liver | 1974 | I. Honjo; R. Mizumoto |
| Primary carcinoma of the liver. Clinical aspects--laparoscopy--functional diagnosis and differential diagnosis of hepatic cirrhosis | 1970 | H. Ortmans; H. Selmair; E. Wildhirt |
| Primary Hepatic Carcinosarcoma Composed of Hepatocellular Carcinoma, Cholangiocarcinoma, Osteosarcoma and Rhabdomyosarcoma With Poor Prognosis | 2020 | L. Liu; E. Ahn; K. Studeman; K. Campbell; J. Lai |
| Primary hepatic lymphoma mimicking type IV cholangio-carcinoma with complete resolution following chemotherapy | 2017 | P. Pal; S. Lakhtakia; A. Sekharan; P. N. Rao; D. N. Reddy |
| Primary Hodgkin lymphoma masquerading as a Klatskin tumor | 2011 | P. Kulkarni; N. Karamsadkar; D. N. Socoloff |
| Primary langerhans cell histiocytosis of the extrahepatic bile duct occurring in an adult patient | 2018 | I. E. Obiorah; A. H. Velasquez; B. Kallakury; M. Özdemirli |
| Primary liposarcoma of the duodenum: A first case presentation | 2013 | T. Okabayashi; Y. Shima; J. Iwata; T. Sumiyoshi; A. Kozuki; T. Tokumaru; Y. Hata; Y. Noda; T. Inagaki; S. Morishita; M. Morita |
| Primary Malignant Rhabdoid Tumour of the Liver in Adult Male: a Diagnostic and Therapeutic Challenge | 2021 | S. Pasricha; G. Durga; G. Gupta; A. Jajodia; V. P. B. Koyyala; A. Sharma; M. Kamboj; M. Gupta; A. Mehta |
| Primary mucosa-associated lymphoid tissue lymphoma of the hilar bile duct resulting in fluctuant jaundice A case report | 2018 | Z. Liu; Y. Zang; X. Wang; N. Li; D. Lin |
| Primary neuroendocrine tumors of the gallbladder: Ultrasonographic and MDCT features with pathologic correlation | 2015 | H. El Fattach; Y. Guerrache; C. Eveno; M. Pocard; R. Kaci; C. Shaar-Chneker; R. Dautry; M. Boudiaf; A. Dohan; P. Soyer |
| Primary sclerosing cholangitis in children versus adults: lessons for the clinic | 2018 | A. Adike; E. J. Carey; K. D. Lindor |
| Primary sclerosing cholangitis is changing clinical spectrum and old biomarkers disclose an innovative role: The case of alkaline phosphatase | 2011 | P. Invernizzi |
| Primary Sclerosing Cholangitis Risk Estimate Tool (PREsTo) Predicts Outcomes of the Disease: A Derivation and Validation Study Using Machine Learning | 2020 | J. E. Eaton; M. Vesterhus; B. M. McCauley; E. J. Atkinson; E. M. Schlicht; B. D. Juran; A. A. Gossard; N. F. LaRusso; G. J. Gores; T. H. Karlsen; K. N. Lazaridis |
| Primary sclerosing cholangitis: Epidemiology, natural history, and prognosis | 2006 | C. Levy; K. D. Lindor |
| Primary squamous cell carcinoma of extrahepatic bile duct associated with choledochal cyst: A case report and literature review | 2018 | A. Tongyoo; C. Thamwongskul; E. Sriussadaporn; P. Limpavitayaporn; C. Mingmalairak |
| Primary undifferentiated carcinoma with osteoclast-like giant cells in liver and rapidly developing multiple metastases after curative hepatectomy: a case report | 2020 | N. Kamitani; T. Nomi; D. Hokuto; T. Yoshikawa; Y. Matsuo; M. Sho |
| PRIME-HCC: phase Ib study of neoadjuvant ipilimumab and nivolumab prior to liver resection for hepatocellular carcinoma | 2022 | A. D'Alessio; M. Pai; D. Spalding; P. Rajagopal; T. Talbot; R. Goldin; C. A. Maria Fulgenzi; C. Ward; V. Yip; T. Dhillon; S. Slater; M. Sodergren; P. Tait; N. Habib; R. Thomas; A. Cortellini; R. Sharma; D. J. Pinato |
| Procalcitonin is a useful biomarker to predict severe acute cholangitis: a single-center prospective study | 2017 | G. Umefune; H. Kogure; T. Hamada; H. Isayama; K. Ishigaki; K. Takagi; D. Akiyama; T. Watanabe; N. Takahara; S. Mizuno; S. Matsubara; N. Yamamoto; Y. Nakai; M. Tada; K. Koike |
| Procedure Time, Efficacy, and Safety of Portal Vein Embolisation Using a Sheathless Needle-Only Technique Compared with Traditional Technique | 2022 | K. C. H. Yu; S. S. M. Wong; Y. C. Wong; C. B. Tan; J. C. W. Siu; H. Y. Lau; J. C. X. Chan; C. S. C. Tsai; S. C. H. Yu |
| Profile and Outcome of Patients with Acute Cholangitis in a Tertiary Center in South India | 2021 | P. Raghhupatruni; R. Gopalakrishna; V. Ankarath; S. Sadasivan |
| Prognosis and incidence of immunological and oncological complications after direct-acting antiviral therapy for chronic hepatitis C | 2022 | Y. Kanayama; K. Sato; S. Saito; T. Ueno; Y. Shimada; T. Kohga; M. Shibasaki; A. Naganuma; S. Takakusagi; T. Nagashima; H. Nakajima; H. Takagi; D. Uehara; T. Uraoka |
| Prognosis of 84 intrahepatic cholangiocarcinoma patients | 2009 | F. H. Li; X. Q. Chen; H. Y. Luo; Y. H. Li; F. Wang; M. Z. Qiu; K. Y. Teng; Z. H. Li; R. H. Xu |
| Prognosis of Hepatocellular Carcinoma Patients Who Achieved Long-Term Recurrence-Free Survival After Curative Therapy: Impact of the ALBI Grade | 2018 | H. Matsushima; Y. Takami; T. Ryu; M. Yoshitomi; M. Tateishi; Y. Wada; H. Saitsu |
| Prognosis of intrahepatic cholangiocarcinoma stratified by albumin-bilirubin grade | 2021 | S. Kaneko; M. Kurosaki; K. Tsuchiya; Y. Yasui; K. Inada; S. Kirino; K. Yamashita; L. Osawa; Y. Hayakawa; S. Sekiguchi; M. Higuchi; K. Takaura; C. Maeyashiki; N. Tamaki; J. Itakura; Y. Takahashi; H. Nakanishi; D. Asano; T. Irie; Y. Kawachi; N. Izumi |
| Prognosis of late elderly patients with chronic hepatitis C after achieving a sustained viral response by direct-acting antivirals | 2021 | S. Takakusagi; H. Takagi; T. Kosone; K. Sato; S. Kakizaki; T. Uraoka |
| Prognosis of Malignant Obstructive Jaundice Following Surgery for Gastric Carcinoma | 2003 | J. H. Song; K. Y. Yoon; S. H. Lee |
| Prognosis of primary sclerosing cholangitis – time to look at the population as a whole, not only from the center's or waiting list perspective | 2018 | W. O. Bechstein; A. A. Schnitzbauer |
| Prognosis of the intrahepatic cholangiocarcinoma after resection: Hepatitis B virus infection and adjuvant chemotherapy are favorable prognosis factors | 2013 | R. Q. Liu; S. J. Shen; X. F. Hu; J. Liu; L. J. Chen; X. Y. Li |
| Prognostic analysis of patients suffering from distal bile duct cancer | 2004 | L. C. Sun; B. H. Zhang; Y. J. Zhang; X. Q. Jiang; B. H. Zhang; B. Yi; W. L. Yu; M. C. Wu |
| Prognostic analysis of patients suffering from intrahepatic cholangiocarcinoma | 2011 | Z. Chen; J. Yan; L. Huang; Y. Yan |
| Prognostic factors for benefits from percutaneous transhepatic biliary drainage | 1991 | M. C. Castoldi; P. Pisani; G. Ideo; M. Bellomi; G. Cozzi; M. A. Pestalozza; A. Severini |
| Prognostic factors for survival after pancreaticoduodenectomy for patients with carcinoma of the pancreatic head region | 1995 | J. H. Allema; M. E. Reinders; T. M. Van Gulik; M. J. W. Koelemay; D. J. Van Leeuwen; L. T. De Wit; D. J. Gouma; H. Obertop |
| Prognostic factors in liver tumors | 1995 | C. Wittekind |
| Prognostic factors of mortality in the malignant biliary obstruction unresectable after the insertion of an endoscopic stent | 2006 | A. Hernández Guerrero; J. Sánchez del Monte; S. Sobrino Cossío; O. Alonso Lárraga; L. Delgado de la Cruz; M. M. Frías Mendívil; C. M. Frías Mendívil |
| Prognostic impact of hepatitis B virus (HBV) infection in advanced intrahepatic cholangiocarcinoma (iCCA) patients (pts) treated with first-line gemcitabine plus cisplatin (GEMCIS) | 2017 | E. J. Kim; H. Cho; C. Yoo; K. P. Kim; H. M. Chang; B. Y. Ryoo |
| Prognostic impact of transcatheter arterial chemoembolization (TACE) combined with radiofrequency ablation in patients with unresectable hepatocellular carcinoma: Comparison with TACE alone using decision-tree analysis after propensity score matching | 2019 | S. Shimose; M. Tanaka; H. Iwamoto; T. Niizeki; T. Shirono; H. Aino; Y. Noda; N. Kamachi; S. Okamura; M. Nakano; R. Kuromatsu; T. Kawaguchi; A. Kawaguchi; H. Koga; Y. Yokokura; T. Torimura |
| Prognostic implications of hepatitis B virus infection in intrahepatic cholangiocarcinoma treated with first-line gemcitabine plus cisplatin | 2018 | H. Chae; H. Choi; C. Yoo; K.-p. Kim; J. H. Jeong; H.-M. Chang; J. Kang; H. C. Lee; Y.-S. Lim; K. M. Kim; J. H. Shim; S. S. Lee; D. H. Park; T. J. Song; S. Hwang; G.-W. Song; D.-B. Moon; Y.-J. Lee; J. H. Lee; B.-Y. Ryoo |
| Prognostic importance of bile duct invasion in surgical resection with curative intent for hepatocellular carcinoma using PSM analysis | 2018 | X. Yang; Z. Qiu; R. Ran; L. Cui; X. Luo; M. Wu; W. F. Tan; X. Jiang |
| Prognostic Index for Localized Liver Radiation – Metastatic (PILLiR-M): Development and Analysis of a Clinical Prognostic Tool to Improve Patient Selection for Liver Directed Radiotherapy for Liver Metastases | 2019 | L. Callan; J. Vickress; E. Wong; M. I. Lock |
| Prognostic markers affecting the early recurrence of hepatocellular carcinoma with liver cirrhosis after curative resection | 2019 | W. J. Kim; T. W. Lim; P. J. Park; S. B. Choi; W. B. Kim |
| Prognostic Nomogram Based on Histological Characteristics of Fibrotic Tumor Stroma in Patients Who Underwent Curative Resection for Intrahepatic Cholangiocarcinoma | 2018 | C. Y. Jing; Y. P. Fu; J. L. Huang; M. X. Zhang; Y. Yi; W. Gan; X. Xu; H. J. Shen; J. J. Lin; S. S. Zheng; J. Zhang; J. Zhou; J. Fan; Z. G. Ren; S. J. Qiu; B. H. Zhang |
| Prognostic significance of neutrophil-lymphocyte ratio and carbohydrate antigen 19-9 in patients with gallbladder carcinoma | 2019 | F. Liu; H. J. Hu; W. J. Ma; Q. Yang; J. K. Wang; F. Y. Li |
| Prognostic significance of preoperative albumin-to-globulin ratio in patients with cholangiocarcinoma | 2017 | Q. Lin; Z. H. Lin; J. Chen; J. X. Lin; X. Li; J. R. Jiang; X. K. Ma; D. H. Wu; Z. H. Chen; M. Dong; L. Wei; T. T. Wang; D. Y. Ruan; Z. X. Lin; J. Y. Wen; X. Y. Wu; M. S. Huang |
| Prognostic Utility of Albumin-Bilirubin Grade for Short- and Long-Term Outcomes after Hepatic Resection for Intrahepatic Cholangiocarcinoma: A Multi-Institutional Analysis of 706 Patients | 2019 | D. I. Tsilimigras; J. M. Hyer; D. Moris; F. Bagante; A. Z. Paredes; T. M. Pawlik |
| Prognostic value of 18F-fluorodeoxyglucose positron emission tomography/computed tomography in patients with combined hepatocellular-cholangiocarcinoma | 2019 | C. H. Lim; S. H. Moon; Y. S. Cho; J. Y. Choi; K. H. Lee; S. H. Hyun |
| Prognostic value of inflammation-based markers in patients with recurrent malignant obstructive jaundice treated by reimplantation of biliary metal stents A retrospective observational study | 2017 | H. Jin; Q. Pang; H. Liu; Z. Li; Y. Wang; Y. Lu; L. Zhou; H. Pan; W. Huang |
| Prognostic value of inflammatory and tumour markers in small-duct subtype intrahepatic cholangiocarcinoma after curative-intent resection | 2021 | B. Ma; H. Meng; A. Shen; Y. Ma; D. Zhao; G. Liu; S. Zheng; Y. Tian; W. Zhang; Q. Li; S. Li |
| Prognostic value of paravertebral muscle density in patients with spinal metastases from gastrointestinal cancer | 2019 | S. Dohzono; R. Sasaoka; K. Takamatsu; M. Hoshino; H. Nakamura |
| Prognostication Systems as Applied to Primary and Metastatic Hepatic Malignancies | 2015 | C. S. Cho |
| Progression Patterns and Post-Progression Survival in Recurred Intrahepatic Cholangiocarcinoma Patients: A Novel Prognostic Nomogram Based on Multicenter Cohorts | 2022 | C. Zhao; C. He; J. Lu; X. Huang; C. Chen; X. Lin |
| Progressive atrophy in a deformed liver as a contributor to sigmoid volvulus | 2019 | T. Zenda; I. Araki; N. Hamano; E. Sawada; O. Nakamiya; T. Endo; H. Nishida; E. Ojima; T. Nakano |
| Proline supplementation mitigates the early stage of liver injury in bile duct ligated rats | 2019 | R. Heidari; H. Mohammadi; V. Ghanbarinejad; A. Ahmadi; M. M. Ommati; H. Niknahad; A. Jamshidzadeh; N. Azarpira; N. Abdoli |
| Prospective evaluation of gadoxetic acid magnetic resonance for the diagnosis of hepatocellular carcinoma in newly detected nodules ≤2 cm in cirrhosis | 2019 | C. Ayuso; A. Forner; A. Darnell; J. Rimola; Á. García-Criado; L. Bianchi; R. Vilana; R. Oliveira; N. Llarch; J. Bruix |
| Protective effects of Persea americana fruit and seed extracts against chemically induced liver cancer in rats by enhancing their antioxidant, anti-inflammatory, and apoptotic activities | 2022 | O. M. Ahmed; H. I. Fahim; E. E. Mohamed; A. Abdel-Moneim |
| Proton beam radiotherapy combined with anti-PD1/PDL1 immune checkpoint inhibitors for advanced hepatocellular carcinoma | 2022 | C. W. Su; M. M. Hou; P. W. Huang; Y. C. Chou; B. S. Huang; J. H. Tseng; C. W. Hsu; T. C. Chang; S. M. Lin; C. C. Lin |
| PTPRO-associated hepatic stellate cell activation plays a critical role in liver fibrosis | 2015 | X. Zhang; Z. Tan; Y. Wang; J. Tang; R. Jiang; J. Hou; H. Zhuo; X. Wang; J. Ji; X. Qin; B. Sun |
| Pure laparoscopic versus open liver resection for primary liver carcinoma in elderly patients a single-center, case-matched study | 2015 | X. T. Wang; H. G. Wang; W. D. Duan; C. Y. Wu; M. Y. Chen; H. Li; X. Huang; F. B. Zhang; J. H. Dong |
| Pure laparoscopic versus open major hepatectomy for hepatocellular carcinoma with liver F4 cirrhosis without routine Pringle maneuver – A propensity analysis in a single center | 2020 | T. T. Cheung; K. W. Ma; W. H. She; W. C. Dai; S. H. Y. Tsang; A. C. Y. Chan; C. M. Lo |
| Pyogenic liver abscess in a splenectomized diabetic: A case study | 2015 | A. Kotwal; C. Shanks; K. Abraham; M. M. Garcia |
| Pyogenic liver abscess: Review of 38 cases in cancer patients | 2017 | S. S. Jaiswal; S. Nanjappa; J. N. Greene |
| Pyogenic liver abscesses: a contemporary analysis of management strategies at a tertiary institution | 2017 | K. Rismiller; J. Haaga; C. Siegel; J. B. Ammori |
| Quality of life and survival analysis of patients undergoing transarterial chemoembolization for primary hepatic malignancies: A prospective cohort study | 2012 | K. M. Eltawil; R. Berry; M. Abdolell; M. Molinari |
| Quantifying the effects of absorbed dose from radioembolisation on healthy liver function with [99mTc]TcMebrofenin | 2020 | K. P. Willowson; G. P. Schembri; E. J. Bernard; D. L. Chan; D. L. Bailey |
| Radioembolization in patients with intrahepatic cholangiocarcinoma-a prognostic risk stratification model | 2017 | I. Schatka; H. Jochens; J. Rogasch; K. Huang; F. Wedel; U. Heimann; C. Bartel; C. Furth; W. Brenner; B. Gebauer; H. Amthauer |
| Radioembolization using 90Y-resin microspheres for patients with advanced hepatocellular carcinoma | 2006 | B. Sangro; J. I. Bilbao; J. Boan; A. Martinez-Cuesta; A. Benito; J. Rodriguez; A. Panizo; B. Gil; M. Inarrairaegui; I. Herrero; J. Quiroga; J. Prieto |
| Radioembolization with Yttrium-90 microspheres in unresectable primary and metastatic liver tumors | 2012 | L. Uslu; F. Gulsen; S. Sager; S. Pekmezci; M. Cantasdemir; L. Kabasakal; F. Numan; B. Kanmaz |
| Radioembolization with yttrium-90 resin microspheres for liver metastases of pancreatic adenocarcinoma: A retrospective multicenter analysis | 2017 | A. Y. Kim; S. Frantz; J. Zhang; N. M. Akhtar |
| Radiofrequency ablation of the main lesion of hepatocellular carcinoma and bile duct tumor thrombus as a radical therapeutic alternative | 2015 | J. Gao; Q. Zhang; J. Zhang; J. Kong; S. Wang; X. Ding; S. Ke; W. Sun |
| Radiological case of the month | 2007 | A. S. Kidwai; A. C. Friedman; C. B. Monteiro |
| Radiomics Analysis of Contrast-Enhanced CT for the Preoperative Prediction of Microvascular Invasion in Mass-Forming Intrahepatic Cholangiocarcinoma | 2021 | F. Xiang; S. Wei; X. Liu; X. Liang; L. Yang; S. Yan |
| Randomized clinical trial of the effect of intravenous fluid administration in patients with obstructive jaundice undergoing endoscopic drainage | 2005 | F. J. Padillo; J. Briceno; A. Cruz; M. Chicano; A. Naranjo; J. Vallejo; A. Martin-Malo; C. Pera-Madrazo; A. Sitges-Serra |
| Rapid Recovery of Postoperative Liver Function after Major Hepatectomy using Saline-linked Electric Cautery | 2008 | T. Mizuguchi; T. Katsuramaki; M. Nagayama; M. Meguro; T. Shibata; S. Kaji; K. Hirata |
| Rapid recurrence of spindle cell type undifferentiated carcinoma early after radical surgery in a bile duct cancer patient – A case report | 2021 | H. Kajioka; A. Muraoka |
| Rare association of microfilaria with poorly differentiated mucin-secreting metastatic carcinoma in liver aspirate cytology | 2019 | R. Gahine; A. Das; V. K. Jain; A. Agrawal |
| Rare event of biliary papillomatosis arising in a choledochal cyst | 2014 | K. Nishant; V. K. Singh; B. K. Sharma |
| Rare postoperative hemorrhage after robotic-assisted pancreatoduodenectomy for pancreatic head cancer: A case report | 2020 | J. J. Zhou; W. H. Chen; H. Zou; L. Xiong; X. Y. Miao; C. He; B. Shu; Y. Q. Zhou; D. L. Liu; Y. Wen |
| Rare sarcomatoid liver carcinoma composed of atypical spindle cells without features of either HCC or ICC: A case report | 2016 | K. Nirei; S. Matsuoka; M. Moriyama; H. Nakamura; T. Maebayashi; T. Takayama; M. Sugitani |
| Real-world outcomes of patients with advanced intrahepatic cholangiocarcinoma treated with programmed cell death protein-1-targeted immunotherapy | 2022 | M. Deng; S. Li; Q. Wang; R. Zhao; J. Zou; W. Lin; J. Mei; W. Wei; R. Guo |
| Reappraisal of the Role of Alkaline Phosphatase in Hepatocellular Carcinoma | 2022 | C. W. Huang; T. H. Wu; H. Y. Hsu; K. T. Pan; C. W. Lee; S. W. Chong; S. F. Huang; S. E. Lin; M. C. Yu; S. M. Chen |
| Recovery patterns of liver function after complete and partial surgical biliary decompression | 1996 | P. Watanapa |
| Recurrent acute portal vein thrombosis with severe abdominal infection after right hemihepatectomy in a patient with perihilar cholangiocarcinoma: A case report and literature review | 2022 | Q. Deng; M. He; Y. Yang; Y. Ou; Y. Cao; L. Zhang |
| Recurrent pyogenic cholangitis – an independent poor prognostic indicator for resectable intrahepatic cholangiocarcinoma: A propensity score matched analysis | 2018 | K. W. Ma; T. T. Cheung; W. H. She; K. S. H. Chok; A. C. Yan Chan; J. W. Chiu Dai; C. M. Lo |
| Reducing mortality and morbidity after pancreaticduodenal resection of periampullary tumor: A team experience at cipto mangunkusumo hospital Jakarta, Indonesia | 2016 | T. Lalisang; Y. Mazni; W. Je; M. Adhi Keswara |
| Reevaluation of prognostic markers in ascending cholangitis: Does charcot's triad matter? | 2016 | S. Mohapatra; F. John; P. Charilaou; A. Broder; C. S. Pitchumoni |
| Regional Infusion-Radioembolization | 2008 | G. J. Dubel; G. M. Soares |
| RELATIONSHIP BETWEEN CONTENT OF HEPATIC GLUTATHIONE S-TRANSFERASES AND THE KINETICS OF INDOCYANINE GREEN ELIMINATION IN VARIOUS LIVER-DISEASES | 1993 | M. Sugimoto; N. Shimada; K. Aikawa; Y. Sugiyama |
| Relevance of preoperative hyperbilirubinemia in patients undergoing hepatobiliary resection for hilar cholangiocarcinoma | 2019 | K. M. Wronka; M. Grąt; J. Stypułkowski; E. Bik; W. Patkowski; M. Krawczyk; K. Zieniewicz |
| Renal cell carcinoma presented with a rare case of icteric Stauffer syndrome: A case report | 2022 | D. R. Popov; K. A. Antonov; E. G. Atanasova; C. P. Pentchev; L. M. Milatchkov; M. D. Petkova; K. G. Neykov; R. K. Nikolov |
| Renal function in obstructive jaundice in man: Cholangiocarcinoma model | 1990 | V. Sitprija; U. Kashemsant; A. Sriratanaban; S. Arthachinta; V. Poshyachinda |
| Repeated hepatectomy for recurrent intrahepatic cholangiolocellular carcinoma: Report of a case | 2016 | M. Tomioku; N. Yazawa; D. Furukawa; H. Izumi; T. Mashiko; S. Ozawa; S. Sadahiro; S. Yasuda; K. Hirabayashi; T. Nakagohri |
| Repeated radioembolization in advanced liver cancer | 2020 | M. Masthoff; P. Schindler; F. Harders; W. Heindel; C. Wilms; H. H. Schmidt; A. Pascher; L. Stegger; K. Rahbar; M. Wildgruber; M. Köhler |
| Report of the 22nd nationwide follow-up Survey of Primary Liver Cancer in Japan (2012–2013) | 2022 | M. Kudo; N. Izumi; N. Kokudo; M. Sakamoto; S. Shiina; T. Takayama; R. Tateishi; O. Nakashima; T. Murakami; Y. Matsuyama; A. Takahashi; H. Miyata; S. Kubo |
| Resection of hilar cholangiocarcinoma | 1998 | S. M. Strasberg |
| Restoration of liver function in cases of surgical obstructive jaundice after biliary decompression: A longitudinal study | 2021 | U. Singh; S. Chakravarti; A. Jain; S. Arora; S. Lal; P. K. Patnaik |
| Results of pancreatoduodenectomy of a low volume surgeon; a retrospective analysis | 2012 | S. Subramanian; S. Gavini; S. Gopal |
| Rethinking Liver Fibrosis Staging in Patients with Hepatocellular Carcinoma: New Insights from a Large Two-Center Cohort Study | 2022 | W. Xu; B. Li; Z. Yang; J. Li; F. Liu; Y. Liu |
| Retrospective Analysis of the Mass Forming Focal Pancreatitis at the Pancreatic Head after Pancreaticoduodenectomy | 2021 | A. T. Kirac; E. O. Kirimker; M. A. Koc; M. K. Karayalcin; A. Tuzuner |
| Retrospective Study Risk factors for occult metastasis detected by inflammation-based prognostic scores and tumor markers in biliary tract cancer | 2021 | Y. Hashimoto; T. Ajiki; H. Yanagimoto; D. Tsugawa; K. Shinozaki; H. Toyama; M. Kido; T. Fukumoto |
| Rhabdomyosarcoma of the common bile duct: Case report | 2005 | D. Tuǧcu; F. Akici; A. Akçay; Z. Şalcioǧlu; G. Aydoǧan; H. Şen; S. Yeşinel; Z. Önal; M. Gülcan; G. Tireli; M. Önal; G. Tekoǧul; R. Ramazanoǧlu; S. Dervişoǧlu |
| Right Portal Vein Ligation Combined With In Situ Splitting Induces Rapid Left Lateral Liver Lobe Hypertrophy Enabling 2-Staged Extended Right Hepatic Resection in Small-for-Size Settings | 2012 | A. A. Schnitzbauer; S. A. Lang; H. Goessmann; S. Nadalin; J. Baumgart; S. A. Farkas; S. Fichtner-Feigl; T. Lorf; A. Goralcyk; R. Hoerbelt; A. Kroemer; M. Loss; P. Ruemmele; M. N. Scherer; W. Padberg; A. Koenigsrainer; H. Lang; A. Obed; H. J. Schlitt |
| Risk calculator for predicting postoperative pneumonia after gastroenterological surgery based on a national Japanese database | 2019 | Y. Takesue; H. Miyata; M. Gotoh; G. Wakabayashi; H. Konno; M. Mori; H. Kumamaru; T. Ueda; K. Nakajima; M. Uchino; Y. Seto |
| Risk factors for complications after pancreatic head resection | 2004 | U. Adam; F. Makowiec; H. Riediger; W. D. Schareck; S. Benz; U. T. Hopt |
| Risk factors for life-threatening (isgpf grade c) pancreatic fistula following pancreaticoduodenectomy | 2013 | S. H. Tsang; K. S. H. Chok; T. T. Cheung; W. K. Yuen; A. C. Chan; T. C. L. Wong; W. Sharr; R. T. P. Poon; S. T. Fan; C. M. Lo |
| Risk factors for post-hepatectomy liver failure in 80 patients | 2021 | Y. Xing; Z. R. Liu; W. Yu; H. Y. Zhang; M. M. Song |
| Risk factors of ineffective drainage in uncovered self-expandable metal stenting for unresectable malignant hilar biliary strictures | 2018 | K. Takahashi; T. Tsuyuguchi; A. Saiga; T. Horikoshi; Y. xOoka; H. Sugiyama; M. Nakamura; J. Kumagai; M. Yamato; Y. Iino; A. Shingyoji; H. Ohyama; S. Yasui; R. Mikata; Y. Sakai; N. Kato |
| Risk factors of posthepatectomy liver failure for perihilar cholangiocarcinoma | 2020 | J. W. Lee; J. H. Lee; Y. Park; W. Lee; J. Kwon; K. B. Song; D. W. Hwang; S. C. Kim |
| Risk Factors of Postoperative Complications of Pancreatoduodenectomy | 2014 | L. Yu; Q. Huang; F. Xie; X. Lin; C. Liu |
| Risk Factors, Patterns, and Long-Term Survival of Recurrence After Radiofrequency Ablation With or Without Transarterial Chemoembolization for Hepatocellular Carcinoma | 2021 | J. Huang; W. Huang; Y. Guo; M. Cai; J. Zhou; L. Lin; K. Zhu |
| Risk model for severe postoperative complications after total pancreatectomy based on a nationwide clinical database | 2020 | D. Hashimoto; M. Mizuma; H. Kumamaru; H. Miyata; A. Chikamoto; H. Igarashi; T. Itoi; S. Egawa; Y. Kodama; S. Satoi; S. Hamada; K. Mizumoto; H. Yamaue; M. Yamamoto; Y. Kakeji; Y. Seto; H. Baba; M. Unno; T. Shimosegawa; K. Okazaki |
| RISK OF BILIARY SURGERY IN PATIENTS WITH HYPERBILIRUBINEMIA | 1987 | C. A. Pellegrini; P. Allegra; F. S. Bongard; L. W. Way |
| Risk stratification and treatment of primary biliary cholangitis | 2019 | J. Martínez; L. Aguilera; A. Albillos |
| Role of hepatic trisectionectomy in advanced hepatocellular carcinoma | 2017 | J. S. Tsang; K. S. H. Chok; C. M. Lo |
| Role of inflammatory and immune-nutritional prognostic markers in patients undergoing surgical resection for biliary tract cancers | 2021 | S. Conci; T. Campagnaro; E. Danese; E. Lombardo; G. Isa; A. Vitali; I. Marchitelli; F. Bagante; C. Pedrazzani; M. De Bellis; A. Ciangherotti; A. Guglielmi; G. Lippi; A. Ruzzenente |
| Role of octreotide in portal inflow modulation and prophylaxis of posthepatectomy liver failure. prospective clinical study | 2018 | R. Alikhanov; M. Efanov; P. Kim; I. Kazakov; A. Vankovich; S. Iskhagi; V. Subbotin |
| Role of serum total sialic acid in differentiating cholangiocarcinoma from hepatocellular carcinoma | 2003 | P. Kongtawelert; P. Tangkijvanich; S. Ong-Chai; Y. Poovorawan |
| Safe Dissemination of Laparoscopic Liver Resection in 27,146 Cases between 2011 and 2017 from the National Clinical Database of Japan | 2021 | D. Ban; M. Tanabe; H. Kumamaru; H. Nitta; Y. Otsuka; H. Miyata; Y. Kakeji; Y. Kitagawa; H. Kaneko; G. Wakabayashi; H. Yamaue; M. Yamamoto |
| Safe for Mother, Baby, and Graft? Pregnancy After Liver Transplant: A Single-Center Experience | 2022 | J. Rashidi-Alavijeh; A. Frey; A. Hörster; B. P. Nguyen; A. Iannaccone; F. Saner; C. M. Lange; K. Willuweit |
| Safe use of livers from deceased donors older than 70 years in recipients with HCV cirrhosis treated with direct-action antivirals. Retrospective cohort study | 2021 | C. Jiménez-Romero; I. Justo; A. Marcacuzco; V. García; A. Manrique; Á. García-Sesma; J. Calvo; I. Fernández; C. Martín-Arriscado; Ó. Caso |
| Safety and efficacy of chemosaturation in patients with primary and secondary liver tumours: a single-centre experience after 54 treatments | 2017 | M. M. Kirstein; S. Marquardt; N. Jedicke; S. Marhenke; W. Koppert; M. P. Manns; F. Wacker; A. Vogel |
| Safety and Prognosis of Transarterial Chemoembolization for Octogenarians with Hepatocellular Carcinoma | 2019 | H. M. Cheng; T. Tanaka; H. Nishiofuku; Y. Chanoki; K. Horiuchi; T. Masada; S. Tatsumoto; T. Matsumoto; N. Marugami; K. Kichikawa |
| Safety Evaluation of Paclitaxel-Eluting Biliary Metal Stent with Sodium Caprate in Porcine Biliary Tract | 2019 | S. I. Jang; S. Jeong; D. H. Lee; K. Na; S. Yang; D. K. Lee |
| Safety of Conservative Management of Bile Leakage after Hepatectomy with Biliary Reconstruction | 2008 | A. Ferrero; N. Russolillo; L. Vigano; E. Sgotto; R. Lo Tesoriere; M. Amisano; L. Capussotti |
| Safety of Patients with Hepatitis C Virus Treated with Glecaprevir/Pibrentasvir from Clinical Trials and Real-World Cohorts | 2021 | X. Forns; J. J. Feld; D. E. Dylla; S. Pol; K. Chayama; J. Hou; J. Heo; P. Lampertico; A. Brown; M. Bondin; F. Tatsch; M. Burroughs; J. Marcinak; Z. Zhang; A. Emmett; S. C. Gordon; I. M. Jacobson |
| Safety of repeated radioembolizations in patients with advanced primary and secondary liver tumors and progressive disease after first selective internal radiotherapy | 2014 | A. Zarva; K. Mohnike; R. Damm; J. Ruf; R. Seidensticker; G. Ulrich; M. Seidensticker; M. Pech; J. Ricke; H. Amthauer |
| SAFETY PROFILE OF LIVER-DIRECTED, NON-VIRAL GENE DELIVERY BY ENDOSCOPIC-MEDIATED HYDRODYNAMIC INJECTION OF THE BILIARY TRACT IN PIGS | 2020 | Y. Huang; R. Kruse; T. Shum; H. Ding; C. Simsek; J. Izzi; Z. Wang; Y. Ichkhanian; M. I. Itani; E. Liapi; L. Li; F. Selaru; V. Kumbhari |
| Sarcomatous intrahepatic cholangiocarcinoma Case report and literature review | 2018 | N. Zhang; Y. Li; M. Zhao; X. Chang; F. Tian; Q. Qu; X. He |
| Sarcopenia and myosteatosis as prognostic markers in patients with advanced cholangiocarcinoma undergoing palliative treatment | 2021 | M. S. Jördens; L. Wittig; L. Heinrichs; V. Keitel; M. Schulze‐hagen; G. Antoch; W. T. Knoefel; G. Fluegen; T. Luedde; C. Loberg; C. Roderburg; S. H. Loosen |
| Sarcopenia increases the risk of early biliary infection after percutaneous transhepatic biliary stent placement | 2022 | Q. Chen; X. Lu; Z. K. Wang; C. Feng; X. J. Yao; J. H. Guo |
| Sarcopenia is closely associated with pancreatic exocrine insufficiency in patients with pancreatic disease | 2017 | R. Shintakuya; K. Uemura; Y. Murakami; N. Kondo; N. Nakagawa; K. Urabe; K. Okano; K. Awai; T. Higaki; T. Sueda |
| Scoring systems for the management of oncological hepato-pancreato-biliary patients | 2022 | A. W. Coombs; C. Jordan; S. A. Hussain; O. Ghandour |
| Secondary sclerosing cholangitis and hodgkin’s lymphoma | 2015 | S. H. Abedi; M. Ghassami; M. Molaei; Z. Mohsenifar; A. H. M. Alizadeh |
| Second-line Chemotherapy Prolongs Survival in Real World Patients with Advanced Biliary Tract and Gallbladder Cancers: A Multicenter Retrospective Population-based Cohort Study | 2021 | A. Zaidi; N. Chandna; G. Narasimhan; M. Moser; K. Haider; H. Chalchal; J. Shaw; S. Ahmed |
| Segmental Atrophy: A Rare Pseudotumor of the Liver | 2018 | T. Thomas; C. Cezar; A. Laiyemo |
| Selective hemihepatic vascular occlusion versus pringle maneuver in hepatectomy for primary liver cancer | 2017 | M. Li; T. Zhang; L. Wang; B. Li; Y. Ding; C. Zhang; S. He; Z. Yang |
| Selective Internal Radiotherapy Changes the Immune Profiles of Extracellular Vesicles and Their Immune Origin in Patients with Inoperable Cholangiocarcinoma | 2022 | F. Haag; A. Manikkam; D. Kraft; C. Bär; V. Wilke; A. J. Nowak; J. Bertrand; J. Omari; M. Pech; S. Gylstorff; B. Relja |
| Sensitivity of liver parameters in diagnosing liver diseases in rabbits | 2016 | A. Leban-Danzl; K. Hartmann; M. Majzoub-Altweck; W. Hermanns; C. Sauter-Louisa; J. Hein |
| Sepsis in patients with haematological versus solid cancer: A retrospective cohort study | 2021 | R. Bou Chebl; R. Safa; M. Sabra; A. Chami; I. Berbari; S. Jamali; M. Makki; H. Tamim; G. Abou Dagher |
| Serotonin: Is it a marker for the diagnosis of hepatocellular carcinoma in cirrhotic patients? | 2013 | H. A. A. Moety; D. A. Maharem; S. H. Gomaa |
| Serum albumin predicts survival in patients with hilar cholangiocarcinoma | 2017 | A. Waghray; A. Sobotka; C. R. Marrero; B. Estfan; F. Aucejo; K. V. N. Menon |
| Serum albumin to globulin ratio as an indicator for the prognosis for cholangiocarcinoma | 2016 | Q. Lin; Z. H. Lin; J. Chen; M. Huang; X. Li; J. R. Jiang; M. Dong; T. T. Wang; D. H. Wu; X. K. Ma |
| Serum and biliary MMP-9 and TIMP-1 concentrations in the diagnosis of cholangiocarcinoma | 2015 | A. T. Iİnce; K. Yıldız; V. Gangarapu; Y. Kayar; B. Baysal; O. Karatepe; A. S. Kemik; H. Şentürk |
| Serum copeptin level is a biomarker associated with ascites retention and the formation of a portosystemic shunt in chronic liver disease | 2021 | R. Shigefuku; M. Iwasa; A. Eguchi; Y. Tamai; K. Yoshikawa; R. Sugimoto; Y. Takei |
| Serum IgG4 cut-off of 70 mg/dL is associated with a shorter time to cirrhosis decompensation and liver transplantation in primary sclerosing cholangitis patients | 2022 | F. Peerani; L. Du; E. Lytvyak; V. G. Bain; A. L. Mason; R. J. Bailey; A. J. Montano-Loza |
| Serum liver enzymes serve as prognostic factors in patients with intrahepatic cholangiocarcinoma | 2017 | C. Zhang; H. Wang; Z. Ning; L. Xu; L. Zhuang; P. Wang; Z. Meng |
| Serum metabolomic and lipoprotein profiling of pancreatic ductal adenocarcinoma patients of african ancestry | 2021 | N. Elebo; J. Omoshoro-Jones; P. N. Fru; J. Devar; C. De Wet van Zyl; B. C. Vorster; M. Smith; S. Cacciatore; L. F. Zerbini; G. Candy; E. E. Nweke |
| Serum mucin 3A as a potential biomarker for extrahepatic cholangiocarcinoma | 2020 | J. Wang; H. Zhou; Y. Wang; H. Huang; J. Yang; W. Gu; X. Zhang; J. Yang |
| Serum nutritional markers and faecal elastase-1 testing in biliopancreatic cancer patients | 2019 | D. Oyón Lara; M. Rullán Iriarte; J. M. Urman Fernández; M. Casi; D. Ruiz-Clavijo García; B. González de la Higuera Carnicer; F. Bolado Concejo |
| Serum unconjugated primary and secondary bile acids in patients with cholangiocarcinoma and hepatocellular carcinoma | 1990 | S. Changbumrung; R. Tungtrongchitr; P. Migasena; S. Chamroenngan |
| Short-Term outcomes after pancreatoduodenectomy in octogenarians: Multicentre case-control study | 2022 | J. A. Attard; B. Al-Sarireh; R. H. Bhogal; A. Farrugia; G. Fusai; S. Harper; C. Hidalgo-Salinas; A. Jah; G. Marangoni; M. Mortimer; M. Pizanias; A. Prachialias; K. J. Roberts; C. Sew Hee; F. Soggiu; P. Srinivasan; N. A. Chatzizacharias |
| Short-term Outcomes of difficult" Laparoscopic Liver Resection at Specialized Centers: Report from INSTALL (International Survey on Technical Aspects of Laparoscopic Liver Resection)-2 on 4478 Patients" | 2022 | S. Ibuki; T. Hibi; M. Tanabe; D. A. Geller; D. Cherqui; G. Wakabayashi |
| Short-term outcomes of paediatric liver transplant recipients after transition to Adult Healthcare Service | 2018 | A. Ferrarese; G. Germani; S. Lazzaro; M. Cananzi; F. P. Russo; M. Senzolo; M. Gambato; A. Zanetto; U. Cillo; E. Gringeri; G. Perilongo; P. Burra |
| Short-term prognostic factors for primary sclerosing cholangitis | 2015 | T. Watanabe; K. Hirano; M. Tada; H. Isayama; S. Mizuno; T. Arizumi; N. Toda; Y. Sugawara; N. Kokudo; K. Koike |
| Should all cases of extra-hepatic cholestasis be operated? | 1985 | C. Meyer; P. Karydakis; M. Safioleas; L. F. Hollender |
| Significance of serum ferritin as a prognostic factor in advanced hepatobilia cancer patients treated with Korean medicine: a retrospective cohort study | 2018 | A. Song; W. Eo; S. Kim; B. Shim; S. Lee |
| Simultaneous pancreaticoduodenectomy and liver transplantation for biliary atresia complicated by choledochal cyst | 2021 | Y. Luo; D. Zhao; T. Zhou; J. Zhu; J. Zhang; Q. Xia |
| Single hepatocellular carcinoma smaller than 2 cm: Are ethanol injection and radiofrequency ablation equally effective? | 2015 | M. Pompili; N. De Matthaeis; A. Saviano; I. De Sio; G. Francica; F. Brunello; A. Cantamessa; A. Giorgio; U. Scognamiglio; F. Fornari; F. Giangregorio; F. Piscaglia; S. Gualandi; E. Caturelli; P. Roselli; L. Riccardi; G. L. Rapaccini |
| Single-agent gemcitabine for biliary tract cancers: Study outcomes and systematic review of the literature | 2006 | T. Kiba; T. Nishimura; S. Matsumoto; E. Hatano; A. Mori; S. Yasumi; R. Doi; I. Ikai; T. Kitano; T. Nishimura; K. Yoshikawa; H. Ishiguro; K. Yanagihara; E. Doi; S. Teramukai; M. Fukushima |
| SIRT in primary and metastatic liver tumors | 2011 | M. Hoffmann |
| Skeletal muscle volume and intramuscular adipose tissue are prognostic predictors of postoperative complications after hepatic resection | 2018 | N. Harimoto; H. Hoshino; R. Muranushi; K. Hagiwara; T. Yamanaka; N. Ishii; M. Tsukagoshi; T. Igarashi; A. Watanabe; N. Kubo; K. Araki; K. Shirabe |
| Small cell lung cancer presenting as painful obstructive jaundice | 2016 | J. Scoon; Y. C. Yeh; A. E. Sagar; R. Lodato |
| Small duct primary sclerosing cholangitis: Ten years of follow up | 2006 | C. Guma; L. Viola; M. Thome; G. Ahumaran; A. Facelli; E. Alvarez |
| Socioeconomic and demographic factors in patients with periampullary cancer | 2021 | M. Botejue; R. Zackria; S. Kubomoto |
| Solitary liver lesions are not always HCC: Metastasis from an uncommon site mimicking a primary liver lesion | 2020 | M. T. Al-Qaisi; A. J. Gomez; M. Batheja |
| SOURCE-PANC: A prediction model for patients with metastatic pancreatic ductal adenocarcinoma based on nationwide population-based data | 2021 | H. G. van den Boorn; W. P. M. Dijksterhuis; L. G. M. van der Geest; J. de Vos-Geelen; M. G. Besselink; J. W. Wilmink; M. G. H. van Oijen; H. W. M. van Laarhoven |
| Squamous cell carcinoma of the pancreas with liver metastasis: A case report | 2008 | Q. P. Chen; K. Ou; Q. H. Guan; F. Zhang |
| Staging laparoscopy among three subtypes of extra-hepatic biliary malignancy: a 15-year experience from 10 institutions | 2019 | J. T. Davidson; L. X. Jin; B. Krasnick; C. G. Ethun; T. M. Pawlik; G. A. Poultsides; K. Idrees; S. M. Weber; R. C. G. Martin; P. Shen; I. Hatzaras; S. K. Maithel; R. C. Fields |
| Stenting metastatic bile duct obstruction (BDO): The importance of liver function parameters | 2015 | M. Bergamino Sirven; R. Montal; M. Galan; J. Gornals Soler; E. Escalante Porrua; V. Navarro Perez; M. Marin Melia |
| Subcentimeter Nodules with Diagnostic Hallmarks of Hepatocellular Carcinoma: Comparison of Pathological Features and Survival Outcomes with Nodules Measuring 1–2 cm | 2023 | P. Huang; X. Ni; C. Zhou; Z. Shi; F. Wu; Y. Xiao; C. Yang; M. Zeng |
| Successful resection of a centrally located primary hepatic neuroendocrine tumor | 2020 | A. Alakeel; A. Alshamrani; A. Alharbi; A. Mubarah; H. Alshurafa; M. Aldayel |
| Superselective Transarterial Chemoembolization for Unresectable or “Ablation Unsuitable” Hepatocellular Carcinoma in the Caudate Lobe: A Real World, Single-Center Retrospective Study | 2021 | L. Yan; Y. Ren; K. Qian; X. Kan; H. Zhang; L. Chen; B. Liang; C. Zheng |
| Surgery of hepatocellular carcinoma complicated with cancer thrombi in bile duct: Efficacy for criteria for different therapy modalities | 2009 | L. Xiangji; T. Weifeng; Y. Bin; L. Chen; J. Xiaoqing; Z. Baihe; S. Feng; W. Mengchao |
| Surgical Administration of Indocyanine Green in Hepatectomy for Improved Bile Leakage Detection | 2022 | T. Hanaki; K. Goto; M. Morimoto; Y. Murakami; T. Matsunaga; M. Yamamoto; N. Tokuyasu; T. Sakamoto; T. Hasegawa; Y. Fujiwara |
| Surgical and radiological decompression in malignant biliary obstruction: A retrospective study using multivariate risk factor analysis | 1984 | D. Bonnel; J. T. Ferrucci Jr; P. R. Mueller |
| Surgical Loupe at 4.0× Magnification in Pancreaticoduodenectomy—Does It Affect the Surgical Outcomes? A Propensity Score–Matched Study | 2019 | M. El Shobary; A. El Nakeeb; A. Sultan; M. A. E. W. Ali; M. El Dosoky; A. Shehta; H. Ezzat; A. M. Elsabbagh |
| Surgical Outcomes for Hepatocellular Carcinoma in Patients with Child-Pugh Class B: a Retrospective Multicenter Study | 2023 | S. Tanaka; T. Noda; K. Komeda; H. Kosaka; H. Iida; M. Ueno; D. Hokuto; H. Ikoma; T. Nakai; D. Kabata; H. Shinkawa; S. Kobayashi; F. Hirokawa; H. Mori; S. Hayami; R. Morimura; M. Matsumoto; T. Ishizawa; S. Kubo; M. Kaibori |
| Surveillance of primary sclerosing cholangitis with ERC and brush cytology: risk factors for cholangiocarcinoma | 2017 | S. Boyd; H. Mustonen; A. Tenca; K. Jokelainen; J. Arola; M. A. Färkkilä |
| Survival and Toxicities after Yttrium-90 Transarterial Radioembolization of Cholangiocarcinoma in the RESiN Registry | 2022 | T. J. Robinson; L. Du; L. Matsuoka; D. Y. Sze; A. S. Kennedy; R. T. Gandhi; B. E. Kouri; Z. S. Collins; N. Kokabi; C. J. Grilli; E. A. Wang; J. S. Lee; D. B. Brown |
| Sustained multiple organ ischaemia after transarterialchemoembolization with drug-eluting beadsfor hepatocellular carcinoma | 2018 | Y. W. Kim; J. H. Kwon; S. W. Nam; J. W. Jang; H. S. Jung; Y. R. Shin; E. S. Park; D. J. Shim |
| Synchronous occurrence of gastrointestinal stromal tumor, pancreatic intraductal papillary mucinous neoplasm, and intrahepatic cholangiocarcinoma: Case report | 2022 | Q. Hou; W. Zhang; J. Niu; M. Tian; J. Liu; L. Cui; Y. Li |
| Syphting Through the Evidence: A Very Distinctive Cause of Neonatal Jaundice | 2019 | R. M. Ruiz; D. M. Bass |
| Systemic treatments with tyrosine kinase inhibitors and platinum-based chemotherapy in patients with unresectable or metastatic hepato-cholangiocarcinoma | 2022 | E. Gigante; C. Hobeika; B. Le Bail; V. Paradis; D. Tougeron; M. Lequoy; M. Bouattour; J.-F. Blanc; N. Ganne-Carrie; H. Tran; C. Hollande; M. Allaire; G. Amaddeo; H. Regnault; P. Vigneron; M. Ronot; L. Elkrief; G. Verset; E. Trepo; A. Zaanan; M. Ziol; M. Ningarhari; J. Calderaro; J. Edeline; J. C. Nault |
| T lymphocyte function in patients with malignant biliary obstruction | 1994 | S. T. Fan; C. M. Lo; E. C. S. Lai; W. C. Yu; J. Wong |
| Tc-99m-MAA lung shunt fraction before Y-90 radioembolization is low among patients with non-hepatocellular carcinoma malignancies | 2019 | M. Elsayed; J. G. Martin; A. Dabrowiecki; D. T. Goldman; R. Faraj; J. T. McMahon; N. Kokabi; R. Duszak; J. Newsome; Z. L. Bercu |
| Tenofovir vs. Entecavir on Outcomes of Hepatitis B Virus-Related Hepatocellular Carcinoma after Radiofrequency Ablation | 2022 | Z. Hu; H. Zeng; J. Hou; J. Wang; L. Xu; Y. Zhang; M. Chen; Z. Zhou |
| TFEB regulates murine liver cell fate during development and regeneration | 2020 | N. Pastore; T. Huynh; N. J. Herz; A. Calcagni’; T. J. Klisch; L. Brunetti; K. H. Kim; M. De Giorgi; A. Hurley; A. Carissimo; M. Mutarelli; N. Aleksieva; L. D’Orsi; W. R. Lagor; D. D. Moore; C. Settembre; M. J. Finegold; S. J. Forbes; A. Ballabio |
| Thalidomide induced acute cholestatic hepatitis | 2011 | F. Vilas-Boas; R. Gonc¸alves; M. S. Simões; J. Lopes; G. Macedo |
| The Addition of Transarterial Chemoembolization to Palliative Chemotherapy Extends Survival in Intrahepatic Cholangiocarcinoma | 2021 | S. J. Gairing; F. Thol; L. Mueller; F. Hahn; T. Thomaidis; C. Czauderna; F. Bartsch; M. B. Pitton; J. U. Marquardt; M.-A. Worns; P. R. Galle; M. Moehler; A. Weinmann; R. Kloeckner; F. Foerster |
| The ALBI score: From liver function in patients with HCC to a general measure of liver function | 2022 | H. Toyoda; P. J. Johnson |
| The allure of a first diagnosis | 2012 | V. Wong |
| The ALPPS procedure for hepatocellular carcinoma larger than 10 centimeters | 2016 | O. J. M. Torres; R. R. Vasques; T. H. S. Silva; M. E. L. Castelo-Branco; C. C. S. Torres |
| The Application of Selective Hepatic Inflow Vascular Occlusion with Anterior Approach in Liver Resection: Effectiveness in Managing Major Complications and Long-Term Survival | 2021 | K. V. Ninh; N. Q. Nguyen; S. H. Trinh; A. G. Pham; T. N. H. Doan |
| The Association of Low Level of Both Vitamin a and Retinol Binding Protein With the Liver Complications in Patients With Primary Sclerosing Cholangitis | 2010 | S. Treeprasertsuk; P. L. Jansen; K. V. Kowdley; V. A. Luketic; M. E. Harrison; T. M. McCashland; A. Befeler; D. M. Harnois; R. A. Jorgensen; J. C. Keach; J. Schmoll; T. Hoskin; P. Thapa; F. Enders; K. D. Lindor |
| THE ASSOCIATION OF UDCA TREATMENT WITH LONG-TERM OUTCOME AND BILIARY TRACT CANCER IN PATIENTS WITH PRIMARY SCLEROSING CHOLANGITIS | 2020 | T. Arizumi; S. Tazuma; T. Nakazawa; H. Isayama; T. Tsuyuguchi; H. Takikawa; A. Tanaka; P. S. C. S. G. J. Japan |
| The chronic cholestasis enigma in adults | 2002 | R. Reshef; W. Sbeit; J. Lachter |
| The clinical characteristics and prognostic factors of combined hepatocellular carcinoma and cholangio-carcinoma, hepatocellular carcinoma and intrahepatic cholangiocarcinoma after surgical resection: A propensity score matching analysis | 2020 | Y. Tang; L. Wang; F. Teng; T. Zhang; Y. Zhao; Z. Chen |
| The clinical implication of gamma-glutamyl transpeptidase in COVID-19 | 2021 | J. Liu; C. Yu; Q. Yang; X. Yuan; F. Yang; P. Li; G. Chen; W. Liang; Y. Yang |
| The clinical presentation and prognostic factors for intrahepatic and extrahepatic cholangiocarcinoma in a tertiary care centre | 2010 | A. G. Singal; M. O. Rakoski; R. Salgia; S. Pelletier; T. H. Welling; R. J. Fontana; A. S. Lok; J. A. Marrero |
| The Comprehensive Analysis of Efficacy and Safety of CalliSpheres (R) Drug-Eluting Beads Transarterial Chemoembolization in 367 Liver Cancer Patients: A Multiple-Center, Cohort Study | 2020 | Z. Peng; G. Cao; Q. Hou; L. Li; S. Ying; J. Sun; G. Zhou; J. Zhou; X. Zhang; W. Ji; Z. Yu; T. Li; D. Zhu; W. Hu; J. Ji; H. Du; C. Shi; X. Guo; J. Fang; J. Han; W. Gu; X. Xie; Z. Sun; H. Xu; X. Wu; T. Hu; J. Huang; H. Hu; J. Zheng; J. Luo; Y. Chen; W. Yu; G. Shao |
| The comprehensive analysis of efficacy and safety of CalliSpheres® drug-eluting beads transarterial chemoembolization in 367 patients with liver cancer: A multiple-center, prospective cohort study (CTILC study) | 2018 | Z. Peng; G. Zhou; W. Yu; G. Shao |
| The curious case of the vanishing ducts | 2016 | M. Gao; T. Wong |
| The development of a predictive risk model on post-ablation hemobilia: A multicenter matched case–control study | 2021 | B. Liu; H. Li; J. Guo; Y. Duan; C. Li; J. Chen; J. Zheng; W. Li |
| The development of broncho-biliary fistula after treatment for hepatocellular carcinoma: a report of two cases | 2021 | S. Takakusagi; T. Hoshino; H. Takagi; A. Naganuma; Y. Yokoyama; K. Kizawa; K. Marubashi; T. Kosone; A. Watanabe; N. Kubo; K. Araki; N. Harimoto; K. Shirabe; S. Nobusawa; D. Zennyoji; T. Shimizu; K. Sato; S. Kakizaki; T. Uraoka |
| The effect of nutritional status on outcome of hospitalization in paediatric liver disease patients | 2016 | Y. Mansi; S. Abdelghaffar; S. Sayed; H. El-Karaksy |
| The efficacy of interventional therapy for hilar cholangiocarcinoma: A comparison between unilateral and bilateral biliary duct drainage | 2009 | F. L. Yang; H. Y. Su; B. Feng; H. B. Shao; K. Xu |
| The efficacy of portal vein embolization prior to right extended hemihepatectomy for hilar cholangiocellular carcinoma: A retrospective cohort study | 2011 | Y. K. Hong; S. B. Choi; K. H. Lee; S. W. Park; Y. N. Park; J. S. Choi; W. J. Lee; J. B. Chung; K. S. Kim |
| The first case of cholecytitis caused by Aggregatibacter kilianii in Korea | 2022 | D. Yang; C. K. Kim; J. Park; N. Y. Song; Y. Lee; W. Lee |
| The identification of risk factors and their application to the management of obstructive jaundice | 1980 | D. R. Hunt |
| The impact of alanyl-glutamine on clinical safety, nitrogen balance, intestinal permeability, and clinical outcome in postoperative patients: A randomized, double-blind, controlled study of 120 patients | 1999 | Z. M. Jiang; J. D. Cao; X. G. Zhu; W. X. Zhao; J. C. Yu; E. L. Ma; X. R. Wang; M. W. Zhu; H. Shu; Y. W. Liu |
| The impact of preoperative biliary drainage on postoperative outcomes in patients with malignant obstructive jaundice: a retrospective analysis of 290 consecutive cases at a single medical center | 2022 | Z. Gao; J. Wang; S. Shen; X. Bo; T. Suo; X. Ni; H. Liu; L. Huang; H. Liu |
| The impact of the albumin-bilirubin (ALBI) score in gallbladder cancer prognosis | 2022 | J. Nogueiro; T. Costa; S. S. Hugo; M. Aral; R. B. Melo; L. Graça; E. Barbosa |
| The indications for and timing of liver transplantation | 1999 | L. K. Schluger; F. M. Klion |
| The influence of enteral nutrition in postoperative patients with poor liver function | 2003 | Q. G. Hu; Q. C. Zheng |
| The klatskin tumor that wasn't: An unusual presentation of sarcoidosis | 2016 | P. D. Farooq; D. R. Potosky |
| The migration route of Fasciola into the liver | 2018 | I. G. Lockart; A. Das; N. Merrett; M. T. Levy |
| The Necessity of Dissection of No. 14 Lymph Nodes to Patients With Pancreatic Ductal Adenocarcinoma Based on the Embryonic Development of the Head of the Pancreas | 2020 | L. Qian; J. Xie; Z. Xu; X. Deng; H. Chen; C. Peng; H. Li; W. Chai; J. Xie; W. Wang; B. Shen |
| The need for liver transplant in a sample of Iraqi children with chronic liver disease | 2021 | R. F. Thejeal; A. R. Bahlol; R. J. Noori |
| The pathology of jaundice-related renal insufficiency: cholemic nephrosis revisited | 2006 | M. G. H. Betjes; I. Bajema |
| The phes score does not correlate with blood ammonia levels, circulating endotoxins or markers of systemic inflammation | 2017 | N. Kimer; L. L. Gluud; J. S. Pedersen; J. Tavenier; S. Møller; F. Bendtsen; M. Y. Morgan |
| The predictive role of tenascin-C and cellular communication network factor 3 (CCN3) in post hepatectomy liver failure in a rat model and 50 patients following partial hepatectomy | 2019 | H. Li; X. Ge; K. Pan; M. Sui; H. Cai; C. Cui; C. Li; S. Lu |
| The prevalence and significance of autoantibodies in patients with nonalcholoic fatty liver disease | 2008 | K. Rao; M. Ahmad; A. Samanta; K. Klein; B. Koneru; A. Fisher; D. Wilson; A. De La Torre |
| The prevalence, incidence and natural history of primary sclerosing cholangitis in an ethnically diverse population | 2011 | E. Toy; S. Balasubramanian; C. Selmi; C.-S. Li; C. L. Bowlus |
| The prognostic factors for primary sclerosing cholangitis, including oxidative stress markers | 2019 | A. Oyama; A. Takaki; T. Adachi; N. Wada; M. Sakata; T. Yasunaka; H. Onishi; H. Shiraha; H. Okada |
| The prognostic impact of lymphocyte-to-C-reactive protein score in patients undergoing surgical resection for intrahepatic cholangiocarcinoma: A comparative study of major representative inflammatory / immunonutritional markers | 2021 | D. Noguchi; N. Kuriyama; Y. Nakagawa; K. Maeda; T. Shinkai; K. Gyoten; A. Hayasaki; T. Fujii; Y. Iizawa; A. Tanemura; Y. Murata; M. Kishiwada; H. Sakurai; S. Mizuno |
| The prognostic impact of preoperative body composition in perihilar and intrahepatic cholangiocarcinoma | 2022 | I. Lurje; Z. Czigany; S. Eischet; J. Bednarsch; T. F. Ulmer; P. Isfort; P. Strnad; C. Trautwein; F. Tacke; U. P. Neumann; G. Lurje |
| The prognostic nutritional index predicts survival and response to first-line chemotherapy in advanced biliary cancer | 2020 | M. Salati; R. Filippi; C. Vivaldi; F. Caputo; F. Leone; F. Salani; K. Cerma; M. Aglietta; L. Fornaro; E. Sperti; M. Di Maio; C. Ortega; E. Fenocchio; P. Lombardi; C. Cagnazzo; I. Depetris; F. Gelsomino; A. Spallanzani; D. Santini; N. Silvestris; G. Aprile; G. Roviello; M. Scartozzi; S. Cascinu; A. Casadei-Gardini |
| The prognostic significance of pretreatment serum γ-glutamyltranspeptidase in primary liver cancer: A meta-analysis and systematic review | 2018 | Y. Ou; J. Huang; L. Yang |
| The proof is in the (liver) parenchyma: Primary hepatic presentation of multiple myeloma and amyloidosis | 2016 | C. Ihunnah; M. S. DiSiena; M. Einstein |
| The ratio of abdominal depth to body mass index is a preoperative predictor of postoperative complications after laparoscopic pancreaticoduodenectomy: a retrospective propensity score matched analysis | 2021 | H. Wang; J. Jin; F. Zhu; F. Peng; M. Wang; R. Qin |
| The Relationship between the Number of Ports and Surgical Outcomes in Laparoscopic Hepatectomy | 2020 | Y. Inoue; K. Kitada; K. Fujii; S. Kagota; A. Tomioka; T. Yamaguchi; K. Yokohama; H. Ohama; H. Hamamoto; M. Ishii; W. Osumi; Y. Tsuchimoto; T. Terazawa; T. Ogura; S. Masubuchi; M. Yamamoto; A. Imoto; A. Asai; K. Komeda; S. Fukunishi; F. Hirokawa; M. Goto; K. Tanaka; J. Okuda; K. Higuchi; K. Uchiyama |
| THE ROLE OF LIVER BIOPSY IN THE DIAGNOSIS AND PROGNOSIS OF PEDIATRIC PRIMARY SCLEROSING CHOLANGITIS AND AUTOIMMUNE HEPATITIS OVERLAP | 2021 | J. Stevens; N. A. Gupta; M. DiGuglielmo; K. N. Furuya; J. Hochberg; S. P. Horslen; N. Kerkar; B. G. Koot; K. M. Loomes; C. L. Mack; M. Martinez; A. Miethke; T. A. Miloh; S. Mohammad; A. J. Montano-Loza; N. Ovchinsky; E. R. Perito; G. S. Rao; A. Ricciuto; P. Sathya; K. Schwarz; U. Shah; N. Soufi; M. E. Tessier; P. L. Valentino; B. Vitola; M. Deneau |
| The role of resolvin D1 in the differential diagnosis of the cholangiocarcinoma and benign biliary diseases | 2020 | Ö. Gül-Utku; E. Karatay; B. Ergül; Ü. Kisa; Z. Erdin; D. Oguz |
| The role of volumetric assessment in predicting the success of percutaneous transhepatic biliary drainage in inoperable Klatskin tumours | 2022 | G. K. Bahadir; M. Özdemir |
| The Safety and Feasibility of Three-Dimensional Visualization Technology Assisted Right Posterior Lobe Allied with Part of v and VIII Sectionectomy for Right Hepatic Malignancy Therapy | 2018 | M. Hu; H. Hu; W. Cai; Z. Mo; N. Xiang; J. Yang; C. Fang |
| The Sclerosing Cholangitis Outcomes in Pediatrics (SCOPE) Index: A Prognostic Tool for Children | 2021 | M. R. Deneau; C. Mack; E. R. Perito; A. Ricciuto; P. L. Valentino; M. Amin; A. Z. Amir; M. Aumar; M. Auth; A. Broderick; M. DiGuglielmo; L. G. Draijer; E. D. T. Fagundes; W. El-Matary; F. Ferrari; K. N. Furuya; N. Gupta; J. T. Hochberg; M. Homan; S. Horslen; R. Iorio; M. K. Jensen; M. M. Jonas; B. M. Kamath; N. Kerkar; K. M. Kim; K.-L. Kolho; B. G. P. Koot; T. J. Laborda; C. K. Lee; K. M. Loomes; M. Martinez; A. Miethke; T. Miloh; D. Mogul; S. Mohammad; P. Mohan; S. Moroz; N. Ovchinsky; S. Palle; A. Papadopoulou; G. Rao; A. R. Ferreira; P. Sathya; K. B. Schwarz; U. Shah; E. Shteyer; R. Singh; V. Smolka; N. Soufi; A. Tanaka; R. Varier; B. Vitola; M. Woynarowski; M. Zerofsky; A. Zizzo; S. L. Guthery |
| The state of immunotherapy in hepatobiliary cancers | 2021 | F. Z. Ilyas; J. D. Beane; T. M. Pawlik |
| The successful treatment of bile cast nephropathy with plasma exchange | 2020 | S. Reddy; T. Kinard; M. Ryan; L. F. Thomas |
| The value of gadoxetate disodium-enhanced MR imaging for predicting posthepatectomy liver failure after major hepatic resection: A preliminary study | 2011 | S. H. Cho; U. R. Kang; J. D. Kim; Y. S. Han; D. L. Choi |
| Therapeutic Efficacy of Percutaneous Radiofrequency Ablation versus Microwave Ablation for Hepatocellular Carcinoma | 2013 | L. Zhang; N. Wang; Q. Shen; W. Cheng; G. J. Qian |
| Therapy of the refractory ascites: Total paracentesis vs. TIPS | 2016 | V. La Mura; F. Salerno |
| Three-day postoperative antibiotics reduces post-hepatectomy infection rate in hepatitis B virus-related hepatocellular carcinoma | 2021 | Z. Chen; H. Jiang; Y. Wang; R. Liang; L. Xu; J. Lai; J. Shen; J. Li; D. Li; S. Li; K. Lei; Q. Zhou; B. Peng; H. Peng; S. Peng; M. Kuang |
| Total Bilirubin Level as a Predictor of Suboptimal Image Quality of the Hepatobiliary Phase of Gadoxetic Acid-Enhanced MRI in Patients with Extrahepatic Bile Duct Cancer | 2022 | J. A. Hwang; J. H. Min; S. H. Kim; S. Y. Choi; J. E. Lee; J. Y. Moon |
| Total laparoscopic pancreaticoduodenectomy with left posterior superior mesenteric artery first-approach and plexus-preserving circumferential lymphadenectomy: step-by-step technique with a surgical case report (with video) | 2022 | T. Khiem; H. Hoi; T. Hiep; K. Khue; V. Duy; Y. Inoue; H. Son; D. Dung |
| Total serum bile acid as a potential marker for the diagnosis of cholangiocarcinoma without jaundice | 2015 | S. Sombattheera; T. Proungvitaya; T. Limpaiboon; S. Wongkham; C. Wongkham; V. Luvira; S. Proungvitaya |
| TOTAL VASCULAR EXCLUSION FOR MAJOR HEPATECTOMY IN PATIENTS WITH ABNORMAL LIVER PARENCHYMA | 1995 | J. Emond; M. E. Wachs; J. F. Renz; S. Kelley; H. Harris; J. P. Roberts; N. L. Ascher; R. C. Lim |
| Totally laparoscopic versus open pancreaticoduodenectomy: A propensity score matching analysis of short-term outcomes | 2021 | M. Mazzola; A. Giani; J. Crippa; L. Morini; A. Zironda; C. L. Bertoglio; P. De Martini; C. Magistro; G. Ferrari |
| Toxicologic effects of 28-day dietary exposure to the flame retardant 1,2-dibromo-4-(1,2-dibromoethyl)-cyclohexane (TBECH) in F344 rats | 2017 | I. H. A. Curran; V. Liston; A. Nunnikhoven; L. Coady; C. Armstrong; D. E. Lefebvre; G. S. Bondy; D. Caldwell; M. J. S. Scuby; P. Pantazopoulos; D. F. K. Rawn |
| Transarterial 90yttrium radioembolisation | 2021 | C. Mosconi; R. Golfieri |
| Transarterial Chemoembolization for Hepatocellular Carcinoma in Clinical Practice: Temporal Trends and Survival Outcomes of an Iterative Treatment | 2022 | F. Pelizzaro; S. Haxhi; B. Penzo; A. Vitale; E. G. Giannini; V. Sansone; G. L. Rapaccini; M. Di Marco; E. Caturelli; D. Magalotti; R. Sacco; C. Celsa; C. Campani; A. Mega; M. Guarino; A. Gasbarrini; G. Svegliati-Baroni; F. G. Foschi; A. Olivani; A. Masotto; G. Nardone; G. Raimondo; F. Azzaroli; G. Vidili; M. R. Brunetto; F. Trevisani; F. Farinati |
| Transarterial chemoembolization versus supportive therapy in the palliative treatment of unresectable intrahepatic cholangiocarcinoma | 2011 | S. Y. Park; J. H. Kim; H. J. Yoon; I. S. Lee; H. K. Yoon; K. P. Kim |
| Transarterial radioembolization of hepatocellular carcinoma, liver-dominant hepatic colorectal cancer metastases, and cholangiocarcinoma using yttrium90 microspheres: Eight-year single-center real-life experience | 2021 | J. Pellegrinelli; O. Chevallier; S. Manfredi; I. Dygai-Cochet; C. Tabouret-Viaud; G. Nodari; F. Ghiringhelli; J. M. Riedinger; R. Popoff; J. M. Vrigneaud; A. Cochet; S. Aho; M. Latournerie; R. Loffroy |
| Transarterial Yttrium-90 Radioembolization in Intrahepatic Cholangiocarcinoma Patients: Outcome Assessment Applying a Prognostic Score | 2022 | I. Schatka; H. V. Jochens; J. M. M. Rogasch; T. C. Walter-Rittel; U. Pelzer; J. Benckert; J. Graef; F. W. Feldhaus; B. Gebauer; H. Amthauer |
| Transcatheter arterial chemo-lipiodol infusion for unresectable hepatocellular carcinoma in 96 high-risk patients | 2010 | H. J. Yoon; J. H. Kim; K. A. Kim; I. S. Lee; G. Y. Ko; H. Y. Song; D. I. Gwon |
| Treatment and prognosis of hepatocellular carcinoma: A population based study in France | 2008 | F. Borie; A. M. Bouvier; A. Herrero; J. Faivre; G. Launoy; P. Delafosse; M. Velten; A. Buemi; J. Peng; P. Grosclaude; B. Trétarre |
| Treatment modification of yttrium-90 radioembolization based on quantitative positron emission tomography/CT imaging | 2013 | T. T. Chang; A. C. Bourgeois; A. M. Balius; A. S. Pasciak |
| Treatment of Antibody-Mediated Post-Transplant Autoimmune-like Hepatitis with Daratumumab | 2021 | R. Epperly; T. Santiago; C. Morin; K. Patton; J. Deyo; B. M. Triplett; A. Sharma |
| Treatment of hepatic tumour with 166HO-spheres selective internal therapy (SIRT): Preliminary experience at National Cancer Institute of Milan | 2019 | M. Maccauro; C. Spreafico; S. Bhoori; C. Chiesa; T. Cascella; G. Aliberti; A. Marchiano; E. Seregni; V. Mazzaferro |
| Treatment of non-hepatocellular carcinoma liver disease with Yttrium-90: Alberta Canadian Registry | 2013 | R. Thomas; R. J. Owen |
| Treatment of severe refractory pruritus with fractionated plasma separation and adsorption (Prometheus((R))) | 2006 | K. Rifai; C. Hafer; J. Rosenau; C. Athmann; H. Haller; M. P. Manns; D. Fliser |
| Treatment Outcomes and Prognostic Factors for Intrahepatic Cholangiocarcinoma Single Center Experience | 2011 | R. Dhanasekaran; A. W. Hemming; D. R. Nelson; C. Soldevila-Pico; R. J. Firpi; G. Morelli; V. Clark; T. J. George; Z. Robert; R. Cabrera |
| Treatment strategy for successful hepatic resection of icteric liver | 2018 | K. Yada; Y. Morine; H. Ishibashi; H. Mori; M. Shimada |
| Tubulopapillary adenoma of the gallbladder accompanied by bile duct tumor thrombus | 2014 | K. Yamamoto; F. Yamamoto; A. Maeda; H. Igimi; M. Yamamoto; R. Yamaguchi; Y. Yamashita |
| Twenty years of radiation therapy of unresectable intrahepatic cholangiocarinoma: Internal or external? A systematic review and meta-analysis | 2021 | Q. Yu; C. Liu; A. Pillai; O. Ahmed |
| Two elder cases of hepatocellular carcinoma adjacent to intrahepatic vessels successfully treated by carbon ion radiotherapy | 2020 | S. Takakusagi; H. Takagi; K. Shibuya; T. Kosone; K. Sato; S. Kakizaki; T. Ohno; T. Uraoka |
| Ulcerative colitis and primary sclerosing cholangitis: A fatal duo | 2013 | N. A. Gogela; M. S. Borkum; M. Locketz; M. Sonderup; S. R. Thomson |
| Ultrasonographic evaluation of patients with abnormal liver function tests in the emergency department | 2022 | B. U. Vardar; C. S. Dupuis; A. J. Goldstein; Z. Vardar; Y. H. Kim |
| Ultrasound-guided percutaneous microwave ablation versus surgical resection for recurrent intrahepatic cholangiocarcinoma: intermediate-term results | 2019 | C. Xu; L. Li; W. Xu; C. Du; L. Yang; J. Tong; Y. Yi |
| Unconventional extrahepatic neovascularization after transplant hepatic artery thrombosis: A case report | 2013 | L. Casadaban; A. Parvinian; I. G. Tzvetanov; H. Jeon; J. Oberholzer; E. Benedetti; J. T. Bui; R. C. Gaba |
| Unique presentations of invasive lobular breast cancer: A case series | 2014 | M. T. Shakoor; S. Ayub; R. Mohindra; Z. Ayub; A. Ahad |
| Unmet needs of chronic hepatitis c in the era of directacting antiviral therapy | 2020 | C. F. Huang |
| Unusual serum protein electrophoresis in a patient with cholangiocarcinoma | 2020 | A. D. T. Bissan; A. Diawara; R. Karfo; A. Teguete; O. Tangara; A. Guindo; F. Maiga; E. Algiman |
| Updates in the systemic treatment of hepatocellular carcinoma | 2018 | E. Y. Chen; C. D. Lopez; G. M. Vaccaro |
| Use heat with caution! Pleurobiliary fistula after hepatocellular carcinoma microwave ablation in lymphoma patient: A case study | 2022 | E. Abdurabuh; M. Khairo; A. Bakhsh; M. Alsharif; W. AlYamani |
| Use of hepatic blood inflow occlusion and hemihepatic artery retention in liver resection for hepatocellular carcinoma | 2016 | C. Jia; C. Dai; X. Zhao; X. Bu; F. Xu; S. Peng; Y. Xu; Y. Zhao; C. Zhao; L. Zhao |
| Use of thromboelastography to demonstrate persistent anticoagulation after stopping enoxaparin | 2007 | R. Simons; S. V. Mallett |
| Usefulness of aspartate aminotransferase to platelet ratio index as a prognostic factor following hepatic resection for hepatocellular carcinoma | 2018 | M. Matsumoto; S. Wakiyama; H. Shiba; K. Haruki; Y. Futagawa; Y. Ishida; T. Misawa; K. Yanaga |
| Usefulness of measuring hepatic functional volume using Technetium-99m galactosyl serum albumin scintigraphy in bile duct carcinoma: Report of two cases | 2009 | A. Nanashima; Y. Sumida; T. Abo; I. Sakamoto; Y. Ogawa; T. Sawai; H. Takeshita; S. Hidaka; T. Nagayasu |
| Utility of AST to platelet ratio index (APRI) score in predicting post-surgical outcomes in patients with cholangiocarcinoma | 2023 | F. Aslam; T. Loveday; P. L. S. Uson; M. J. Borad |
| Utility of hepatobiliary scintigraphy for recurrent reflux cholangitis following choledochojejunostomy: A case report | 2018 | M. Yamamoto; H. Tahara; M. Hamaoka; S. Shimizu; S. Kuroda; M. Ohira; K. Ide; T. Kobayashi; H. Ohdan |
| Utility of remnant liver volume for predicting posthepatectomy liver failure after hepatectomy with extrahepatic bile duct resection | 2021 | R. Yamamoto; T. Sugiura; Y. Okamura; T. Ito; Y. Yamamoto; R. Ashida; K. Ohgi; S. Otsuka; K. Uesaka |
| Validation of the PREsTo machine learning algorithm for the prediction of disease progression in patients with primary sclerosing cholangitis | 2019 | J. Eaton; K. Lazaridis; P. Invernizzi; O. Chazouilleres; G. Hirschfield; H. Metselaar; H. Gronbaek; X. Lu; C. Chung; M. Subramanian; R. Myers; B. McCauley; E. Atkinson; B. Juran; Z. Goodman; M. P. Manns; C. Bowlus; C. Levy; A. Muir |
| Value of Liver Function Tests in Cirrhosis | 2022 | P. Sharma |
| Vanishing bile duct syndrome in a hodgkin's lymphoma patient with fatal outcome despite lymphoma remission | 2013 | A. Aleem; M. Al-Katari; K. Alsaleh; K. Alswat; A. Al-Sheikh |
| Vanishing bile duct syndrome in Hodgkin's lymphoma: A case report and literature review | 2017 | M. Bakhit; T. R. McCarty; S. Park; B. Njei; M. Cho; R. Karagozian; A. Liapakis |
| Vanishing Bile Duct Syndrome: A Rare Cause of Cholestasis in Hodgkin's Lymphoma | 2013 | M. R. De Leon; Y. Bayissa; P. Koduru; S. Velinova; C. Czapar; G. Vettiankal; B. Attar |
| Vascular invasion and lymph node metastasis mediate the effect of CA242 on prognosis in hilar cholangiocarcinoma patients after radical resection | 2022 | G. Heng; B. Huang; Y. Shen; D. wang; Z. Lan; Y. Yao; J. Zhang; J. Jia; C. Zhang |
| Vedolizumab for primary sclerosing cholangitis associated with inflammatory bowel disease: A multicentre cohort study from the GETAID | 2018 | B. Caron; L. Peyrin-Biroulet; M. Nachury; Y. Bouhnik; P. Seksik; G. Bouguen; L. Caillo; D. Laharie; F. Carbonnel; R. Altwegg; C. Reenaers; M. Serrero; A. Boureille; S. Nancey; J. Filippi; V. Abitbol; G. Savoye; L. Vuitton; S. Viennot; M. Fumery; M. Reymond; J. P. Bronowicki; J. M. Reimund; A. Amiot |
| Vimentin-positive circulating tumor cells as diagnostic and prognostic biomarkers in patients with biliary tract cancer | 2021 | S. Y. Han; S. H. Park; H. S. Ko; A. Jang; H. I. Seo; S. J. Lee; G. H. Kim; D. U. Kim |
| Visceral symptoms as a key diagnostic sign for the early infantile form of Niemann-Pick disease type C in a Russian patient: A case report | 2016 | A. V. Degtyareva; S. V. Mikhailova; E. Y. Zakharova; E. L. Tumanova; A. A. Puchkova |
| Vitamin D levels in patients admitted to the intensive care unit and the association with organ dysfunction and glutamine levels | 2020 | F. Seedat; G. K. Schleicher; Gaylard; R. Blaauw |
| Vitronectin (VTN): A Novel Diagnostic and Prognostic Marker for Hepatocellular Carcinoma (HCC) On Top Of Chronic Hepatitis C Virus Related Diseases | 2021 | A. S. Bakir; H. H. Al-Kilany; R. S. Ghait; H. S. Badawy |
| Volumetric ct texture analysis of intrahepatic mass-forming cholangiocarcinoma for the prediction of postoperative outcomes: Fully automatic tumor segmentation versus semi-automatic segmentation | 2021 | S. Park; J. M. Lee; J. Park; J. Lee; J. S. Bae; J. H. Kim; I. Joo |
| Web-based calculator for biliary atresia screening in neonates and infants with cholestasis | 2021 | D. Zhao; S. Gu; X. Gong; Y. Li; X. Sun; Y. Chen; Z. Deng; Y. Zhang |
| Weissella confusa bacteremia in a liver transplant patient with hepatic artery thrombosis | 2010 | N. Harlan; R. Kempker; E. Burd; D. Kuhar |
| Worsening Liver Function Tests in Improving Hypereosinophilic Syndrome Prompting Discovery of Adenocarcinoma With Liver Metastases | 2017 | E. S. John; J. R. Penn; D. N. Seril |
| Wound healing in obstructive jaundice | 1984 | C. P. Armstrong; J. M. Dixon; S. W. Duffy |
| Wrapping double-mattress anastomosis for pancreaticojejunostomy in minimally invasive pancreaticoduodenectomy can significantly reduce postoperative pancreatic fistula rate compared with conventional pancreaticojejunostomy in open surgery: An analysis of a propensity score-matched sample | 2021 | G. Kiguchi; A. Sugioka; Y. Uchida; J. Yoshikawa; M. Nakauchi; M. Kojima; Y. Tanahashi; T. Takahara; A. Yasuda; K. Suda; Y. Kato; I. Uyama |
| YAP activation drives liver regeneration after cholestatic damage induced by Rbpj deletion | 2018 | U. Tharehalli; M. Svinarenko; J. M. Kraus; S. D. Kühlwein; R. Szekely; U. Kiesle; A. Scheffold; T. F. E. Barth; A. Kleger; R. Schirmbeck; H. A. Kestler; T. Seufferlein; F. Oswald; S. F. Katz; A. Lechel |
| Yttrium-90 Radioembolization in the Management of Liver Malignancies | 2010 | A. Riaz; L. M. Kulik; M. F. Mulcahy; R. J. Lewandowski; R. Salem |
| Yttrium-90 Radioembolization of Unresectable Intrahepatic Cholangiocarcinoma: Long-Term Follow-up for a 136-Patient Cohort | 2022 | A. N. Gupta; A. C. Gordon; A. Gabr; A. Kalyan; S. M. Kircher; D. Mahalingam; M. F. Mulcahy; R. P. Merkow; A. D. Yang; D. J. Bentrem; J. C. Caicedo-Ramirez; A. Riaz; B. Thornburg; K. Desai; K. T. Sato; E. S. Hohlastos; L. Kulik; A. B. Benson; R. Salem; R. J. Lewandowski |
| Yttrium-90 Radiotherapy for Unresectable Intrahepatic Cholangiocarcinoma: A Preliminary Assessment of This Novel Treatment Option | 2010 | A. Saxena; L. Bester; T. C. Chua; F. C. Chu; D. L. Morris |
